# Molecularly Imprinted Polymers for Selective Extraction of Oblongifolin C from *Garcinia yunnanensis* Hu

**DOI:** 10.3390/molecules22040508

**Published:** 2017-03-23

**Authors:** Liping Wang, Wenwei Fu, Yunhui Shen, Hongsheng Tan, Hongxi Xu

**Affiliations:** 1 School of Pharmacy, Shanghai University of Traditional Chinese Medicine, Shanghai 201203, China; maxine_wlp0411@163.com (L.W.); fu_wenwei@163.com (W.F.); bravesyh@163.com (Y.S.); ths97029@163.com (H.T.); 2 Engineering Research Center of Shanghai Colleges for TCM New Drug Discovery, Shanghai 201203, China

**Keywords:** molecularly imprinted polymers, solid-phase extraction, oblongifolin C, guttiferone K, *Garcinia yunnanensis* Hu

## Abstract

Molecularly imprinted polymers (MIPs) were synthesized and applied for the selective extraction of oblongifolin C (OC) from fruit extracts of *Garcinia yunnanensis* Hu. A series of experiments and computational approaches were employed to improve the efficiency of screening for optimal MIP systems in the study. The molar ratio (1:4) was eventually chosen based on the comparison of the binding energy of the complexes between the template (OC) and the functional monomers using density functional theory (DFT) at the RI-PBE-D3-gCP/def2-TZVP level of theory. The binding characterization and the molecular recognition mechanism of MIPs were further explained using the molecular modeling method along with NMR and IR spectra data. The reusability of this approach was demonstrated in over 20 batch rebinding experiments. A mass of 140.5 mg of OC (>95% purity) was obtained from the 5 g extracts, with 2 g of MIPs with the best binding properties, through a gradient elution program from 35% to 70% methanol-water solution. At the same time, another structural analog, 46.5 mg of guttiferone K (GK) (>88% purity), was also obtained by the gradient elution procedure. Our results showed that the structural analogs could be separated from the crude extracts by the molecularly imprinted solid-phase extraction (MISPE) using a gradient elution procedure for the first time.

## 1. Introduction

*Garcinia yunnanensis* (Guttiferae) Hu is an evergreen tree found in the southwestern part of the Yunnan province in China [1]. The fruits of this plant are called “Xiaoguliangguo” by local people, and are edible, like the fruits of the other plants in the genus, such as *G. mangostana*, *G. subfalcata*, *G. esculenta*, and *G. indica* [2]. Previous phytochemical investigations in our group showed that oblongifolin C (OC) and guttiferone K (GK) are the two main polyisoprenylated benzophenone derivatives (PPAPs) from *G. yunnanensis* Hu [1]. Our previous studies further revealed that OC and GK are multi-therapeutic agents with anti-inflammatory and anti-cancer properties [1,3,4,5,6,7]. Emerging evidence clearly indicated that OC and GK are promising candidates for developing novel treatments for infection or cancer, thus large amounts of these compounds are needed for further investigations, especially for animal studies [5]. Without a published synthetic route, OC or GK was usually isolated from plants using conventional techniques [1,8], such as repeated silica gel column chromatography, MCI column chromatography, medium-pressure preparative liquid chromatography (MPLC), and preparative HPLC, all of which included complicated, inefficient, and time-consuming operations. Thus, an efficient method to selectively extract OC or GK should be established.

Molecular imprinting, developed by Wulff and Sarhan in 1972, is a technology that facilitates the production of artificial receptors toward compounds of interest [9]. Molecularly imprinted polymers (MIPs) are functionally porous materials with stereospecific, three dimensional binding cavities for recognition of a particular target molecule. MIPs have many advantages, including high selectivity, mechanical and chemical stability, low cost and easy preparation, and a long storage life. Over the past decades, these polymers have been successfully used in different fields [10,11,12,13,14,15] such as chemical sensors [16,17,18,19,20], enzyme mimicking catalysis [21,22,23], intelligent drug delivery [24,25,26,27], etc. In particular, MIPs have been extensively employed as the adsorbents of the solid phase extraction (SPE) for the selective extraction of target compounds and structural analogues from complex matrices, such as natural products [28,29,30,31,32]. However, little research focuses on the isolation of structural analogues with the same polar functional groups and a similar molecular volume, from the complex matrices by using only the molecularly imprinted solid-phase extraction (MISPE) technique. On the other hand, the screening of MIP formulations is still a tedious and time-consuming process. PPAPs-imprinted MIP systems have rarely been explored computationally due to the high molecular weight and the flexible structure. Although, many applications of computational approaches in molecular imprinting have demonstrated the potential of molecular modeling toward rational MIP design.

In this manuscript, we reported a simple technique to selectively extract OC from the fruit extracts of *G. yunnanensis* Hu using PPAPs-imprinted MIPs. Computational approaches were applied to determine optimal ratios of the template to functional monomer for polymer formation. Batch rebinding experiments were performed to evaluate the MIPs performance. The adsorption process and its kinetics were investigated, and the adsorption process could be explained well by the *Freundlich* isotherm model. The MIPs with the best binding properties were successfully applied to MISPE for the selective extraction of OC from fruit extracts of the plant. At the same time, another structural analog, GK, was also obtained by the gradient elution procedure. To the best of our knowledge, the structural analogs could be separated from the crude extract by the MISPE using a gradient elution procedure for the first time.

## 2. Results and Discussion

### 2.1. Comparative Study of Different Polymer Preparation Conditions

Optimization of the polymer composition is a crucial step in polymer preparation. The typical bulk polymerization was chosen as the polymerization method. Eleven different polymers were prepared using different functional monomers, porogens, or molar ratios among molecular templates, functional monomers and crosslinkers, as shown in Table 1. Batch rebinding studies were conducted to evaluate the binding efficiency of the polymers prepared using different conditions. The imprinting factor (IF) was calculated for each MIPs to determine the degree of specificity of its binding sites using Equation (3). Table 1 showed the list of the IF values that were obtained for the eleven polymers.

The selection of a suitable functional monomer is crucial because the weak interaction between the template and functional monomer affects the efficiency of the finally obtained MIPs in non-covalent imprinting. As shown in Table 1, the results revealed that the MIPs prepared with AM as the functional monomer exhibited the best recognition ability with the highest IF value (MIPs A–C in Table 1).

The imprinting efficiencies of the MIPs prepared in four different porogens were investigated. Table 1 showed that MIPs prepared in the DMSO had higher IF values than those prepared using other porogens (MIPs C–F in Table 1). The best imprinting effect was obtained at a molecular template:functional monomer:crosslinker ratio of 1:4:10 as shown in Table 1 (MIPs C, G–K in Table 1).

### 2.2. Molecular Modeling and Computational Optimization

The selection of a suitable functional monomer and its molar ratio is a time consuming stage in the development process of MIPs. Computational modeling has become a versatile tool for reducing the time of experiments and for studying the molecular recognition mechanism of the MIPs.

In the computational studies, AM was chosen as the functional monomer, as it exhibited good retention of OC in the batch rebinding studies. It is partly because AM is a basic monomer, thus is favorable for interactions between acidic and basic functional groups, such as the formation of hydrogen bonds that are often found. The template-monomer complex structures were drawn with Discover Studio 2.5 software (Accelrys, San Diego, CA, USA). The weak hydrogen bonds, pi–pi, and pi–sigma interactions between template and monomers clusters were monitored in the software, and only the hydrogen bonds were monitored. Thus, the complex level was mainly determined using the number of intermolecular hydrogen bonds formed between the functional monomers and the template.

**Figure 1:**
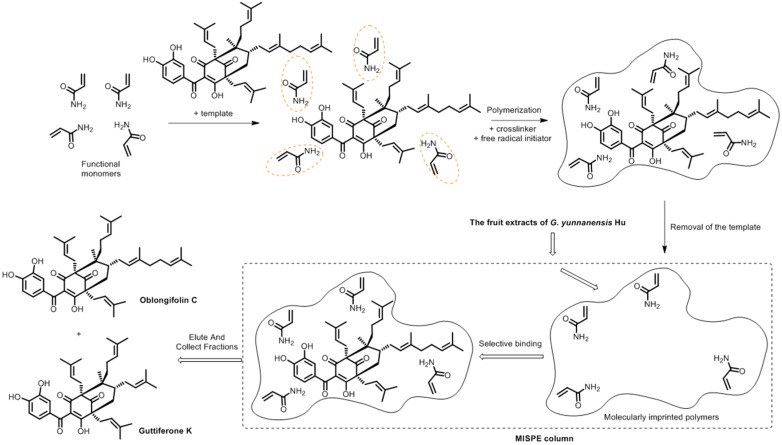
Schematic procedure for the preparation of MIPs and its application to the herbal extracts.

There are three hydrogen-donating and three hydrogen-accepting sites in OC. Due to the intramolecular hydrogen bonding observed, it seemed that there were only four sites that could be offered to the monomers. Figure 1 illustrated the expected mechanism of polymerization between OC and AM at the four sites. Thus, four templates to functional monomer molar ratios, which were 1:1, 1:2, 1:3, and 1:4, were screened for all possible interactions between the functional monomer and the template molecule in the DMSO.

The binding energies of the complexes formed in the solvent phase (DMSO) were calculated according to the Equation (1) (shown in Section 3.3) and the Boltzmann distribution rate of each complex geometric conformation at 300 K, was summarized in Table 2. The obtained results showed that the calculated binding energies decreased by increasing the number of monomers used, and the lower the value of the binding energy, the more stable the formation of the complex. Thus, the best molar ratio for polymer preparation was 1:4, providing the MIPs with good recognition properties [32,33].

**Figure 2:**
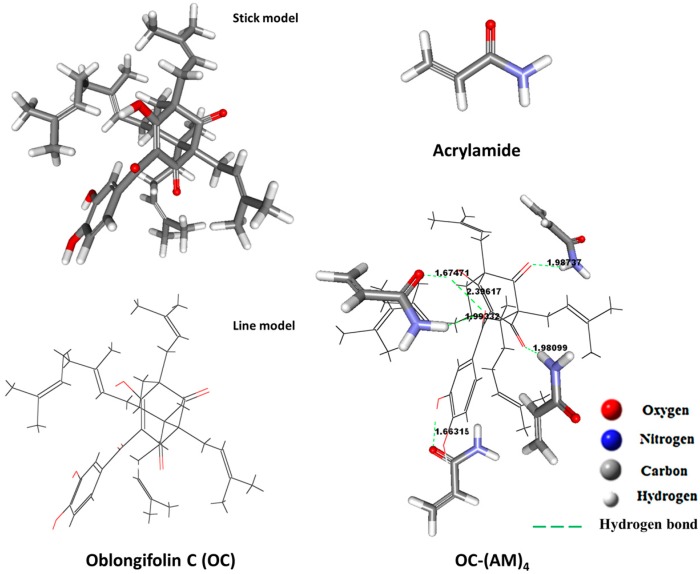
The best conformations of AM, OC and OC–(AM)4 complex.

Figure 2 represented the best conformations of OC, AM and the 1:4 complex. Moreover, the bond distances between the hydrogen atom of the OH-group or NH_2_-group and the oxygen atom of the carbonyl group at the different sites were calculated. They all ranged between 1.66 and 2.39 Å which is within the hydrogen bond range as indicated in Figure 2 [32,34]. Thus, hydrogen bonding is the only possible contributor in the stabilization of the pre-polymerization complex [35].

### 2.3. Characterization of the Morphology

The FTIR spectra of OC, unleached MIPs, leached MIPs, and non-imprinted polymers (NIPs) were recorded in the range of 4000 to 500 cm^−1^ as shown in Figure S1. The similarity in these FTIR spectra indicated that these polymers or OC had similar groups. For example, a strong peak at ~1731 cm^−1^ ascribed to the stretching vibration of –C=O in the EDGMA (crosslinker), AM (functional monomer) or OC (template), and a strong peak at ~3400 cm^−1^ ascribed to the stretching vibration of –NH_2_ groups in AM or the stretching vibration of –OH groups in OC. The IR spectra confirmed the successful incorporation of OC in MIPs. Compared with the peaks at ~3450 cm^−1^ and ~2960 cm^−1^ in the leached MIPs and NIPs spectra, the broader peaks at ~3450 cm^−1^ in the FTIR spectra of unleached MIPs could be assigned to the stretching vibration of –OH in OC, and the broader peaks at ~2960 cm^−1^ could be assigned to the stretching vibration of –CH_3_ in OC.

The NMR spectra of OC, AM and OC + AM were recorded as shown in Figures S2–S4. The interaction between the functional monomer and template molecule was also investigated using the ^1^H-NMR spectra. The electron density around a proton decreased when it participated in hydrogen bonding, which caused the proton in the hydrogen bond to shift downfield. Moreover, the larger the change of the chemical shift, the stronger the interaction between the template and monomer. Compared with the –OH proton peak at 9.912 ppm in the OC ^1^H-NMR spectrum, the –OH protons were shifted downfield to 9.916 ppm in the ^1^H-NMR spectrum of the mixture (OC:AM = 1:4, mol:mol; Figure S2). Compared to the –NH_2_ proton peak at 7.509 ppm in the acrylamide ^1^H-NMR spectrum, the –NH_2_ protons were shifted downfield to 7.513 ppm in the ^1^H-NMR spectrum of the mixture (OC:AM = 1:4, mol:mol; Figure S3). The –C=O carbon was shifted downfield from 166.33 to 166.35 ppm in the ^13^C-NMR spectrum of the mixture (OC:AM = 1:4, mol:mol) compared to the –C=O carbon peaks in the acrylamide (Figure S4). These results confirmed the strong hydrogen bonding interactions between OC and AM.

SEM was used to illustrate the detailed morphology of the MIPs and the corresponding NIPs as shown in Figure S5. We found that the rough and irregular particles of both polymers were constructed as network structures, which was mainly due to use of the bulk polymerization method. However, such irregularity in our study did not have a significant effect on the binding properties of the imprinted polymer, as previously reported for bulk polymers [32]. The morphology of the best polymer was further investigated using BET N_2_ adsorption–desorption analysis. Comparing the imprinted and the non-imprinted polymer, we found that MIPs C showed a higher specific surface area than NIPs C (94.253 and 86.311 m^2^·g^−1^, respectively), as well as a higher pore volume (0.147 and 0.129 cc·g^−1^, respectively) and a higher pore diameter (6.247 and 5.996 nm, respectively). These results directly indicated a significant influence of the template on the polymer structure, where the presence of imprinted cavities increased the surface area and provided a higher number of selective binding sites.

### 2.4. Evaluation of the Adsorption Properties

#### 2.4.1. Adsorption Isotherm

Since MIPs C showed the highest binding capacity and efficiency, as shown in Table 1, the static adsorption properties of MIPs were investigated using the equilibrium absorption of MIPs and NIPs toward OC. The relationship of the adsorption capacity to the concentration of OC was shown in Figure S6a. Notably, the capacity of MIPs for OC increased observably after raising the initial concentrations (0.5 to 2.0 mM), and then the adsorption capacity for NIPs was apparently lower than that of MIPs at the same concentration. This finding indicated that the MIPs showed an observably higher capacity than the NIPs.

The adsorption mechanism of the MIPs could be explained using two models: the one binding site model described by the Langmuir equation and the continuous distribution model described by the *Freundlich* equation [36,37]. The binding data for MIPs and NIPs toward OC were further processed according to the *Langmuir* and *Freundlich* equations, and the results were shown in Figure S7. The correlation coefficients (*R*^2^) of the *Langmuir* and *Freundlich* equations of MIPs were 0.6115 and 0.981, respectively, and the correlation coefficients (*R*^2^) of the *Langmuir* and *Freundlich* equations of NIPs were 0.2216 and 0.9528, respectively. Therefore, the *Freundlich* isotherm model was more suitable to the adsorption process due to the higher correlation coefficient (*R*^2^). According to the *Freundlich* theory, the adsorption processes of MIPs and NIPs toward OC were mostly likely explained by the multiple adsorption attributed to the heterogeneous surfaces in the binding site. The KF and n values in the *Freundlich* equation were calculated according to the slopes and intercepts of the two linear regression equations. In the current study, the KF value was calculated to be 0.102 mmol·L^−1^ in the MIP *Freundlich* equation for the higher affinity binding sites in the MIPs and was 0.038 mmol·L^−1^ in the NIP equation for the lower affinity binding sites in the NIPs. The n value was calculated to be 1.117 in the MIP *Freundlich* equation and was 0.879 in the NIP equation, indicating that the absorption of MIPs for OC was favorable (*n* > 1) and the absorption of NIPs for OC was not suitable (*n* < 1) according to the *Freundlich* theory.

#### 2.4.2. Adsorption Kinetics

The adsorption kinetics of MIPs and NIPs toward OC were examined. The adsorption equilibrium was achieved after 30 min for MIPs C, while it did not reach equilibrium in more than 120 min for NIPs C, as shown in Figure S6b. The mass transfer rate of OC onto MIPs C was very fast and may have resulted from the preferential and rapid adsorption of the template onto the binding sites. The results indicated that the MIPs could be used to the selective extraction of OC in practice.

#### 2.4.3. Selectivity Study of the Sorbents

**Figure 3:**
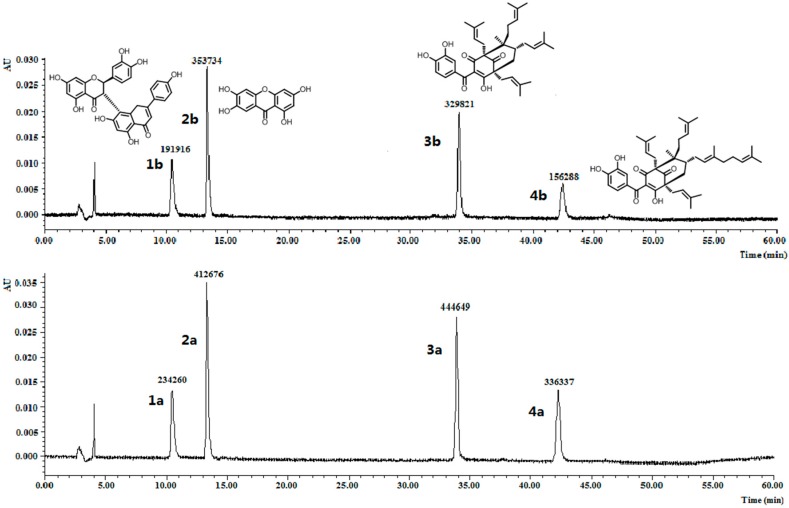
Chromatograms for the selectivity experiment. The peak areas of volkensiflavone (**1a** and **1b**), 1,3,6,7-tetrahydroxyxanthone (**2a** and **2b**), GK (**3a** and **3b**) and OC (**4a** and **4b**) before and after incubation with 10 mg of MIPs C were showed.

To further investigate the molecular recognition properties of the MIPs, 10 mg of the polymers were incubated with 2 µM of OC and its structural analogues. The analogues chosen, volkensiflavone (1), and 1,3,6,7-tetrahydroxyxanthone (2), occur naturally in the plants from the *Garcinia* genus, and GK (3) coexists with OC (4) within the fruits exacts of *G. yunnanensis* Hu. The results summarized in Figure 3 clearly showed that MIPs C distinguished OC and GK from other analogues, whose equilibrium concentration was relatively reduced by more than double by volkensiflavone (1) and 1,3,6,7-tetrahydroxyxanthone (2). MIPs C had the higher affinity towards OC and GK, and had the highest affinity towards OC. Therefore, the MIPs could be a selective absorbent for OC and structurally similar compounds such as GK, and could be used to separate and purify these compounds from *Garcinia* extracts.

### 2.5. Optimization of the MISPE Procedures

For application in real samples, some procedures of the MISPE were investigated further, such as the type of the loading solvent, the composition and volume of the eluting solvent, and the composition of the washing solvent. All experiments used SPE columns packed with 1.0 g MIPs.

#### 2.5.1. The Type and Volume of the Loading Solvent

As the first step toward the development of an effective MISPE method, the type and volume of the loading solvent should be determined. The choice of loading solvent is important because it affects the interactions between the template and the MIPs. In this experiment, several loading solvents were tested. As shown in Table S2, although the MIPs had higher binding rates in the solution of methanol–water (70:30, *v/v*) than in the other solvents, the solubility was poorer compared to the other solvents. Ultimately, the solution of methanol–water (80:20, *v/v*) was selected as the loading solvent.

As shown in Table S3, OC from 1 g fruit extracts of G. yunnanensis Hu could be dissolved completely (99.7%) in 5 mL of methanol–water (80:20, *v/v*), indicating that the appropriate loading solvent volume is five times the weight of the extracts. According to the binding isotherm assay described earlier, the maximum adsorption capacity of MIPs was estimated more than 0.14 mmol·g^−1^ (Q > 0.14 mmol·g^−1^), which corresponded to 94 mg·g^−1^. Moreover, the binding rates of GK observably decreased when the extracts weight increased (Table S4), indicating that different amounts of GK and OC were obtained through an adjustment of the mass ratio of loading amount of extracts to amount of MIPs.

#### 2.5.2. Washing and Eluting Solvent Selection

Based on the results in Section 2.5.1, the methanol–water solution was selected as the appropriate washing solution system. As shown in Figure S8, only GK was obtained from the eluate when methanol–water (50:50, *v/v*) was used as the eluting solution, and OC was obtained from the eluate when the 70:30 (*v/v*) solution of methanol–water was used as the eluting solvent. The gradient elution method involved the following three steps: (1) methanol–water (35:75, *v/v*); (2) the separation of GK with methanol–water (50:50, *v/v*); and (3) the separation of OC with methanol–water (70:30, *v/v*). This experiment was the first to use a gradient elution method for the separation of OC and GK based on MISPE.

#### 2.5.3. Reusability of MISPE

The reusability of MISPE was also investigated. After each test, the cartridge was rinsed with methanol and water in sequence, and subsequently it was reused for the next test cycle. As shown in Table S5, the relative binding rate decreased to 70% after 20 cycles; however the binding rate increased to 92% in the 21st experiment after the cartridge was rinsed with methanol–acetic acid (90:10, *v/v*) and methanol in sequence, demonstrating the reusability of the MISPE procedure.

### 2.6. Application of MISPE to Fruit Extracts of G. yunnanensis Hu

**Figure 4:**
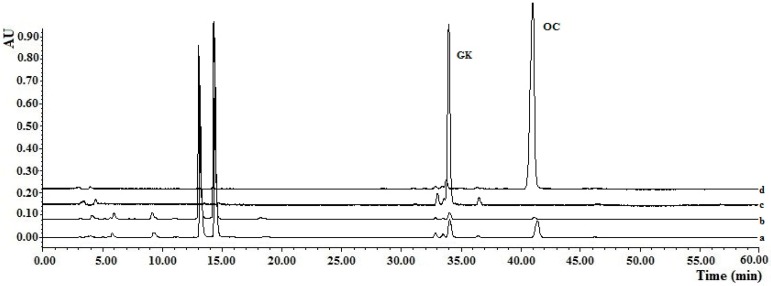
Chromatograms for molecularly imprinted solid-phase extraction (MISPE) using the gradient elution method: (**a**) Chromatograms of the fruit extracts solutions; (**b**) Chromatogram of elution fractions from the OC-MISPE column after gradient elution (Methanol–water, 35:65, *v/v*); (**c**) Chromatogram of elution fractions from the OC-MISPE column after gradient elution (Methanol–water, 50:50, *v/v*); (**d**) Chromatogram of elution fractions from OC-MISPE column after gradient elution (Methanol–water, 70:30, *v/v*).

For complete confirmation of the practicability of the OC-MISPE column, an experiment was conducted using the OC-MISPE column with 0.25 g, 1.25 g or 2.0 g of MIPs (Table S6). As shown in Figure 4, there was a significant decrease in the content of the OC and GK compared to those of the other compounds in the fruit extracts of *G. yunnanensis* Hu after incubation with MIPs.

The results showed that 140.5 mg of OC with a 95% purity and 46.5 mg of GK with an 88% purity were acquired from a 5 g extracts with 2 g of MIPs. The recoveries of OC in our work were in the range of 48.0%–77.7% and the recoveries of GK were in the range of 25.1%–32.4%. Although there were low recoveries in our work, the amount of targets from the same amount of crude extracts was much higher than that in Ref. [1] (Table S6). All of this data demonstrated that the proposed method was a suitable technology for the selective extraction of OC and GK from the fruit extracts of *G. yunnanensis* Hu.

## 3. Materials and Methods

### 3.1. Reagents and Materials

OC (purity > 97%) and GK (purity > 97%) were obtained in the laboratory from the fruits of *G. yunnanensis* Hu as described in the literature [1]. Ethylene glycol dimethacrylate (EDGMA, 99%), 2,2’-azobisisobutyronitrile (AIBN, 99%), acrylamide (AM, 99%), methacrylic acid (MAA, 99%), and 4-vinylpyridine (4-VP, 99%) were purchased from J & K Scientific (Shanghai, China). Acetic acid, dimethylsulfoxide (DMSO), chloroform (CHCl_3_), toluene (Tol.), HPLC-grade methanol (MeOH), acetic acid (HOAc), and acetonitrile (MeCN) were purchased from the Sinopharm Chemical Reagent Co. (Shanghai, China). All solvents were of analytical grade unless noted otherwise. All of the experiments used Milli-Q system-purified deionized water. The 0.22 μm syringe driven filters were purchased from Anpel Laboratory Technologies Inc. (Shanghai, China).

The fruits of *G. yunnanensis* Hu were collected from the Dehong prefecture, Yunnan province in 2014, and the species was identified by Dr. HongMei Zhang (School of Pharmacy, Shanghai University of Traditional Chinese Medicine, Shanghai, China).

### 3.2. Apparatus

The FTIR spectra analysis was conducted on a Nicolet FTIR 6700 spectrometer. NMR spectra were measured on a Bruker AV-400 spectrometer and were calibrated based on the solvent peak. The scanning electron microscope (SEM) images of the surface morphology of imprinted and non-imprinted polymers were recorded on a field emission scanning electron microscope with an Oxford INCA 350 Energy dispersive X-ray microanalysis system (Hitachi S-4800, Hitachi Ltd., Tokyo, Japan). The specific surface areas and pore volumes were determined using a Physisorption Analyzer (ASAP 2020 series, Micromeritics Instrument, Norcross, GA, USA). The tests were performed at the Center for Pharmaceutical Analysis and Solid-State Chemistry Research (Shanghai Institute of Materia Medica, Chinese Academy of Sciences, Shanghai, China).

All chromatographic measurements were performed using an HPLC or a UHPLC instrument. All of the analyses were carried out in triplicate. HPLC experiments were implemented using a Waters 2535 series high-performance liquid chromatographic instrument (Waters Corp., Framingham, MA, USA). The analytical column was a Shim-pack VP-ODS column (4.6 × 250 mm, 4.5 μm, Shimadzu, Tokyo, Japan). UHPLC analyses was performed on a Waters ACQUITY UPLC H-class system equipped with a quaternary solvent manager, a sample manager-FTN, and a PDA detector (Waters Corp., Framingham, MA, USA) connected to a reversed-phase column (Waters Acquity UPLC BEH Shield RP C18, 100 mm × 2.1 mm, i.d., 1.7 μm). The analytical conditions and methodology validations for HPLC and UHPLC analyses were described in the experimental section of the supplementary materials (See Table S1).

### 3.3. Computational Optimization: Monomers Molar Ratio Screening

For a rational design of the MIPs with OC as the template molecule, the molecular modeling method was applied. Monte Carlo conformational searching was performed for the low energy conformations (<5 kcal/mol from global minimum) of template (OC) and functional monomer (AM) using Macromodel v10.0 [38] (Schrodinger Inc., Portland, OR, USA). The template-monomer complex structures were drawn using Discover Studio 2.5 software, and the template, functional monomer and clusters of template-monomer complex conformations were further optimized using MOPAC2012 with the PM7 quantum chemical semi-empirical method [39]. The single-point energies were calculated via density functional theory (DFT) methods, and were implemented in the ORCA 3.03 package [40] at the RI-PBE-D3-gCP/def2-TZVP level. Four template to monomer molar ratios were screened, 1:1, 1:2, 1:3, and 1:4.

A conductor-like screening model (COSMO) [38] was applied to evaluate the effect of the solvent on the binding energy calculations. Finally, the binding energy of each complex was calculated as follows Equation (1):

∆E_binding_ = E_complex_ − [E_OC_ + nE_AM_]. (1)

In the equation, ∆E_binding_ is the binding energy between the OC and AM, and n refers to the monomer number in the template-monomer complexes. E_complex_, E_OC_, and E_AM_ are the single-point energies of OC–AM complexes, OC, and AM, respectively.

### 3.4. Preparation of MIPs

MIPs were prepared using a non-covalent approach with bulk polymerization. Eleven MIPs for OC were prepared (A-K), where 0.125 mmol of the template molecule (OC) was dissolved in 3 mL of porogen (DMSO, MeCN, Tol., CHCl_3_) in a 10 mL glass tube. The functional monomer (AM, MAA, 4-VP) was then added, and the pre-polymerization solution was shaken for 1.5 h. Next, the solution was mixed with the crosslinker (EGDMA) for 10 min. Then, 10.0 mg of the free radical initiator (AIBN) was added and the solution was purged with argon for 10 min. Finally, the glass tube was sealed and placed at 60 °C in a water bath for 24 h for polymerization to take place. For each MIP, corresponding NIPs were prepared by exactly the same procedure as described above, but without the OC template. The chemical composition of the pre-polymerization mixture was described in Table 1.

The obtained bulk polymers that had a yellow lumpy structure were crushed, ground, and sieved through a 35–45 μm stainless steel mesh. The sieved particles were washed with a mixture of methanol–acetic acid (90:10, *v/v*) using a soxhlet apparatus until no template was detected by HPLC in the extract and then washed with methanol until neutral. The synthesized polymers were dried in a vacuum drying oven at 60 °C until a constant weight was achieved and then stored at 4 °C for the following experiments.

### 3.5. Evaluation of MIPs Performance

The imprinting efficiency of the prepared polymers was determined using three different factors: the binding capacity, adsorption kinetics, and selectivity. These factors were evaluated by performing equilibrium batch rebinding experiments.

#### 3.5.1. Equilibrium Batch Rebinding Experiments

All of the MIPs and their corresponding NIPs (10 mg) were incubated with 1 mL of OC solution at a concentration of 1.5 mmol·L^−1^ in a methanol–water solution (70:30, *v/v*). After equilibration for 2 h at room temperature, the suspensions were centrifuged at 13,800 rpm for 1 min. The supernatant liquid was filtered through a 0.22 µm syringe driven filter. The concentration of the free OC was then quantified using the UHPLC technique. The adsorption capacity was calculated using the following Equation (2) [37]:

Q_f_ = (C_i_ − C_f_) v*/*m (2)

where C_i_ (µg·mL^−1^) and C_f_ (µg·mL^−1^) are the initial and final concentrations of OC, respectively, and v is the total volume of solution in mL, and m is the mass of the polymer added in mg.

The imprinted factors were obtained following the Equation (3):

IF = Q_MIPs_/Q_NIPs_. (3)

#### 3.5.2. Adsorption Isotherms

To investigate the static binding ability of MIPs and NIPs, 20 mg of the polymers were mixed with 1.5 mL of 97% pure OC solutions in methanol−water (70:30, *v/v*) over a concentration range of 0.5 to 2.0 µmol·mL^−1^. After equilibration for 2 h at room temperature, the free and bound OC concentrations were quantified following the same procedure as mentioned earlier. The data from the batch rebinding experiments were further processed according to the following *Langmuir* and *Freundlich* Equations (4) and (5) [36,37,41]:

C_f_/Q_f_ = 1/(q_m_·K_b_) + C_f_/q_m_ (4)

ln Q_f_ = 1/*n* ln C_f_ + ln K_F_. (5)

where K_b_ and q_m_ are the affinity constant and binding site density, respectively, of the *Langmuir* equation, and K_F_ and 1/n are the maximum adsorption capacity and heterogeneity parameter, respectively of the *Freundlich* equation. Moreover, n can be used to determine whether the adsorption is favorable, where 10 > *n* > 1 is a favorable adsorption.

#### 3.5.3. Adsorption Kinetics

The kinetic study was carried out with 20 mg of MIPs or NIPs and a 1 mL solution of OC at a concentration of 1.5 mmol·L^−1^ in a methanol–water solution (70:30, *v/v*).

#### 3.5.4. Selectivity Experiments

The selectivity was examined by comparing the binding capacity of the best MIPs to four compounds (including OC) that naturally occur in plants from the Garcinia genus. For this purpose, 10 mg of the imprinted polymers were equilibrated with volkensiflavone (1, 0.90 µmol·mL^−1^), 1,3,6,7-tetrahydroxyxanthone (2, 1.92 µmol·mL^−1^), GK (3, 1.66 µmol·mL^−1^), and OC (4, 1.49 µmol·mL^−1^) for 3 h using the same procedure as discussed earlier. The bound amount of the four different compounds was then quantified using the same HPLC method.

#### 3.5.5. Characterization of the Molecularly Imprinted Polymer

Fourier transform infrared (FTIR) spectra for the powdered OC, the best MIPs before and after leaching, and their corresponding NIPs were recorded in the range of 500 to 4000 cm^−1^. The surface morphology of the best MIPs and their corresponding NIPs were examined using scanning electron microscopy (SEM). The surface area and the pore volume were analyzed using the Brunauer–Emmett–Teller (BET) method. The polymers were first degassed for 4 h at 100 °C to remove absorbed gas and moisture. The surface area and the pore volume were then determined using N_2_ absorption/desorption analysis [42,43].

### 3.6. Preparation of MISPE Column

**Figure 5:**
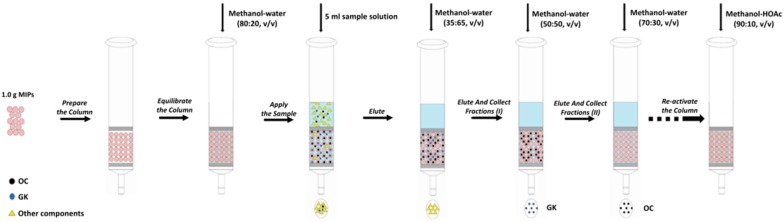
Schematic diagram of the generic procedure of MISPE.

Next, 1.0 g of MIPs was packed into a solid phase extraction cartridge. At first, the polymers were pre-equilibrated with methanol–water (70:30, *v/v*). After the preparation of the MISPE column, the steps of loading, washing and elution vary slightly according to the different experimental conditions (see Section 2.5.1 and Section 2.5.2). The flow rate was set at 0.1 mL·min^−1^ and the filtrate was collected and filtered by a 0.22 μm filter for HPLC analysis. The generic procedure of MISPE was illustrated in Figure 5.

### 3.7. Preparation of the Fruit Extracts of G. yunnanensis Hu

A total of 45 g of the powder of *G. yunnanensis* Hu fruits was refluxed with 95% ethanol for 2 h (225 mL × 3). The filtrate was concentrated in a vacuum to accommodate the crude extract (23.3 g). The obtained fruit extracts of *G. yunnanensis* Hu were dissolved in the selected loading solution (methanol–water (80:20, *v/v*)) and applied to the MISPE column. The amount of OC bound was quantified using the same HPLC method described in supplementary materials. The recovery of purified OC was calculated by the mass ratio of purified amount of OC to total amount of OC in fruit extracts. The same goes for the recovery of purified GK.

## 4. Conclusions

For the first time, MIPs were successfully developed for the selective isolation of two structurally similar compounds, OC and GK, from fruit extracts of *G. yunnanensis* Hu using a gradient elution method in a single run. Based on the computational analysis and the results of batch rebinding experiments, MIPs that were prepared using bulk polymerization with AM as the functional monomer and EDGMA as the crosslinker with a molar ratio of molecular template:functional monomer:crosslinker (1:4:10) showed the best binding properties.

The molecular recognition mechanism is mainly based on intermolecular hydrogen binding between the OC and the MIPs, as determined using the molecular modeling method along with IR and NMR spectra data. The MIPs showed robust and selective binding to template molecule (OC) and analogue (GK), and the gradient elution method in the MISPE can be used to separate structurally similar chemical compounds from the herbal extracts in a single run.

## Figures and Tables

**Table 1:** Optimization of the preparation of molecularly imprinted polymers (MIPs) for oblongifolinC (OC).

MIPs	Molecular Template ^1^	Functional Monomer	Crosslinker	Porogen	Molar Ratio ^2^	IF ^3^	RSD ^4^ (%)
A	OC	4-VP	EDGMA	DMSO (3 mL)	1:4:10	1.26	6.67
B	OC	MAA	EDGMA	DMSO (3 mL)	1:4:10	1.37	1.25
C	OC	AM	EDGMA	DMSO (3 mL)	1:4:10	3.42	0.42
D	OC	AM	EDGMA	MeCN (3 mL)	1:4:10	0.87	0.76
E	OC	AM	EDGMA	Tol. (3 mL)	1:4:10	1.36	6.66
F	OC	AM	EDGMA	CHCl_3_ (3 mL)	1:4:10	0.93	4.98
G	OC	AM	EDGMA	DMSO (3 mL)	1:4:8	2.34	8.03
H	OC	AM	EDGMA	DMSO (3 mL)	1:4:20	1.10	3.41
I	OC	AM	EDGMA	DMSO (3 mL)	1:1:10	1.93	0.78
J	OC	AM	EDGMA	DMSO (3 mL)	1:2:10	1.95	1.17
K	OC	AM	EDGMA	DMSO (3 mL)	1:3:10	2.08	0.73

^1^ Molecular template = 0.125 mmol. ^2^ Molar ratio: MT = molecular template; FM = functional monomer; CL = crosslinker. ^3^ Tests were performed in triplicate (*n* = 3). ^4^ RSD = relative standard deviation.

**Table 2:** The calculated binding energies of the complexes prepared in the solvent phase (DMSO).

Complexes	Energies (Hartree) ^1^	Binding Energies (kcal·mol^−1^) ^2^
OC (Template)	−2122.941	/
AM	−247.118	/
OC-AM	−2368.641	−2.343
OC-(AM)2	−2609.601	−8.594
OC-(AM)3	−2843.979	−13.128
OC-(AM)4	−3110.504	−15.465

^1^ hartree = 627.5 kcal. ^2^ The binding energies were finally obtained by weighing the Boltzmann distribution rate of each complex geometric conformation at 300 K. Cartesian coordinates and single point energies of the computed complex geometric conformations could be found in Part 3 of the Supplementary Material.

## Supplementary Materials

Supplementary materials are available online.
