# Supplementary material for: Molecularly Imprinted Polymers for Selective Extraction of Oblongifolin C from Garcinia yunnanensis Hu
Source: Molecules. 2017 Mar 23;22(4):508. doi: 10.3390/molecules22040508 (PMC6153995; doi:10.3390/molecules22040508)
Supplement: Supplementary file 1 [file molecules-22-00508-s001.pdf]

Supplementary materials

Molecularly imprinted polymers for selective extraction of oblongifolin C from *Garcinia yunnanensis* Hu

Liping Wang [1,2,a], Wenwei Fu [1,2,a], Yunhui Shen [1], Hongsheng Tan [1,2], and Hongxi Xu[1,2 *]

[1] School of Pharmacy, Shanghai University of Traditional Chinese Medicine, Shanghai 201203, China; maxine_wlp0411@163.com; fu_wenwei@163.com; bravesyh@163.com; ths97029@163.com

[2] Engineering Research Center of Shanghai Colleges for TCM New Drug Discovery, Shanghai 201203, China

[*] Correspondence: xuhongxi88@gmail.com; Tel./Fax: +86-21-51323089

[a] The two authors contributed equally to this work and should be considered as co-first authors.

List of supplementary materials

Part 1. Experimental section……………………………………………………………..3

Method validation of the HPLC and UHPLC analyses.

Part 2. Figures and Tables ………………………………………………………………5

Figure S1. FTIR spectra of OC, NIPs, leached MIPs, and unleached MIPs.

Figure S2. ^{1}H NMR in DMSO-*d* of OC and OC + AM.

Figure S3. ^{1}H NMR in DMSO-d of AM and OC + AM.

Figure S4. ^{13}C NMR in DMSO-d of OC, AM and OC + AM.

Figure S5. SEM images of MIPs C and NIPs C.

Figure S6. Evaluation of the adsorption properties.

Figure S7. *Langmuir* and *Freundlich* isotherms for adsorption of OC on MIPs and NIPs.

Figure S8. Chromatograms for elution solvent selection.

Table S2. Binding rates of different loading solvents on the static adsorption.

Table S3. Solution efficiency of different volumes of the loading solution

Table S4. Binding rates of different loading amount of extracts.

Table S5 The stability of MIPs

Table S6 The separating effect of OC-MISPE

Part 3. Cartesian coordinates of the computed structures…………………………………13

Part 1. Experimental section

1. Method of validation of the HPLC and UHPLC analyses

1.1 Apparatus and analytical conditions

1.1.1 Apparatus

HPLC experiments were implemented using a Waters 2535 Series High-performance Liquid Chromatographic instrument. UHPLC analysis was performed on a Waters ACQUITY UPLC H-class system equipped with a quaternary solvent manager, a sample manager-FTN, and a PDA detector (Waters Corp., Massachusetts, USA) connected to a reversed-phase column (Waters Acquity UPLC BEH Shield RP C18, 100 mm × 2.1 mm, i.d., 1.7 μm).

1.1.2 Analytical conditions and method of validation of the HPLC analysis

The analytical column used was a Shim-pack VP-ODS column (4.6 × 250 mm, 4.5 μm, Shimadzu, Tokyo, Japan). The mobile phase consisted of 0.1% formic acid (A)-acetonitrile (B) in a gradient elution in which (B)% was 70, 100, 100, 70, and 70 at 0, 15, 35, 50, and 60 min, respectively, at a flow rate of 1.0 mL min^{-1}. The detection wavelength was at 327 nm and the injection volume was 10 μL. The method of validation of the HPLC analysis was described in Table S1.

1.1.3 Analytical conditions and method of validation of the UHPLC analysis

A gradient program was used at a flow rate of 0.4 mL min^{-1} by combining solvent A (0.1% formic acid) and solvent B (acetonitrile) as follows: 75–85% B (10 min), 85-100% B (3 min), 100-75% B (2 min) and 75% B (3 min). The column temperature was maintained at 40 °C. The injection volume was 5 μL, and the detection wavelength was set at 327 nm. The method of validation of the UHPLC analysis was described in Table S1.

Table S1. Standard curves, LOD, LOQs, linear ranges and Rt of the HPLC and UHPLC analyses

Entry	standard curve	LOD (µg mL^{-1})	LOQ (µg mL^{-1})	Linear range (µg mL^{-1})	Retention time (min)	Precision (RSD%)
HPLC(OC)	y=7.9274x-27.431 (R^2=0.9995)	2.0	6.0	6.0-200	40.808	3.00
HPLC(GK)	y=8.4102x-18.473 (R^2=0.9996)	1.5	5.0	5.0-200	35.617	3.73
UHPLC(OC)	y=21.077x-15.706 (R^2=0.9998)	0.08	0.40	0.48-200	8.531	0.88

Part 2. Figures and Tables

Figure S1. FTIR spectra of OC, NIPs, leached MIPs, and unleached MIPs

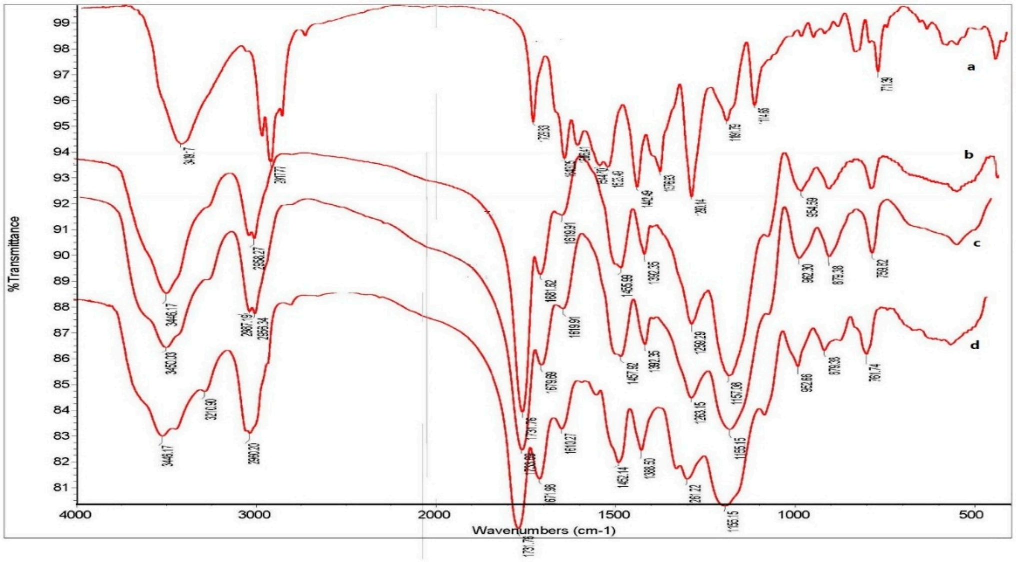

Fig. S1. FTIR spectra of OC (a), NIPs (b), leached MIPs, (c) and unleached MIPs (d)

Figure S2. ^{1}H NMR in DMSO-*d* of OC and OC + AM.

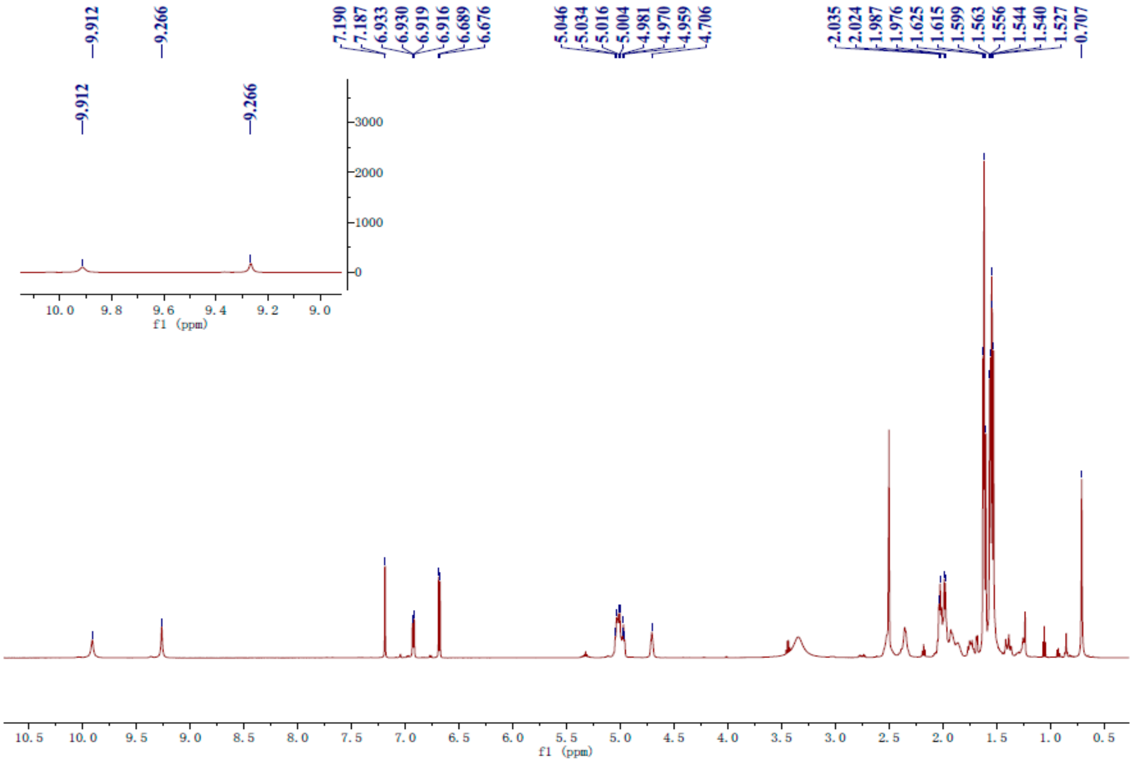

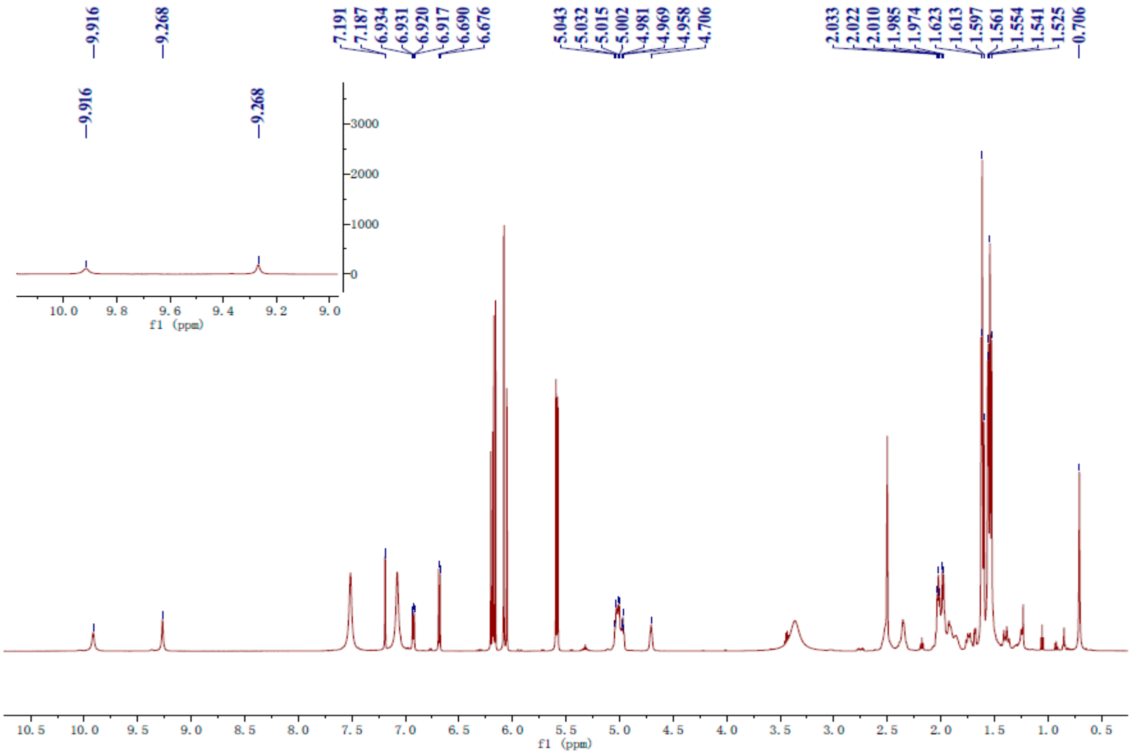

Fig. S2. ^{1}H NMR in DMSO-*d* of OC (top) and OC + AM (bottom).

Figure S3. ^{1}H NMR in DMSO-*d* of AM and OC + AM.

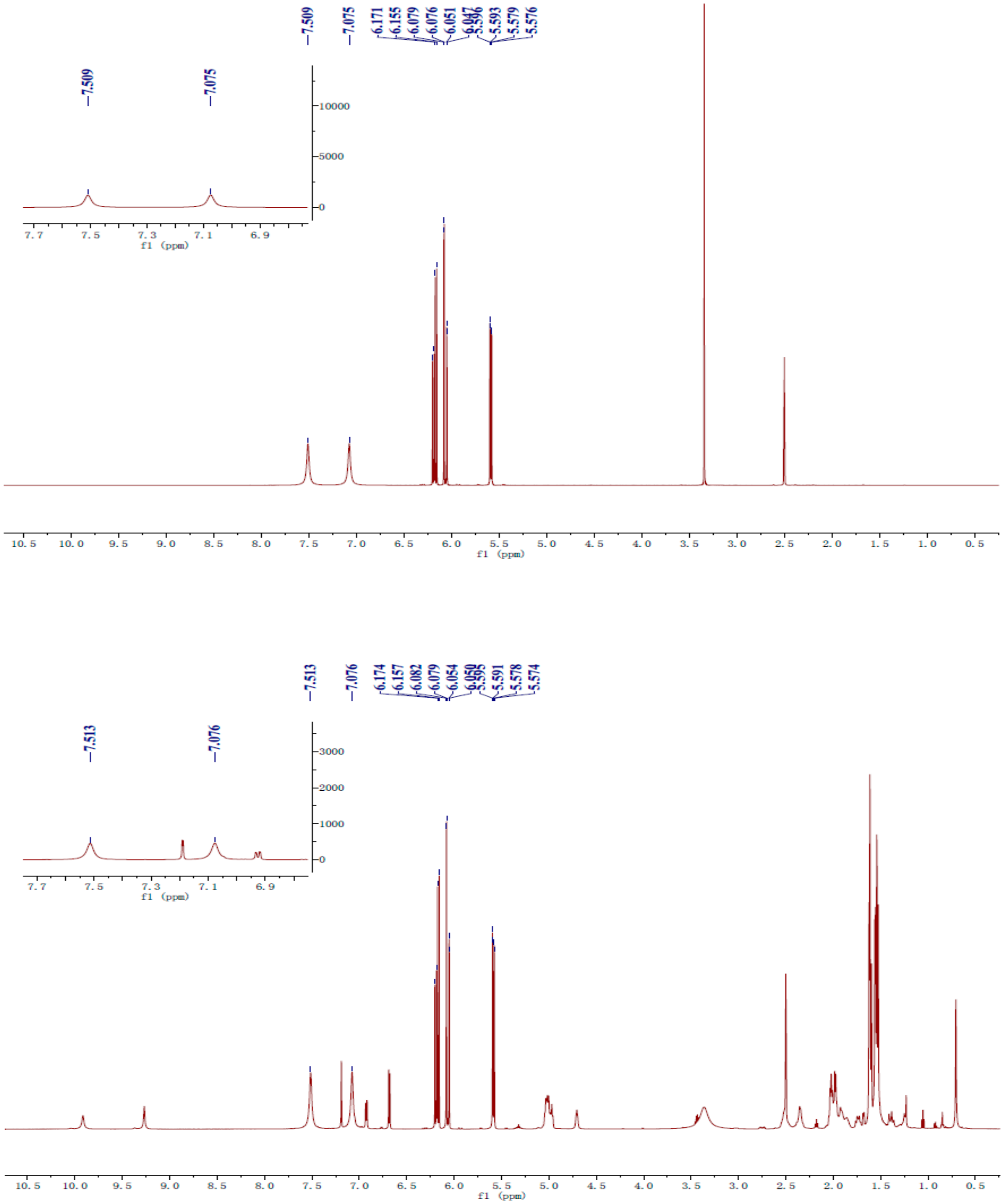

Fig. S3. ^{1}H NMR in DMSO-*d* of AM (top) and OC + AM (bottom).

Figure S4. ^{13}C NMR in DMSO-*d* of OC, AM and OC + AM.

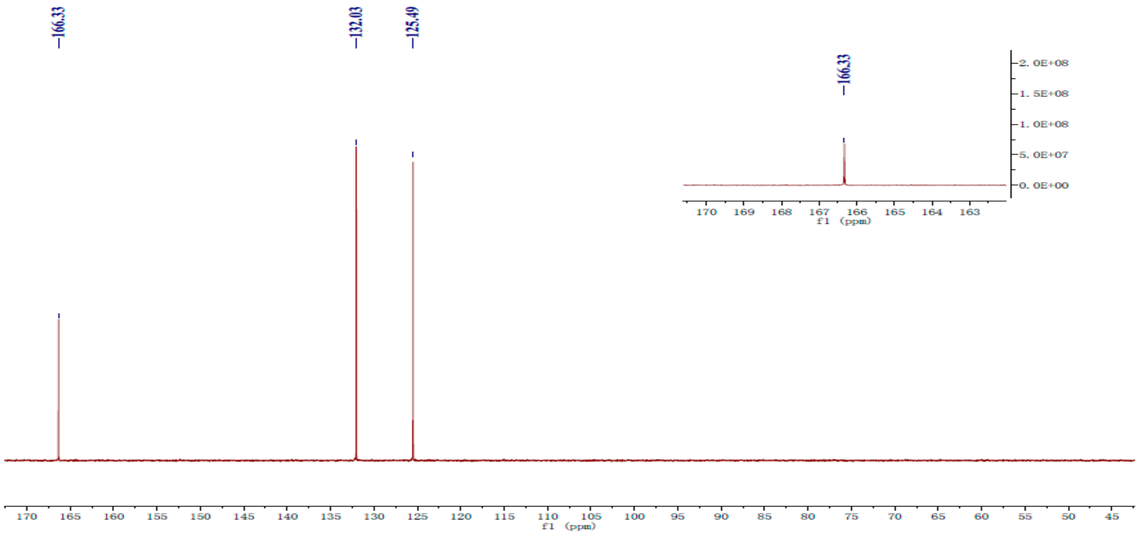

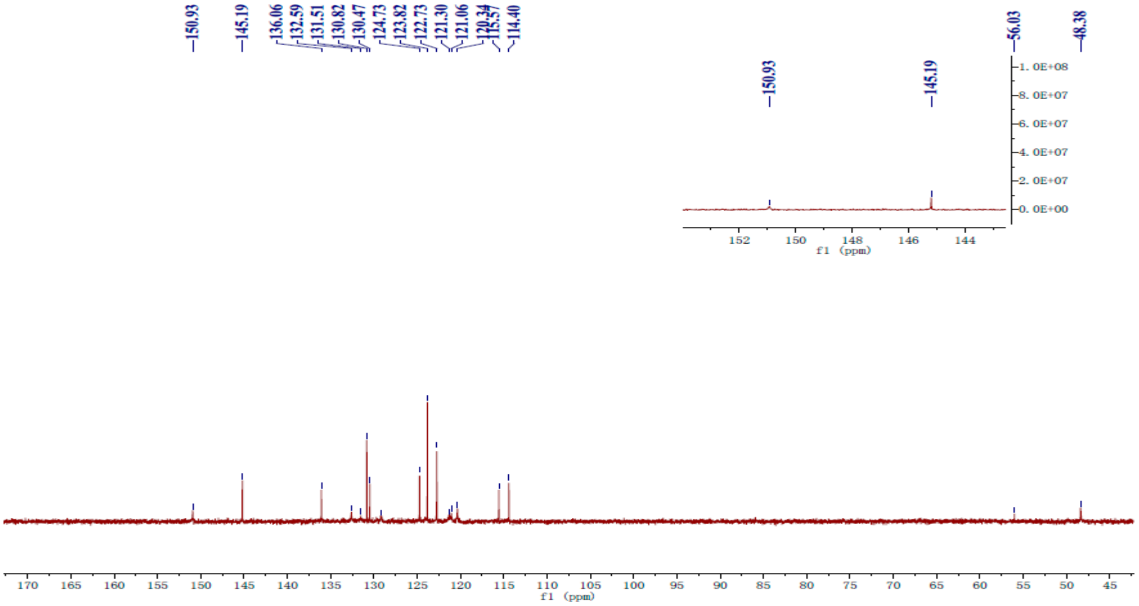

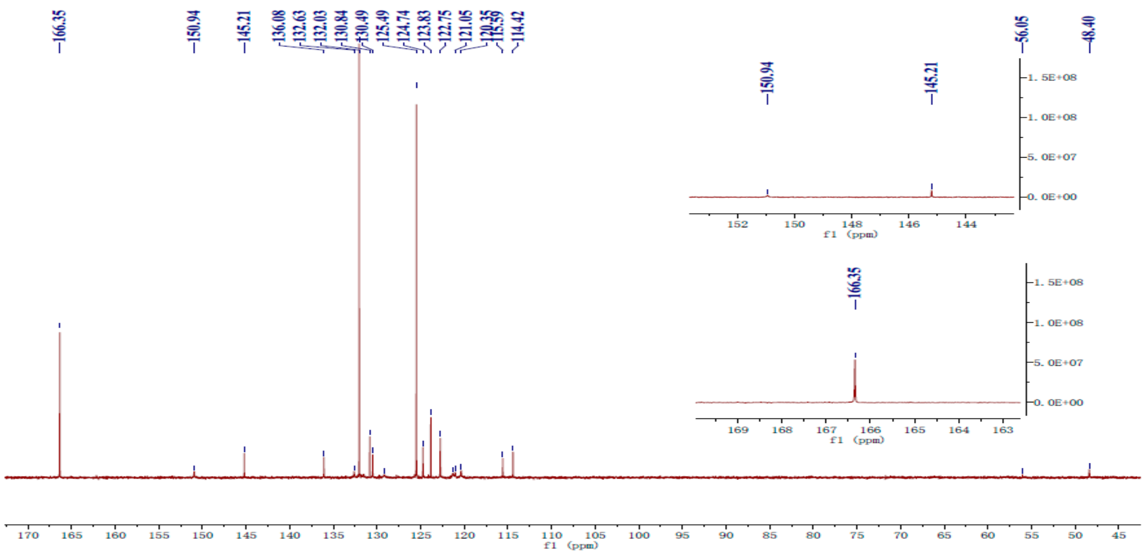

Fig. S4. ^{13}C NMR in DMSO-*d* of OC (top), AM (middle) and OC + AM (bottom).

Figure S5. SEM images of MIPs C and NIPs C.

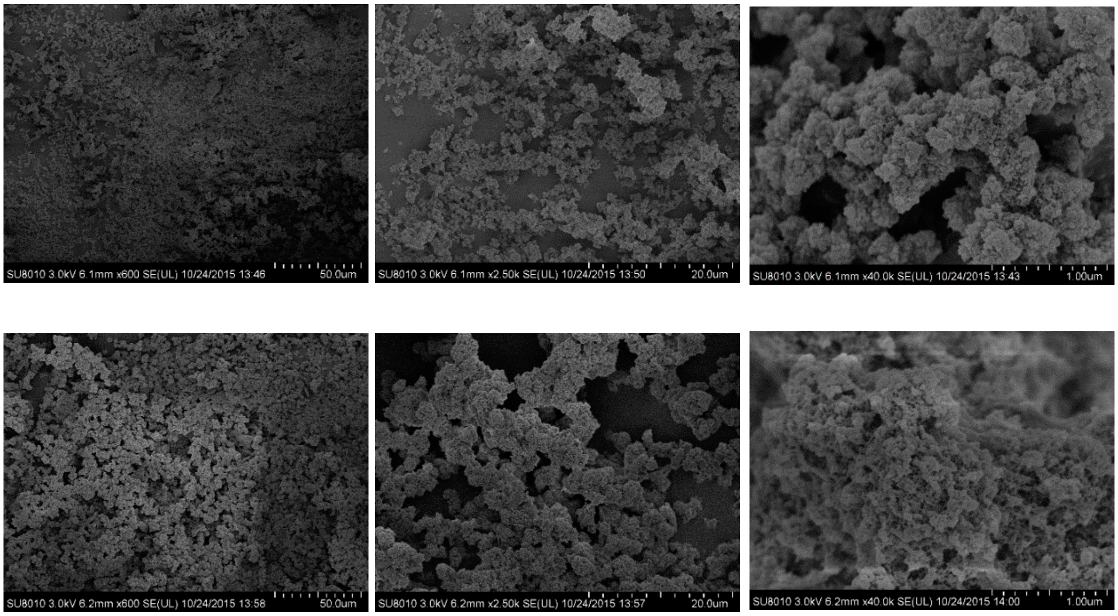

Fig. S5. SEM images of MIPs C (top) and NIPs C (bottom) at magnifications of 600×, 2500×, and 40,000× from left to right.

Figure S6. Evaluation of the adsorption properties

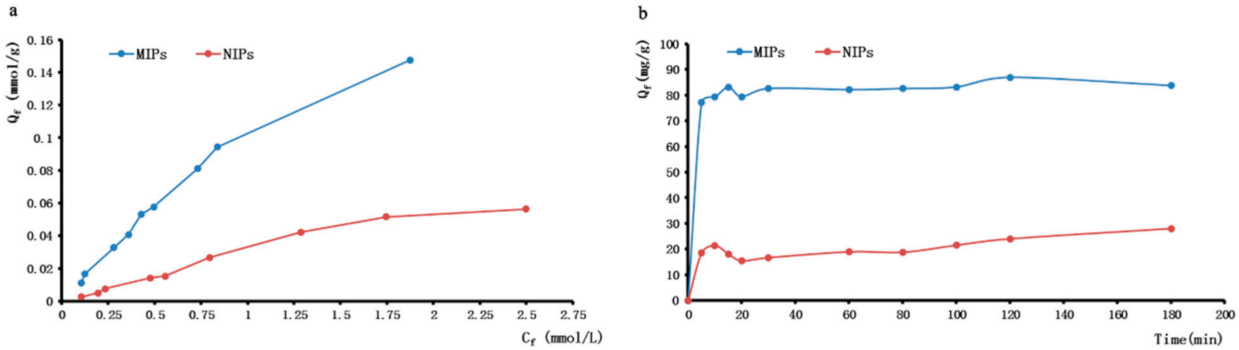

Fig. S6. Evaluation of the adsorption properties. a. The adsorption isotherms of OC on MIPs and NIPs. b. Dynamic adsorption isotherms of MIPs and NIPs for OC.

Figure S7. *Langmuir* and *Freundlich* isotherm for adsorption of OC on MIPs and NIPs.

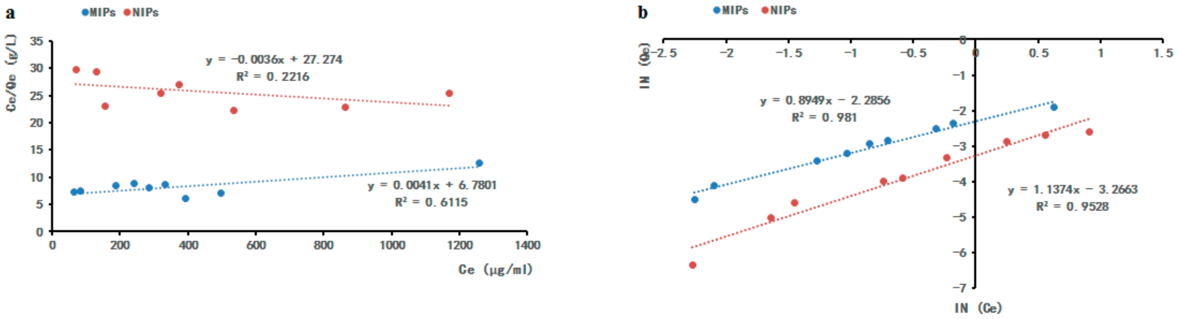

Fig. S7. *Langmuir* and *Freundlich* isotherm for adsorption of OC on MIPs and NIPs. a. *Langmuir* isotherm for adsorption of OC on MIPs and NIP. b. *Freundlich* isotherm for adsorption of OC on MIPs and NIPs

Figure S8. Chromatograms for elution solvent selection.

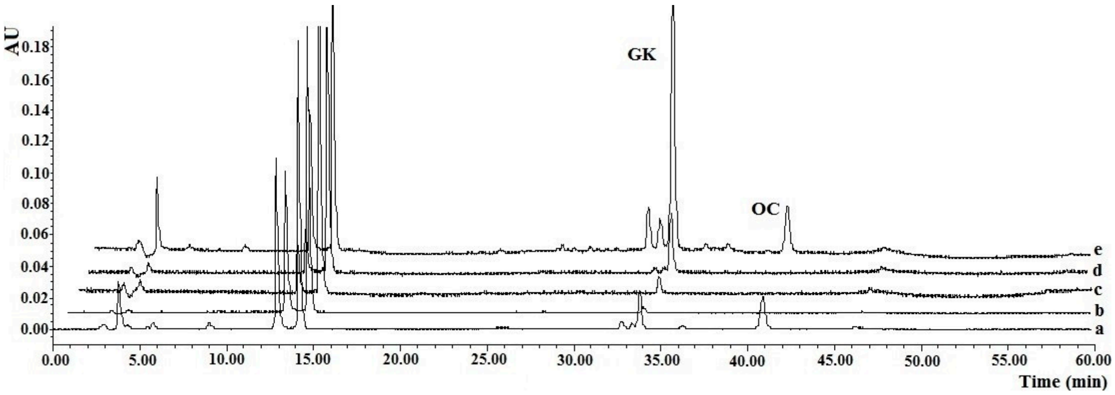

Fig. S8. Chromatograms for elution solvent selection. a Chromatograms of fruit extracts solutions. b Chromatogram of elution solutions from the OC-MISPE column of methanol-water (35:65, v/v). c Chromatogram of elution solutions from the OC-MISPE column of methanol-water (40:60, v/v). d Chromatogram of elution solutions from the OC-MISPE column of methanol-water (50:50, v/v). e Chromatogram of elution solutions from the OC-MISPE column of methanol-water (60:40, v/v).

Table S2. Binding rates of different loading solvents on the static adsorption

Table S2. Binding rates of different loading solvents on the static adsorption

solvent	binding rate	RSD[a] (%)
70% MeOH:H_2O	56.6%	0.8%
80% MeOH:H_2O	32.0%	2.2%
90% MeOH:H_2O	18.0%	1.8%
MeOH	7.9%	3.9 %
70% EtOH:H_2O	25.6%	1.5%
80% EtOH:H_2O	19.4%	4.1%
EtOH	20.3%	1.5%
MeCN	21.4%	0.9%

[a]. Tests were performed in triplicate (n = 3).

Table S3. Solution efficiency of different volumes of the loading solution of methanol-water (80:20, v/v)

Table S3. Solution efficiency of different volumes of the loading solution of methanol-water (80:20, v/v)

solvent volume (mL)	solution efficiency [a]		RSD [b] (%)	
	GK	OC	GK	OC
2	17.4%	16.1%	1.5%	1.8%
3	47.3%	36.4%	0.7%	0.9%
4	94.5%	84.8%	4.5%	4.0%
5	101.0%	99.7%	2.9%	4.9%

[a]. Solution efficiency = the amount of dissolved OC or GK / the amount of total OC or GK in fruit extracts. [b]. Tests were performed in triplicate (n = 3) with 1g of extracts.

Table S4. Binding rates of different loading amount of extracts

Table. S4. Binding rates of different loading amount of extracts

loading amount of extracts (g)	binding rates		RSD[a] (%)	
	GK	OC	GK	OC
1g	58.4%	76.9%	2.8%	2.5%
3g	33.8%	63.6%	4.6%	4.4%
5g	14.1%	57.8%	2.8%	3.5%

[a]. Tests were performed in triplicate (n = 3).

Table S5. The lifetime of MIPs

Table S5. The lifetime of MIPs

entry	reused times of MIPs	relative binding rate [a] (%)	RSD[c] (%)
1	5	88.05	4.10

2	10	77.17	4.73
3	20	70.28	4.59
4[b]	21	92.58	4.89

[a]. Relative binding rate (%) = xth binding rate / 1st binding rate ×100.
[b]. Acid activation after 20 times of MIPs.
[c]. Tests were performed in triplicate (n = 3).

Table S6. Purification of OC and GK in *G. yunnanensis* Hu fruit extracts

Table S6. Purification of OC and GK in *G. yunnanensis* Hu fruit extracts

entry[a]	loading amount of extracts (g)	purified amount of OC (mg)	recovery (%)	purified amount of GK (mg)	recovery (%)
1[b]	1	22.3	61.7	9.2	32.1
2[b]	3	52.0	48.0	21.6	25.1
3[c]	5	140.5	77.7	46.5	32.4
4[d]	12000	5000		3000	

[a]. Tests were repeated twice (n = 2).
[b]. 250 mg polymers for each test.
[c]. 2 g polymers for each test.
[d.] from the J. Agric. Food Chem. 2008, 56, 11144–11150.

Part 3. Cartesian coordinates of the computed structures:

Acrylamide (AM) in the DMSO
Energy = -247.118065481278 a.u.
O 0.521819367 1.325799372 0.001847414
N 1.626415015 -0.619098538 -0.001580827
C 0.462421251 0.094091494 0.000821088
C -0.811879963 -0.652398004 0.001871991
C -1.980987800 -0.010347528 -0.001497633
H -0.740611271 -1.741839130 0.005995244
H 1.652948939 -1.624979695 -0.003801869
H 2.521136118 -0.156021635 -0.001531100
H -2.939485980 -0.518203269 -0.000698151
H -2.071451529 1.071858363 -0.005558309

Oblongifolin C (OC) in the DMSO
Energy = -2122.94081692631 a.u.
C -4.023308460 -1.005639202 -1.689190683
C -5.356586326 -0.745635988 -1.992555367
C -6.222048904 -1.815883930 -2.216254848
C -5.758733035 -3.127549098 -2.126433552
C -4.427335759 -3.398512102 -1.791127870
C -3.562172890 -2.334758827 -1.587494521
C -6.665499539 -4.270067101 -2.366184458
C -7.569552593 -4.315859885 -3.492300739
C -8.555580534 -5.285287834 -3.535825481
C -9.684976486 -5.278506963 -4.528561169
C -9.803459895 -3.893009667 -5.159864798
C -8.495395000 -3.343167314 -5.684669336
C -7.414133906 -3.379746608 -4.601464006
C -8.094429729 -4.267401814 -6.900931708
C -9.346356143 -6.202020535 -5.710625669
C -8.063261909 -5.755109666 -6.436645715
O -8.590158253 -6.303943658 -2.716209041
C -9.151741630 -4.086763676 -8.004715717
C -6.726298174 -3.836831048 -7.489990130
C -7.824219757 -6.716270539 -7.623404923
C -7.066243007 -7.919765188 -7.172236028
C -5.969813855 -8.398665212 -7.775260632
C -5.243424416 -9.581526599 -7.202423844
C -5.370153238 -7.800122659 -9.003120696
O -6.456802110 -2.650243109 -4.693658681
O -10.866379587 -3.348284194 -5.296833178
C -10.991643201 -5.690805159 -3.831381695
C -11.252103666 -4.809329349 -2.654194163
C -11.376549689 -5.233663408 -1.388921400
C -11.606451149 -4.265954847 -0.275872194
C -11.292705916 -6.665755383 -0.983794724
C -4.029342283 -9.095211182 -6.386507831
C -4.466008896 -8.269382524 -5.221770661
C -3.879302137 -7.133922870 -4.822345569
C -4.411373397 -6.363506453 -3.658643078
C -2.689225741 -6.524808733 -5.481798109
C -5.475063936 -4.511932696 -6.904673129
C -4.257227792 -3.979092027 -7.587146487
C -3.313958259 -3.233503331 -6.995011805
C -2.114875098 -2.764297320 -7.753461666
C -3.361930934 -2.817203628 -5.564619255
O -2.243342524 -2.477483346 -1.291293855
O -3.217162508 0.064921236 -1.497567841
O -6.649398160 -5.202117970 -1.551872832
C -8.680943534 -1.862596855 -6.075824532
C -9.029907199 -1.024628238 -4.887976489
C -8.297552571 0.000413580 -4.429949041
C -8.744967798 0.788287832 -3.241871478
C -7.005156218 0.432740314 -5.033485507
H -5.707462991 0.282857711 -2.067234859
H -7.265840736 -1.615724702 -2.478091809
H -4.076407521 -4.427010900 -1.692538464
H -9.229529957 -7.246828302 -5.354422184
H -10.202795015 -6.235976826 -6.415048149
H -7.208151772 -5.867333850 -5.720942440
H -7.814464319 -6.240962800 -1.992978361
H -10.166865075 -4.294208945 -7.650457200
H -9.146689999 -3.068576847 -8.412008265
H -8.971138133 -4.758730092 -8.852746755
H -6.733147680 -4.013587846 -8.584807045
H -6.608707379 -2.738771674 -7.375624766
H -8.788783681 -7.024671311 -8.077788806
H -7.286963379 -6.189664159 -8.438983400
H -7.461221745 -8.393827492 -6.274437508
H -5.908418439 -10.191983819 -6.560001556
H -4.906151564 -10.264920198 -8.006547474
H -6.103865789 -7.714545083 -9.816996481
H -4.988865640 -6.785592929 -8.802820948
H -4.523159031 -8.381484041 -9.390705500
H -11.837173921 -5.620531760 -4.551222315
H -10.940707755 -6.763534363 -3.547160273
H -11.337740760 -3.750720449 -2.899078987
H -12.536859864 -4.488984942 0.266488469
H -11.670807465 -3.223250472 -0.615626745
H -10.789448953 -4.312277948 0.461337529
H -10.323267843 -7.106657675 -1.267826107
H -12.071484437 -7.271714716 -1.470316064
H -11.403642194 -6.813384660 0.098491109
H -3.444736427 -9.968982126 -6.031451366
H -3.347167289 -8.530424876 -7.054518333
H -5.344942925 -8.654699940 -4.706310796
H -5.333355190 -6.792922545 -3.244141000
H -4.641914002 -5.326208932 -3.948840922
H -3.676483492 -6.323320175 -2.840932138
H -2.026296134 -7.268618964 -5.940937332
H -3.007796865 -5.837233538 -6.285327963
H -2.078076932 -5.936506858 -4.783951394
H -5.422540575 -4.362150389 -5.809136631
H -5.514414761 -5.615101948 -7.041049016
H -4.180720058 -4.253618982 -8.637143328
H -1.186406962 -3.132409978 -7.291744570
H -2.051806531 -1.666673313 -7.764119727
H -2.107144835 -3.098222672 -8.798807473
H -4.325680869 -2.339722817 -5.315361003
H -2.572386948 -2.106735751 -5.295170375
H -3.262765379 -3.682234624 -4.891824568
H -1.997451866 -3.432701595 -1.131194023
H -2.284208450 -0.210508842 -1.263510534
H -7.754261988 -1.497743871 -6.570884298
H -9.485784848 -1.768853553 -6.836076502
H -9.964850554 -1.302174420 -4.403415364
H -9.671522666 0.404989534 -2.793783744
H -8.924526498 1.840508435 -3.505743113

H -7.981655470 0.786218231 -2.449035906
H -7.105917064 0.663813499 -6.102651502
H -6.577998059 1.319081149 -4.549260935
H -6.251945040 -0.373469106 -4.950860243

OC-AM complex in the DMSO

Energy = -2370.06266710 a.u. #Cluster: 1

O	3.31328274	-3.15294742	-2.50336738
N	3.31907402	-1.62903197	-4.14384646
C	2.65737647	-2.50608560	-3.33345416
C	1.20093069	-2.65241476	-3.49837087
C	0.52254398	-3.57898480	-2.81940938
H	0.71826732	-1.97324222	-4.20426850
H	2.84760754	-1.01719951	-4.79295024
H	4.30776582	-1.46678057	-4.02347271
H	-0.54809186	-3.72308780	-2.91440072
H	0.98307844	-4.26817329	-2.11641177
C	6.35982492	0.23691379	-1.72011127
C	6.18163387	1.40942331	-0.99275474
C	5.10049183	1.51648416	-0.11591226
C	4.21967440	0.45003880	0.02990328
C	4.39659773	-0.73819706	-0.68360393
C	5.46670173	-0.84500512	-1.56200935
C	3.06212585	0.51966312	0.94893177
C	1.91067553	1.34657263	0.67122890
C	0.79454191	1.25927974	1.48080642
C	-0.45880819	2.06059248	1.25622768
C	-0.05036975	3.37266936	0.58725959
C	0.73653266	3.18943218	-0.69704731
C	1.93456852	2.26227035	-0.46928156
C	-0.26018327	2.54193683	-1.73403813
C	-1.35469898	1.32212203	0.24906610
C	-0.73919577	1.18206581	-1.15326955
O	0.71641869	0.45324349	2.50967531
C	-1.45279482	3.49769699	-1.91513889
C	0.39266625	2.36700914	-3.12670167
C	-1.79331245	0.48923099	-2.04778924
C	-1.91560658	-0.93326998	-1.61354936
C	-2.94036634	-1.45425604	-0.92640142
C	-2.87910189	-2.87631623	-0.44586713
C	-4.18393527	-0.71853603	-0.56385250
O	2.87353221	2.31278131	-1.22551450
O	-0.36419084	4.44102372	1.03724988
C	-1.19907546	2.29730162	2.58640458
C	-2.53903403	2.91774890	2.36530688
C	-3.70599298	2.26917259	2.48741350
C	-5.00194906	2.96133048	2.22670835
C	-3.81611426	0.83209493	2.87251263
C	-2.22791156	-2.93677206	0.95214490
C	-0.78273367	-2.56560151	0.88280786
C	0.21367894	-3.43574531	0.66964872
C	1.63350938	-2.98307525	0.59375763
C	-0.00432719	-4.90074113	0.49174295
C	1.15720042	1.05841670	-3.38728326
C	1.74947612	1.11127204	-4.75972551
C	3.00139124	1.51395125	-5.02380057
C	3.52910108	1.53653099	-6.41987415
C	3.95185580	1.96858277	-3.97261290
O	5.70046573	-1.96828003	-2.28019593
O	7.37116549	0.05953619	-2.60151043
O	3.07862103	-0.20297260	1.95217122
C	1.30468213	4.55122608	-1.14519631
C	2.27843237	5.07927945	-0.14080446
C	3.58981046	5.26057939	-0.35073736
C	4.47468075	5.76399486	0.74365296
C	4.28790678	4.96834026	-1.63373458
H	6.87182945	2.24338970	-1.11925998
H	4.94013083	2.44519942	0.43560538
H	3.70270540	-1.57282608	-0.56504900
H	-1.61103482	0.31499967	0.64327949
H	-2.33172627	1.84746749	0.17735455
H	0.15059153	0.50545093	-1.08445058
H	1.62444496	-0.07934709	2.65649327
H	-1.96649065	3.71981827	-0.97400632
H	-1.14228655	4.45508515	-2.35114448
H	-2.20567628	3.07404757	-2.59181679
H	-0.39237555	2.46223949	-3.90556947
H	1.08244182	3.21567658	-3.31601739
H	-2.75905398	1.02805148	-2.00022277
H	-1.49995839	0.54141035	-3.11656361
H	-1.04490928	-1.54585018	-1.85514110
H	-3.89112376	-3.32442440	-0.40432109
H	-2.30562377	-3.51275587	-1.14891624
H	-4.06180196	0.37305272	-0.57990422
H	-4.99919812	-0.96029856	-1.26326622
H	-4.54098206	-0.98388152	0.44209282
H	-1.29202760	1.33746170	3.14131161
H	-0.58281709	2.95398444	3.24030640
H	-2.50478884	3.96679870	2.07435515
H	-5.54568061	2.48495098	1.39631606
H	-4.88167531	4.02049485	1.96249345
H	-5.66473182	2.92051164	3.10382363
H	-3.29902322	0.18045797	2.15181145
H	-4.85583866	0.48194737	2.92424923
H	-3.36700925	0.63882623	3.85906668
H	-2.77021859	-2.26049822	1.64374296
H	-2.36195977	-3.95426451	1.37292181
H	-0.58557441	-1.50118090	0.99651358
H	1.75748260	-1.92368314	0.85261992
H	2.03570087	-3.12248327	-0.42451272
H	2.27860163	-3.55949972	1.27311711
H	-0.38023123	-5.36414205	1.41648101
H	-0.75120533	-5.10834760	-0.28888552
H	0.91110878	-5.43897788	0.21183102
H	1.94232785	0.89886651	-2.62208391
H	0.47939665	0.18475429	-3.30505454
H	1.07148467	0.81197828	-5.55569430
H	4.43248825	0.91553108	-6.51880016
H	3.81375486	2.55594704	-6.72069095
H	2.80635243	1.17379671	-7.16218465
H	3.57213417	2.87046621	-3.46119157
H	4.94907117	2.20516694	-4.35990016
H	4.07823418	1.22054361	-3.17199561
H	4.87163223	-2.57020001	-2.28737790
H	7.91812939	0.88936344	-2.71577401
H	1.77845708	4.43992082	-2.14520925
H	0.47727747	5.27934638	-1.28293670
H	1.83479333	5.31900795	0.82496903
H	3.94439170	5.91658393	1.69283573
H	4.94009327	6.72310813	0.47534078
H	5.29464849	5.05708166	0.94373541
H	3.69763591	5.26005629	-2.51243741
H	5.26044554	5.46911073	-1.71621460

H	4.47635755	3.88165593	-1.72526042

Energy = -2370.05841892 a.u. #Cluster: 2

O	7.28733257	4.44008637	0.75450532
N	5.24454221	3.56847813	1.04867026
C	6.28624168	3.93645570	0.24257320
C	6.15716700	3.67213922	-1.20446670
C	7.14212402	3.97066662	-2.05142775
H	5.21132116	3.22787019	-1.53214538
H	4.38413655	3.21809665	0.65916449
H	5.24724411	3.77416681	2.03371618
H	7.08408291	3.79449034	-3.11983453
H	8.08398943	4.41254652	-1.74473811
C	5.19999955	-0.74567056	-2.48449927
C	5.82300904	0.08005356	-1.55926331
C	5.11906543	0.46898360	-0.41631549
C	3.80542521	0.05704521	-0.23509340
C	3.16782420	-0.78060732	-1.15757624
C	3.87392667	-1.18889301	-2.28136688
C	3.03586475	0.46274729	0.96492129
C	1.95846187	1.42612119	0.84345822
C	0.91255114	1.39652561	1.74041428
C	-0.36442442	2.16190286	1.53227040
C	-0.01929798	3.45746071	0.79666288
C	0.73613197	3.24970516	-0.50813268
C	1.99347969	2.42384948	-0.22302938
C	-0.26925133	2.51179911	-1.47334912
C	-1.25308101	1.33785905	0.58214233
C	-0.66181766	1.15488875	-0.82598622
O	0.90673446	0.65107435	2.82105314
C	-1.51531128	3.40388679	-1.63829446
C	0.31467991	2.31555138	-2.89102029
C	-1.68880844	0.36925552	-1.67182262
C	-1.87859069	-0.98573145	-1.07485159
C	-2.94784571	-1.38302565	-0.37248697
C	-2.97873898	-2.75089838	0.24757597
C	-4.16310855	-0.55727978	-0.12597704
O	3.01727999	2.64915452	-0.82772636
O	-0.34699896	4.53412674	1.21333455
C	-1.07685397	2.43561998	2.86853661
C	-2.45209329	2.97524183	2.65174422
C	-3.58043453	2.28079969	2.85640001
C	-4.91725596	2.88772890	2.59074626
C	-3.60437781	0.87188780	3.34605307
C	-2.37157415	-2.72013970	1.66530858
C	-0.90778788	-2.42126271	1.63783370
C	0.04026461	-3.28628143	1.25225261
C	1.48481013	-2.90630591	1.25251604
C	-0.23543094	-4.67432285	0.78348173
C	1.29090915	1.14669719	-3.07757479
C	1.86839718	1.19435304	-4.45640344
C	3.03341751	1.78314382	-4.75951165
C	3.57182293	1.77528811	-6.15189171
C	3.88389421	2.47547294	-3.75051942
O	3.24560821	-2.03143826	-3.13635938
O	5.79762134	-1.20267466	-3.61206958
O	3.25563266	-0.09372808	2.03749534
C	1.19597737	4.60936137	-1.06623250
C	2.16049492	5.31123255	-0.16594449
C	3.20014971	6.03604693	-0.60246185
C	4.09687316	6.75197562	0.35306884
C	3.55312447	6.18786170	-2.04370044
H	6.84825182	0.41612428	-1.71839764
H	5.61100077	1.09770145	0.32806933
H	2.13924811	-1.11131146	-1.01490173
H	-1.45840143	0.34242941	1.03274378
H	-2.25422477	1.81423176	0.50965657
H	0.26051734	0.52588313	-0.75181840
H	1.81273734	0.14887988	2.95644878
H	-1.99666186	3.64777369	-0.68583014
H	-1.27512260	4.35267964	-2.13359289
H	-2.27785861	2.91487636	-2.25818070
H	-0.52338619	2.18230959	-3.60761652
H	0.81127584	3.25194558	-3.21764829
H	-2.64180887	0.92876537	-1.74332712
H	-1.33515677	0.27560814	-2.72005937
H	-1.02870310	-1.65591695	-1.21239688
H	-4.01587475	-3.13738271	0.29907865
H	-2.42411840	-3.48140254	-0.37570661
H	-4.04185748	0.49537763	-0.41445171
H	-5.02614202	-0.94707443	-0.68722147
H	-4.44691455	-0.56330534	0.93720041
H	-1.10496582	1.50728908	3.48080849
H	-0.47766324	3.15826169	3.46706188
H	-2.47886021	4.00236207	2.29033966
H	-5.44390157	2.34788788	1.78833376
H	-4.86109201	3.94029715	2.28219073
H	-5.56462165	2.84544658	3.47911991
H	-2.99345720	0.21112919	2.71261305
H	-4.61580056	0.44399830	3.36839237
H	-3.20444190	0.79376415	4.36928875
H	-2.91201179	-1.97302887	2.28100321
H	-2.56078878	-3.69573100	2.16177776
H	-0.64764834	-1.41313068	1.95561952
H	1.65280553	-1.85330255	1.50817722
H	1.94462621	-3.08709205	0.26937043
H	2.05246166	-3.50574091	1.98104197
H	-1.16581064	-5.08643460	1.19839295
H	-0.33743642	-4.70091646	-0.31325063
H	0.57095824	-5.37251451	1.04672162
H	2.09826480	1.17923983	-2.31988589
H	0.77754665	0.17607793	-2.92092922
H	1.26723239	0.70353034	-5.21647910
H	4.55875218	1.28941813	-6.18927428
H	3.70377240	2.79674268	-6.53723302
H	2.92579951	1.24214595	-6.86102514
H	3.29550650	3.18288839	-3.14244395
H	4.71643134	3.03065091	-4.19701987
H	4.31281642	1.75127061	-3.03678430
H	3.75756459	-2.13101605	-3.98946950
H	6.66481193	-0.73905869	-3.79320238
H	1.64556213	4.44882128	-2.07203521
H	0.31535615	5.26863012	-1.23220728
H	1.94889730	5.22434383	0.89783713
H	3.84736288	6.55758131	1.40509721
H	4.05059842	7.84079970	0.20383563
H	5.14679194	6.45459929	0.21306340
H	2.69309313	6.50874827	-2.64837253
H	4.35380478	6.91953157	-2.21347493
H	3.90373340	5.23044569	-2.46230244

Energy = -2370.05569446 a.u. #Cluster: 3

O	2.12715531	-0.51913071	4.32482056
N	3.98950290	-1.30034589	3.35371148

C	2.84890112	-1.49177298	4.07354222
C	2.53834859	-2.84524379	4.57088389
C	1.27375572	-3.21024319	4.78686983
H	3.38711746	-3.50858380	4.74213341
H	4.58641081	-2.05494182	3.06068346
H	4.21249057	-0.38118135	2.99557756
H	0.98893521	-4.19130807	5.14946387
H	0.42323368	-2.55802862	4.60802831
C	4.81531719	-0.99506328	-1.63873145
C	5.59923119	-0.07911585	-0.94999934
C	5.01527272	0.68689215	0.06157509
C	3.66328387	0.54110791	0.35422460
C	2.86357193	-0.37514703	-0.33871679
C	3.44356942	-1.14964493	-1.33407024
C	3.05429303	1.37053032	1.41875197
C	1.79486796	2.05027776	1.12306470
C	0.69201868	1.91541737	1.91269106
C	-0.63778367	2.54307378	1.56546165
C	-0.37133305	3.80884191	0.75357106
C	0.49548412	3.59265562	-0.47667734
C	1.80157912	2.91161519	-0.06081581
C	-0.34628680	2.68042552	-1.45157152
C	-1.38642522	1.58024083	0.62782627
C	-0.67757157	1.35040369	-0.71782549
O	0.60992460	1.19158276	3.00442767
C	-1.64442800	3.43043296	-1.80463707
C	0.39405562	2.42043049	-2.78594316
C	-1.56675133	0.41075101	-1.56451470
C	-1.61834350	-0.92649810	-0.90258217
C	-2.63747185	-1.39085465	-0.16758826
C	-2.51455549	-2.70065448	0.55805487
C	-3.93450841	-0.68597753	0.03469846
O	2.82485451	3.11502676	-0.66992173
O	-0.84585376	4.87010870	1.05673365
C	-1.45487794	2.84441334	2.83490666
C	-2.87025700	3.18686067	2.50425998
C	-3.92051134	2.39174160	2.75252217
C	-5.30363656	2.80061674	2.36994270
C	-3.81071102	1.05917784	3.41459935
C	-1.70497624	-2.51831739	1.86029047
C	-0.24418625	-2.40479527	1.56603324
C	0.57980283	-3.45359651	1.43512816
C	2.02453758	-3.27184730	1.10800643
C	0.13858074	-4.86971598	1.59614779
C	1.41853339	1.27690577	-2.79729451
C	2.08984429	1.21320914	-4.13150923
C	3.32004220	1.68327158	-4.37915665
C	3.94421693	1.55238692	-5.72955808
C	4.15966688	2.36215841	-3.35235366
O	2.64581982	-2.04069760	-1.97045119
O	5.28610478	-1.80132862	-2.61919438
O	3.57828800	1.45524614	2.51452388
C	0.85971712	4.95630923	-1.09518578
C	1.69204590	5.78062086	-0.16642162
C	2.91777978	6.24494538	-0.44673552
C	3.67572852	7.06189573	0.54769527
C	3.62647526	5.99023916	-1.73333404
H	6.65485865	0.03792271	-1.19423844
H	5.62407600	1.40699907	0.61336436
H	1.80177281	-0.49118579	-0.11570657
H	-1.53583843	0.60595613	1.14204984
H	-2.41524762	1.95773293	0.45108040
H	0.28759196	0.81682206	-0.52450000
H	1.44343412	0.66204023	3.30285437
H	-2.22680584	3.71081498	-0.92101888
H	-1.44386347	4.34937456	-2.36854055
H	-2.30246595	2.81802179	-2.43373702
H	-0.35628644	2.21737057	-3.57816465
H	0.90254124	3.35076944	-3.11136647
H	-2.57503246	0.84777698	-1.69988954
H	-1.16041977	0.31275871	-2.59235960
H	-0.70581029	-1.51477548	-1.01361104
H	-3.51045890	-3.12050987	0.79805746
H	-2.02491672	-3.46252341	-0.08103236
H	-4.11634249	-0.48337479	1.10119384
H	-3.99454651	0.27898541	-0.48683218
H	-4.77961249	-1.29405475	-0.32093343
H	-1.40877137	1.97825923	3.53135525
H	-0.98212626	3.68594331	3.38878978
H	-2.99625081	4.15293233	2.01741028
H	-5.71771896	2.12649292	1.60415050
H	-5.35653583	3.81789050	1.95948177
H	-5.98980531	2.76265440	3.22855600
H	-3.00681695	0.44647105	2.98018157
H	-4.73473895	0.47001649	3.34180595
H	-3.58744375	1.16482829	4.48815074
H	-2.06436748	-1.62072109	2.40242008
H	-1.90737971	-3.36958425	2.54151222
H	0.11269074	-1.38498178	1.44269850
H	2.39667367	-2.27118184	1.36100731
H	2.19901947	-3.41256588	0.02796338
H	2.66210117	-3.99607621	1.63264211
H	0.06916647	-5.13832396	2.66152813
H	-0.85217946	-5.05109003	1.15606782
H	0.83009032	-5.58358044	1.13007379
H	2.16616114	1.40889211	-1.99036815
H	0.93187620	0.30152745	-2.58694904
H	1.50022709	0.73652884	-4.90998372
H	4.87934978	0.97457529	-5.67568292
H	4.20034789	2.53501556	-6.15131419
H	3.29729233	1.04580810	-6.45696918
H	3.60715717	3.18117965	-2.86041831
H	5.08532995	2.78305170	-3.75922468
H	4.44318269	1.66771608	-2.54427102
H	3.11366313	-2.45217141	-2.75199742
H	6.22298261	-1.57114118	-2.88400123
H	1.38472143	4.78825530	-2.06098936
H	-0.06672470	5.51549562	-1.34955686
H	1.21926262	5.99899394	0.78951563
H	3.11574229	7.24448218	1.47439300
H	3.95022701	8.04410767	0.13664451
H	4.61447400	6.56260439	0.83277418
H	3.05448138	6.35847379	-2.59665581
H	4.61707382	6.45856074	-1.78052200
H	3.77522348	4.90598622	-1.88572640

Energy = -2370.05003075 a.u. #Cluster: 4

O	-2.43279420	8.69987674	1.50152544
N	-0.89342013	7.21709283	0.82474599
C	-1.85542779	7.62951737	1.70087599
C	-2.13793257	6.76324707	2.86539580
C	-3.33241181	6.77254619	3.45583397
H	-1.31575123	6.12313140	3.19873943
H	-0.43102061	6.32483841	0.93504807

H	-0.67061390	7.75012815	0.00063356
H	-3.57855704	6.15527757	4.31254374
H	-4.15935801	7.39612512	3.13201867
C	5.99867916	0.30085280	-1.95402730
C	6.04704544	1.29361114	-0.98283446
C	5.05699385	1.32637481	0.00034679
C	4.04506577	0.37003617	0.00031978
C	3.98660380	-0.63385127	-0.96981895
C	4.96783559	-0.66162311	-1.95333313
C	2.98883048	0.39927175	1.03902428
C	1.89194997	1.33681043	0.96670675
C	0.84665686	1.24772028	1.86481259
C	-0.36579545	2.13675291	1.82329571
C	0.02083112	3.45547880	1.15889574
C	0.69310054	3.30231807	-0.19049721
C	1.90602228	2.37692997	-0.06206891
C	-0.38228985	2.67927106	-1.16442655
C	-1.40909750	1.50593915	0.88520355
C	-0.91018423	1.34700313	-0.55917085
O	0.79881697	0.35119102	2.81926329
C	-1.53331360	3.69247892	-1.30283498
C	0.19922178	2.44783611	-2.58057263
C	-2.06689328	0.74626437	-1.39223388
C	-2.34298198	-0.63918317	-0.91225681
C	-3.35935624	-1.00279194	-0.11857886
C	-3.45058811	-2.41516763	0.38696691
C	-4.44456600	-0.09706206	0.35109364
O	2.85165761	2.53192481	-0.79503572
O	-0.24003721	4.51264098	1.66852158
C	-0.93755559	2.35287709	3.23679071
C	-2.28772750	2.98791498	3.18454365
C	-3.43646858	2.35899023	3.46892984
C	-4.74592087	3.06798857	3.36802681
C	-3.51319348	0.93188008	3.89607146
C	-2.56521240	-2.60417479	1.63772193
C	-1.12666162	-2.33165304	1.34008481
C	-0.32887544	-3.12671203	0.61424543
C	1.08086528	-2.73662390	0.30914157
C	-0.75786602	-4.42642539	0.02339727
C	0.95160982	1.12739785	-2.82168082
C	1.47680444	1.09934852	-4.22207837
C	2.64965110	1.62823417	-4.59849124
C	3.12213666	1.54272637	-6.01268816
C	3.56766531	2.33233236	-3.66227366
O	4.88749296	-1.65189541	-2.87839430
O	6.91781196	0.17046884	-2.94297383
O	3.03961539	-0.43260627	1.94997009
C	1.22318167	4.66911074	-0.67340243
C	2.22033999	5.24346668	0.28084741
C	3.50075940	5.51216630	-0.01208667
C	4.42018645	6.06707061	1.02711734
C	4.12545770	5.27711908	-1.34386624
H	6.83937754	2.04206474	-0.99526160
H	5.06893710	2.11622679	0.75396600
H	3.19066439	-1.37747372	-0.97356123
H	-1.72231962	0.51702884	1.28472113
H	-2.33857417	2.11421648	0.90171480
H	-0.06911737	0.60831447	-0.56009990
H	1.66664276	-0.25603176	2.82874317
H	-1.96867963	3.96815295	-0.33674610
H	-1.20778797	4.61585393	-1.79723957
H	-2.35118690	3.29171011	-1.91539389
H	-0.62646723	2.50886549	-3.31979800
H	0.87296950	3.28956312	-2.84069586
H	-2.96482508	1.39217804	-1.32913592
H	-1.80068359	0.73270876	-2.46964113
H	-1.59221013	-1.37173321	-1.21515176
H	-4.49619595	-2.68484994	0.63317598
H	-3.13884525	-3.13355218	-0.39804504
H	-4.32368476	0.94136720	0.01395133
H	-5.42943835	-0.43674982	-0.00311405
H	-4.49359508	-0.06997175	1.45042246
H	-0.97190607	1.38671135	3.78555528
H	-0.24233886	2.99561903	3.82271261
H	-2.27771192	4.03373693	2.87607525
H	-5.38300804	2.61211061	2.59429038
H	-4.64314567	4.13093174	3.11106066
H	-5.30730850	3.01576786	4.31228368
H	-2.96040546	0.26908198	3.21360925
H	-4.54363172	0.55427701	3.94148720
H	-3.07906804	0.79000494	4.89830283
H	-2.92903632	-1.93912661	2.44663166
H	-2.69817214	-3.63637330	2.02450140
H	-0.75691644	-1.38942170	1.74262123
H	1.37235088	-1.78926991	0.77999990
H	1.22471891	-2.62602416	-0.77698607
H	1.79508966	-3.49995456	0.65169491
H	-1.57476179	-4.89878260	0.58652523
H	-1.12290661	-4.28500229	-1.00624076
H	0.06271958	-5.15525921	-0.02495667
H	1.78250855	1.00160662	-2.09924954
H	0.28105242	0.25948242	-2.65720929
H	0.82507859	0.60726448	-4.93872455
H	4.07203439	0.99012330	-6.07650980
H	3.30687765	2.54139452	-6.43455069
H	2.41001293	1.03683139	-6.67686769
H	3.07722651	3.19775296	-3.18839876
H	4.48618861	2.69068007	-4.13903297
H	3.86846820	1.67817716	-2.82465985
H	5.54971673	-1.51379882	-3.61390319
H	7.53869977	0.95337687	-2.98355990
H	1.66910881	4.54381062	-1.68604527
H	0.38330487	5.38029404	-0.81237967
H	1.83246493	5.44267086	1.27872574
H	3.93165283	6.22397799	1.99775720
H	4.84420012	7.03271185	0.71709916
H	5.26828708	5.38664831	1.20115614
H	3.51491649	5.67189132	-2.16721976
H	5.12301246	5.72326447	-1.43683350
H	4.24388976	4.19190771	-1.52364916

Energy = -2370.04927123 a.u. #Cluster: 5

O	4.32771805	-3.35106866	-4.60243917
N	4.16219000	-1.28829331	-5.47291734
C	4.70892738	-2.54086395	-5.45753981
C	5.68190254	-2.89563703	-6.50688357
C	6.48448102	-3.95116302	-6.36265172
H	5.70701883	-2.24995996	-7.38712671
H	4.51115237	-0.56000565	-6.07411190
H	3.56655408	-0.97811571	-4.71736055
H	7.21886450	-4.24696981	-7.10395229
H	6.47368613	-4.59983032	-5.49158704
C	5.13912844	-0.84293014	-2.09345907
C	5.73977498	0.17653502	-1.36714595

C	5.07400098	0.70978143	-0.25967099
C	3.82491756	0.21798827	0.09245052
C	3.20465889	-0.80330728	-0.63505670
C	3.86811769	-1.34341878	-1.73069838
C	3.08044800	0.75289585	1.25636690
C	1.94191889	1.61920125	1.00477655
C	0.84550861	1.57436470	1.82642112
C	-0.47348739	2.18829641	1.45382926
C	-0.17154019	3.47940349	0.69090164
C	0.72173281	3.29208420	-0.52455369
C	1.99315784	2.52689249	-0.14332799
C	-0.13738948	2.45732201	-1.55290330
C	-1.18250017	1.24757464	0.46134091
C	-0.47584585	1.08999603	-0.89672081
O	0.82538024	0.93749194	2.98124282
C	-1.42899533	3.24409729	-1.84786242
C	0.58491167	2.27665676	-2.90865853
C	-1.38531054	0.21782714	-1.79138065
C	-1.48748294	-1.14923897	-1.20066732
C	-2.58031944	-1.64778241	-0.60691938
C	-2.54528515	-3.01014863	0.02070870
C	-3.87477190	-0.91492296	-0.49895950
O	3.02030039	2.70562725	-0.75062599
O	-0.64447438	4.53272293	1.02115879
C	-1.34412981	2.44255355	2.69736633
C	-2.73472858	2.83478794	2.32025651
C	-3.80572884	2.03627440	2.43284854
C	-5.15895453	2.49883348	2.00723744
C	-3.74760846	0.64676915	2.97263734
C	-2.31149549	-2.89975417	1.54115417
C	-0.94349713	-2.39003786	1.86007682
C	0.18651521	-3.09852148	1.72973240
C	1.51351845	-2.51392284	2.08909359
C	0.24298793	-4.50013741	1.22539837
C	1.59183247	1.12062750	-3.03148619
C	2.23776272	1.19859219	-4.37898741
C	3.37411545	1.86609346	-4.62605648
C	3.93820721	1.96569052	-6.00488510
C	4.14577683	2.57618253	-3.56982987
O	3.23331974	-2.33101799	-2.38827091
O	5.71448379	-1.42355121	-3.17588465
O	3.34278025	0.36930683	2.39025515
C	1.15549692	4.66980001	-1.06088201
C	1.93056791	5.43983486	-0.04034607
C	3.19991100	5.84767372	-0.17795170
C	3.88087496	6.60776584	0.91366872
C	4.04043464	5.58110169	-1.37882811
H	6.71701295	0.56027550	-1.66026896
H	5.53024349	1.52240743	0.30792969
H	2.21564358	-1.17763459	-0.36895826
H	-1.31031095	0.24553743	0.92575201
H	-2.21891946	1.61268734	0.29513266
H	0.48310904	0.53337860	-0.74171925
H	1.75245928	0.55004609	3.22696251
H	-2.02527632	3.43816471	-0.95048911
H	-1.21657708	4.21378019	-2.31399076
H	-2.07982541	2.69922938	-2.54351148
H	-0.17911010	2.14338784	-3.70393230
H	1.10088228	3.22178394	-3.17390106
H	-2.37841656	0.69228711	-1.91093224
H	-0.97334788	0.15713009	-2.82048623
H	-0.56523170	-1.72860662	-1.24965864
H	-3.49151172	-3.55444967	-0.17038471
H	-1.74983157	-3.63831628	-0.42980349
H	-3.77828944	-0.01664397	0.12992677
H	-4.23820156	-0.57993693	-1.48147080
H	-4.67365971	-1.52278740	-0.05382263
H	-1.34766147	1.54023372	3.34781467
H	-0.88085289	3.24330072	3.31633476
H	-2.82216428	3.84322506	1.91805004
H	-5.54489663	1.88652898	1.17765531
H	-5.17202297	3.54237074	1.66505903
H	-5.88947286	2.41902702	2.82586035
H	-2.99066915	0.03711746	2.45678953
H	-4.70200750	0.11139581	2.87777775
H	-3.48666279	0.64229344	4.04277073
H	-3.08736385	-2.24290153	1.98419048
H	-2.47395370	-3.89476239	2.00694764
H	-0.91752532	-1.36241103	2.21794506
H	1.47474446	-1.43071413	2.25563583
H	2.26386779	-2.70248404	1.30669882
H	1.90405676	-2.96601335	3.01422128
H	-0.69398683	-5.04974400	1.38996125
H	0.43491336	-4.51470244	0.14035550
H	1.04446194	-5.08231448	1.70069451
H	2.35191939	1.16317557	-2.22632061
H	1.08777694	0.14102207	-2.90739331
H	1.69101489	0.69615698	-5.17281552
H	4.99214644	1.65151922	-6.04008248
H	3.91936309	3.00849081	-6.35946739
H	3.38655655	1.37435409	-6.74745893
H	3.52285479	3.32911593	-3.05750251
H	5.03690614	3.08684523	-3.94989511
H	4.47670641	1.88655293	-2.77392939
H	3.73850775	-2.66841784	-3.20592528
H	6.55605727	-0.95822819	-3.44657447
H	1.74899818	4.52771857	-1.99052947
H	0.26082228	5.25680642	-1.36184341
H	1.37229091	5.67272137	0.86524128
H	3.24229098	6.76933046	1.79207322
H	4.21235177	7.59762768	0.56836731
H	4.77854679	6.07459261	1.26246982
H	3.52046070	5.82596474	-2.31497311
H	4.98323493	6.14133118	-1.37841187
H	4.30394932	4.50813885	-1.42868621

OC-(AM)$_2$ complex in the DMSO

Energy = -2617.19089169 a.u. #Cluster: 1

O	1.63711575	-3.12024551	-3.36866533
N	3.24858928	-1.83164599	-4.23107298
C	1.94883085	-2.24356049	-4.18456443
C	0.98117244	-1.65726483	-5.12948227
C	-0.32682596	-1.70729372	-4.87210188
H	1.39243819	-1.19421103	-6.02726247
H	3.55880773	-1.05782687	-4.79632609
H	3.92252776	-2.20175401	-3.57824320
H	-1.08142407	-1.29191046	-5.53050742
H	-0.74313641	-2.16564245	-3.97728796
O	2.74477077	-0.07923545	4.27611446
N	4.78686011	-0.60127979	3.51187216
C	3.68405584	-0.88454057	4.25766895
C	3.67683161	-2.11868055	5.06656213

C	2.63811706	-2.41301718	5.85051831
H	4.55586360	-2.76100682	4.98716864
H	5.55155644	-1.24728835	3.39539294
H	4.82347542	0.23987175	2.95508083
H	2.58754959	-3.30632756	6.46313530
H	1.75727050	-1.78300636	5.94059221
C	4.59978912	-1.83368930	-1.08712877
C	5.52373146	-0.84948346	-0.75320649
C	5.11388476	0.22484038	0.03604918
C	3.79076195	0.31548736	0.46188455
C	2.85945029	-0.67693449	0.13625333
C	3.26620456	-1.76472145	-0.62297376
C	3.37293044	1.49275465	1.25563589
C	2.05098744	2.05383516	0.94368995
C	0.92546269	1.76842274	1.64624707
C	-0.44482067	2.23822917	1.21635679
C	-0.26480670	3.56026461	0.47254904
C	0.70495621	3.49462439	-0.69743709
C	2.04486652	2.93328098	-0.22550130
C	0.05571114	2.53476800	-1.76623973
C	-1.01368659	1.24112680	0.19289487
C	-0.20258205	1.14987419	-1.10836395
O	0.86431675	1.02326390	2.73138903
C	-1.27136071	3.16845266	-2.22162192
C	0.95068556	2.39331540	-3.02244814
C	-0.95822896	0.19802595	-2.05930444
C	-0.99615098	-1.17852895	-1.48402348
C	-2.11722621	-1.82962692	-1.14775710
C	-2.05258130	-3.21572857	-0.57852535
C	-3.48664488	-1.25878582	-1.31318112
O	3.07301195	3.21753190	-0.79353351
O	-0.86628197	4.55403884	0.77907998
C	-1.38377669	2.38191098	2.42855958
C	-2.78614138	2.66795312	2.00253217
C	-3.77543163	1.76461502	1.95634596
C	-5.13816550	2.14243243	1.47667148
C	-3.62517984	0.33026427	2.33449692
C	-2.29458591	-3.20343829	0.94392396
C	-1.16559032	-2.55333852	1.67522787
C	0.03496747	-3.11307092	1.87623970
C	1.10899290	-2.39562517	2.62501175
C	0.43242992	-4.45724108	1.36953540
C	2.02386107	1.29351421	-2.99015601
C	2.79710324	1.31676683	-4.26840029
C	4.11414178	1.54642370	-4.36055068
C	4.81257904	1.49898616	-5.67949543
C	4.98350532	1.85320500	-3.19023170
O	2.38895959	-2.77559280	-0.82036593
O	4.89926204	-2.88538124	-1.88274560
O	4.09767746	1.96631858	2.10555367
C	0.95412777	4.91747084	-1.23355247
C	1.68792584	5.75549524	-0.23689157
C	2.89069407	6.30898072	-0.44613762
C	3.55458281	7.12745644	0.61212149
C	3.66172562	6.15876224	-1.71373884
H	6.55409676	-0.91877605	-1.10230559
H	5.83415563	1.00232481	0.30784682
H	1.82065515	-0.62134241	0.47323764
H	-1.08320900	0.23119008	0.65461255
H	-2.06418185	1.51363991	-0.04110585
H	0.79016546	0.68897056	-0.87213338
H	1.74728696	0.68000355	3.12725237
H	-1.96365328	3.33708933	-1.39020349
H	-1.11348792	4.13256972	-2.71964988
H	-1.79490278	2.53190586	-2.94587794
H	0.30294959	2.21772880	-3.90601586
H	1.45256276	3.36124712	-3.22830603
H	-1.97578893	0.58642734	-2.26163069
H	-0.46643357	0.17300933	-3.05453195
H	-0.01574058	-1.63816099	-1.33965096
H	-2.80101775	-3.86807471	-1.07033773
H	-1.06840235	-3.68430989	-0.78821837
H	-4.26147343	-1.86408285	-0.82481008
H	-3.56274180	-0.24574113	-0.89238188
H	-3.76056735	-1.18548361	-2.37615121
H	-1.33652143	1.45917249	3.04953917
H	-1.01302362	3.19666771	3.08863655
H	-2.95670236	3.69993155	1.69670322
H	-5.45479554	1.50790320	0.63510681
H	-5.20253245	3.18302525	1.13199343
H	-5.89276068	2.01835527	2.26729537
H	-3.34168492	-0.27727156	1.45904579
H	-4.55856919	-0.09946481	2.72408894
H	-2.85534012	0.16683496	3.10263412
H	-3.25071620	-2.68697849	1.16286254
H	-2.43898110	-4.24577611	1.29731733
H	-1.38008860	-1.55046929	2.03756350
H	0.82834379	-1.37188963	2.90732254
H	2.02933377	-2.33397114	2.02359804
H	1.37009852	-2.92447382	3.55277839
H	-0.42409973	-5.11255703	1.16703492
H	0.98795073	-4.34828547	0.42082657
H	1.09417221	-4.98784632	2.06602706
H	2.68834036	1.41370858	-2.11246842
H	1.56148793	0.29053527	-2.87145571
H	2.19610108	1.12519899	-5.15583686
H	5.63553535	0.76802116	-5.66925203
H	5.25988319	2.47212970	-5.93075563
H	4.15147899	1.22648103	-6.51232811
H	4.52711977	2.61569320	-2.53528449
H	5.97348248	2.22691512	-3.47842214
H	5.14552791	0.96084461	-2.56641592
H	2.20719441	-2.93017785	-1.80364324
H	5.86789837	-2.90291053	-2.14657194
H	1.51061555	4.85540069	-2.19414075
H	-0.01431834	5.40405231	-1.48136649
H	1.16138435	5.89722223	0.70528516
H	2.92548972	7.27930238	1.49915707
H	3.83443220	8.12270838	0.23817398
H	4.48180480	6.64316637	0.95587922
H	3.14321659	6.62743184	-2.56202949
H	4.66375782	6.60193144	-1.66315212
H	3.79510685	5.09290768	-1.96991889

Energy = -2617.19029291 a.u. #Cluster: 2

O	3.91751048	-2.97997872	-3.97688241
N	2.37406496	-2.26268273	-5.41842872
C	3.67065468	-2.45437681	-5.08368946
C	4.73185164	-2.09435058	-6.03667524
C	5.98887120	-1.89868781	-5.63512280
H	4.42856887	-1.99885595	-7.08339063
H	2.08939754	-1.79725735	-6.26746251
H	1.62788871	-2.49552252	-4.77760033
H	6.79442337	-1.63546491	-6.31471105

H	6.31964887	-1.98245843	-4.60453911
O	2.11437968	-0.37102537	4.44479856
N	4.05456532	-1.13740941	3.62754783
C	2.87520849	-1.33267170	4.28109856
C	2.56868375	-2.67316485	4.81378342
C	1.30332767	-3.04471327	5.01498902
H	3.41932637	-3.32313390	5.02272673
H	4.68914637	-1.88602038	3.40667305
H	4.27691881	-0.22729144	3.24726449
H	1.02025274	-4.01740490	5.40053618
H	0.45042199	-2.40665213	4.80041571
C	4.99101811	-1.21076316	-1.30265283
C	5.78289832	-0.33714631	-0.55761612
C	5.18329270	0.49397087	0.38628965
C	3.80288326	0.45890932	0.56975048
C	3.00307663	-0.42717992	-0.15595309
C	3.59655700	-1.26870533	-1.09048871
C	3.16548606	1.38562530	1.52945309
C	1.90954288	2.01716454	1.11896735
C	0.75286660	1.87629585	1.82299834
C	-0.56351465	2.44036863	1.34153659
C	-0.26572391	3.68570566	0.50884283
C	0.69795175	3.45108699	-0.64404274
C	1.98382348	2.82175526	-0.10248280
C	-0.04669694	2.48456541	-1.64474722
C	-1.19787406	1.41345092	0.38739769
C	-0.38279838	1.16688345	-0.89367215
O	0.60482556	1.19716245	2.93774071
C	-1.34237514	3.18105952	-2.10362992
C	0.78244054	2.21291689	-2.92242253
C	-1.16938499	0.16651032	-1.77084340
C	-1.23665009	-1.14616218	-1.06264981
C	-2.30566160	-1.62329947	-0.41188077
C	-2.21202582	-2.90529831	0.36586538
C	-3.63940882	-0.96054266	-0.35976687
O	3.04455931	3.01954030	-0.64592499
O	-0.78014607	4.74621226	0.74123930
C	-1.49482741	2.75799035	2.52532406
C	-2.88072574	3.06560979	2.06214899
C	-3.93402908	2.25385713	2.23096947
C	-5.28439705	2.62674222	1.71739346
C	-3.85768490	0.93543067	2.92536778
C	-1.55799246	-2.65230941	1.74167146
C	-0.07983538	-2.48462671	1.60081154
C	0.79275906	-3.50178769	1.59344587
C	2.25519368	-3.27193412	1.40561492
C	0.38703017	-4.92840936	1.75783636
C	1.85932087	1.12047448	-2.84285507
C	2.58507214	1.05708750	-4.14827513
C	3.85482132	1.44157407	-4.33390536
C	4.50195570	1.34955098	-5.67721724
C	4.72385358	1.98086384	-3.24993551
O	2.76455261	-2.13022658	-1.71775655
O	5.64465161	-1.98929801	-2.19108016
O	3.65744271	1.59368178	2.62277540
C	1.07356722	4.80058373	-1.28499966
C	1.83831332	5.66856576	-0.33838749
C	3.07379432	6.13698284	-0.56380317
C	3.76450347	6.99896276	0.44151437
C	3.85702047	5.84567396	-1.79890448
H	6.85892641	-0.30993090	-0.72541452
H	5.79768608	1.18296007	0.97014052
H	1.92163218	-0.47804912	-0.00731794
H	-1.34593939	0.45141057	0.92491383
H	-2.22513536	1.73888095	0.12107656
H	0.58505965	0.67714022	-0.61378720
H	1.42861804	0.71474423	3.32521132
H	-1.99336916	3.46279471	-1.26978815
H	-1.13422457	4.09128430	-2.67853218
H	-1.93550999	2.53030789	-2.75828520
H	0.08779476	1.94830825	-3.74730817
H	1.26406766	3.15540703	-3.25430015
H	-2.17441439	0.56427609	-2.00910805
H	-0.67535335	0.04125165	-2.75656436
H	-0.29670047	-1.70243993	-1.06561143
H	-3.21129567	-3.35957799	0.50874340
H	-1.62550383	-3.66324890	-0.19128799
H	-3.66493794	0.01196466	-0.86939555
H	-4.41277803	-1.58672816	-0.82918637
H	-3.95935793	-0.78518391	0.67845619
H	-1.49598434	1.90952682	3.24486861
H	-1.08717482	3.62181047	3.09627338
H	-2.98062859	4.01985357	1.54686114
H	-5.61532066	1.92875583	0.93269523
H	-5.31882550	3.63387885	1.28087076
H	-6.04430796	2.59248151	2.51159290
H	-3.02707857	0.31978371	2.54854721
H	-4.77268930	0.33925457	2.80978052
H	-3.69725151	1.06227614	4.00768086
H	-2.01005482	-1.75651138	2.21236930
H	-1.79916116	-3.49297155	2.42325363
H	0.24754516	-1.45507561	1.47783867
H	2.55308162	-2.23602915	1.61150966
H	2.54759724	-3.48350330	0.36312311
H	2.86161176	-3.92116435	2.05121915
H	0.11024624	-5.14048378	2.80157375
H	-0.48536190	-5.18278986	1.13898366
H	1.18531833	-5.63141910	1.48767795
H	2.55880079	1.31299888	-2.00648833
H	1.41001251	0.12804182	-2.62534752
H	1.98929993	0.66860475	-4.97132306
H	5.42748594	0.75657227	-5.63479644
H	4.77772127	2.34472613	-6.05527748
H	3.85870117	0.88532371	-6.43562703
H	4.18814611	2.71886092	-2.62850798
H	5.62921295	2.46817896	-3.62942152
H	5.04685771	1.17851192	-2.56784147
H	3.13139935	-2.46788393	-2.59084160
H	5.03729212	-2.38891089	-2.89350167
H	1.65492833	4.61210054	-2.21420848
H	0.15414462	5.33348179	-1.61063305
H	1.30569776	5.91346187	0.57886398
H	3.14045494	7.22881063	1.31527128
H	4.07496597	7.95876156	0.00419991
H	4.67644327	6.50998126	0.81723332
H	3.36962884	6.25476561	-2.69513465
H	4.87428124	6.25473984	-1.77043697
H	3.95172429	4.75655462	-1.95541168

Energy = -2617.18523403 a.u. #Cluster: 3

O	2.40036463	-0.41659912	4.14001992
N	4.28094065	-1.14737801	3.16242426
C	3.16303787	-1.36352724	3.90920664
C	2.92525014	-2.70875469	4.46537431

1	C	1.68866467	-3.09982911	4.77753177
2	H	3.80146879	-3.34512944	4.59727349
3	H	4.90438130	-1.88617552	2.88353632
4	H	4.45314804	-0.23419598	2.76294642
5	H	1.45614454	-4.07667853	5.18611337
6	H	0.81070054	-2.47453910	4.64021703
7	O	7.46567975	3.86225909	0.94419220
8	N	5.22680627	3.74566347	0.95806396
9	C	6.42063384	3.76461981	0.29807294
10	C	6.38205564	3.67583444	-1.17599201
11	C	7.45801886	3.31058609	-1.87242894
12	H	5.42440679	3.91315748	-1.64692954
13	H	4.35412238	3.62454586	0.46344241
14	H	5.17858055	3.75135898	1.96328460
15	H	7.46941794	3.22493203	-2.95325304
16	H	8.41209587	3.06391257	-1.41874452
17	C	4.59384862	-1.22437788	-1.82976198
18	C	5.47986346	-0.40172883	-1.14610497
19	C	4.98931146	0.43116046	-0.13930426
20	C	3.62856276	0.45182776	0.15439485
21	C	2.73167394	-0.38120202	-0.52379764
22	C	3.21528711	-1.22300369	-1.51573958
23	C	3.12728794	1.37145382	1.19853606
24	C	1.86079935	2.06683257	0.95418959
25	C	0.78007522	1.91333594	1.77142346
26	C	-0.55903488	2.54625109	1.47217393
27	C	-0.31687181	3.82033787	0.66607340
28	C	0.51153436	3.61603966	-0.59485554
29	C	1.83389631	2.95718867	-0.20279218
30	C	-0.35021943	2.70401576	-1.54941344
31	C	-1.33475677	1.59217229	0.54781374
32	C	-0.65602849	1.36946998	-0.81410987
33	O	0.72919327	1.16360998	2.84622850
34	C	-1.66075921	3.44946110	-1.86437389
35	C	0.35427162	2.45417589	-2.90432374
36	C	-1.55281650	0.42163267	-1.64242624
37	C	-1.58461261	-0.91323932	-0.97363956
38	C	-2.58931584	-1.38108695	-0.22126613
39	C	-2.44770579	-2.68732705	0.50727138
40	C	-3.88769130	-0.68353637	-0.00178953
41	O	2.85287736	3.21425291	-0.80606645
42	O	-0.77049504	4.88209690	0.99643512
43	C	-1.33601461	2.83512038	2.76900509
44	C	-2.76050413	3.18060909	2.48436468
45	C	-3.80248848	2.38129968	2.75314957
46	C	-5.19645538	2.79290942	2.41570069
47	C	-3.67213740	1.04106959	3.39561870
48	C	-1.66119278	-2.48607680	1.82081592
49	C	-0.19930624	-2.34101313	1.54669440
50	C	0.64946046	-3.37244601	1.43899117
51	C	2.09359264	-3.16334175	1.12596761
52	C	0.23629514	-4.79602128	1.61062298
53	C	1.41163076	1.34248715	-2.93785694
54	C	2.03607922	1.27998053	-4.29444006
55	C	3.29873547	1.63735539	-4.56438222
56	C	3.86485198	1.50803734	-5.94030235
57	C	4.23816481	2.17739195	-3.54107850
58	O	2.31720054	-2.02286369	-2.13931429
59	O	4.96351588	-2.08015767	-2.80959663
60	O	3.74781138	1.53957455	2.23112683
61	C	0.84112218	4.98742178	-1.21739709
62	C	1.75046802	5.78685813	-0.34052946
63	C	2.94840332	6.25850791	-0.71369674
64	C	3.79276345	7.04714844	0.23268708
65	C	3.53885067	6.04245363	-2.06616038
66	H	6.54210113	-0.41092041	-1.39049376
67	H	5.68050541	1.07238211	0.41257933
68	H	1.66524196	-0.38640980	-0.28786135
69	H	-1.47481113	0.61521819	1.06014915
70	H	-2.36623159	1.97354463	0.39772203
71	H	0.31802087	0.84324023	-0.64427491
72	H	1.58927881	0.66918250	3.13714689
73	H	-2.21636153	3.73139330	-0.96411425
74	H	-1.48056702	4.36706720	-2.43733579
75	H	-2.33599961	2.83264966	-2.47087043
76	H	-0.41581561	2.22230293	-3.66961438
77	H	0.82309347	3.39663945	-3.25445916
78	H	-2.56577904	0.85175584	-1.76352413
79	H	-1.16125826	0.32127052	-2.67583020
80	H	-0.66929283	-1.49524646	-1.09605983
81	H	-3.43785886	-3.12832172	0.73303410
82	H	-1.93157562	-3.43888514	-0.12323498
83	H	-3.95671316	0.28492510	-0.51549082
84	H	-4.73290313	-1.29301306	-0.35501477
85	H	-4.06113199	-0.48974043	1.06771281
86	H	-1.26810066	1.96253045	3.45573086
87	H	-0.84591121	3.67125356	3.31623406
88	H	-2.90086433	4.15282466	2.01387456
89	H	-5.63122438	2.12788713	1.65338184
90	H	-5.26212836	3.81520141	2.01983480
91	H	-5.85762588	2.74292271	3.29316595
92	H	-2.87995662	0.43533519	2.93104261
93	H	-4.59692423	0.45105599	3.34197694
94	H	-3.41792495	1.13436672	4.46347647
95	H	-2.04753280	-1.59623432	2.35693157
96	H	-1.85437503	-3.34136271	2.49979896
97	H	0.13616806	-1.31466754	1.41828414
98	H	2.43003002	-2.13895134	1.32948137
99	H	2.28999651	-3.35788508	0.05845702
100	H	2.74254666	-3.83793105	1.70096393
101	H	0.10453093	-5.03949735	2.67599434
102	H	-0.71927872	-5.01363895	1.11253544
103	H	0.97400687	-5.50272936	1.20913935
104	H	2.17922971	1.50488496	-2.15636246
105	H	0.96069366	0.35404669	-2.70368618
106	H	1.37856424	0.90055456	-5.07294896
107	H	4.74295457	0.84455127	-5.94320993
108	H	4.19866597	2.48032136	-6.33089289
109	H	3.15037400	1.09608129	-6.66432151
110	H	3.75342593	2.94869876	-2.91852791
111	H	5.13983370	2.62113655	-3.97910922
112	H	4.56763139	1.38461746	-2.85024632
113	H	2.73549636	-2.50972863	-2.90515300
114	H	5.92122831	-1.96381001	-3.07775782
115	H	1.28833461	4.83282773	-2.22360363
116	H	-0.09628354	5.55875236	-1.39204021
117	H	1.36028693	5.97735658	0.65788784
118	H	3.28126693	7.27963172	1.17623801
119	H	4.11085449	8.00264318	-0.20781106
120	H	4.70853970	6.49396838	0.49310376
121	H	2.99375678	6.60519400	-2.83746297
122	H	4.59068905	6.34900771	-2.12946642
123	H	3.49229496	4.97846713	-2.35477430
124				

Energy = -2617.18418481 a.u. #Cluster: 4

O	3.11593190	-3.05596938	-2.67544990
N	3.13879355	-1.42133714	-4.20554250
C	2.46461428	-2.32887881	-3.44017846
C	1.00013578	-2.41131990	-3.57559277
C	0.30197415	-3.34631713	-2.92902414
H	0.52788647	-1.67784278	-4.23261472
H	2.67480348	-0.74789402	-4.79647912
H	4.13556609	-1.30356069	-4.10094209
H	-0.77540035	-3.44462005	-3.00486873
H	0.75133913	-4.08875863	-2.27467961
O	-2.17857077	8.65135465	1.27760941
N	-0.65089163	7.13685243	0.64569714
C	-1.63009416	7.56911982	1.49301355
C	-1.96385067	6.70924807	2.64863139
C	-3.17737033	6.73623353	3.19831658
H	-1.16224901	6.05856618	3.01038043
H	-0.21506557	6.23267304	0.76559228
H	-0.39544560	7.66372231	-0.17293420
H	-3.46072407	6.12408647	4.04734953
H	-3.98419222	7.37049982	2.84595484
C	6.33397356	0.13564682	-1.74991776
C	6.23052494	1.25635227	-0.93200826
C	5.18141728	1.34316511	-0.01498497
C	4.25735165	0.30790916	0.07901824
C	4.35923425	-0.82960730	-0.72572388
C	5.39754003	-0.91548942	-1.64379371
C	3.12845729	0.36169956	1.03386200
C	2.00466554	1.24895464	0.84028766
C	0.91129347	1.16037167	1.68025078
C	-0.30812366	2.02964737	1.54229224
C	0.14589432	3.35732388	0.93881600
C	0.87473220	3.22045582	-0.38337566
C	2.03428435	2.22702982	-0.24642585
C	-0.19477746	2.68818277	-1.41412083
C	-1.26776691	1.38983068	0.52620962
C	-0.70760634	1.31327414	-0.90381162
O	0.82611759	0.29650290	2.65993667
C	-1.35112523	3.70332523	-1.47271924
C	0.38311568	2.58303142	-2.84535739
C	-1.81910650	0.71966234	-1.79993061
C	-1.98631729	-0.72094739	-1.44862078
C	-3.00892350	-1.24269117	-0.75889819
C	-2.99189174	-2.69381702	-0.37038950
C	-4.20758727	-0.48203671	-0.30758068
O	2.95355426	2.28190833	-1.02646357
O	-0.09181930	4.40650034	1.47507086
C	-0.99928360	2.22382868	2.90555562
C	-2.29338978	2.95376385	2.76101473
C	-3.50476789	2.38971970	2.86460086
C	-4.74554203	3.19874839	2.68139129
C	-3.72057637	0.94231010	3.15279098
C	-2.29531940	-2.86973698	0.99555956
C	-0.83945808	-2.55069138	0.89626371
C	0.11174127	-3.44077378	0.58258549
C	1.54535225	-3.03887186	0.48393607
C	-0.17275342	-4.87948073	0.30950161
C	1.10353361	1.27680383	-3.21772005
C	1.65641569	1.40893050	-4.60106455
C	2.91429044	1.78483194	-4.87620908
C	3.39916567	1.89190782	-6.28390362
C	3.91409502	2.12531696	-3.82740411
O	5.55818894	-1.98990280	-2.45106232
O	7.31072703	-0.01828915	-2.67384892
O	3.14117992	-0.42177614	1.99016837
C	1.49850862	4.57055351	-0.78965637
C	2.47370388	5.05022855	0.23650628
C	3.79090763	5.20210434	0.03814178
C	4.68047828	5.65668403	1.14945354
C	4.48692986	4.92536074	-1.24932010
H	6.95338421	2.06752816	-1.01867386
H	5.07981692	2.23278770	0.60958293
H	3.63158303	-1.63985611	-0.64731810
H	-1.55105694	0.37085077	0.86795001
H	-2.22457490	1.95625256	0.52312742
H	0.15556002	0.59983034	-0.91080539
H	1.71406644	-0.28322186	2.74376582
H	-1.80943437	3.88575414	-0.49527799
H	-1.02256119	4.67139054	-1.87011469
H	-2.15511530	3.35272051	-2.13298887
H	-0.43873823	2.73780621	-3.57598411
H	1.07859252	3.42874720	-3.02339287
H	-2.75917802	1.29286354	-1.68521080
H	-1.55954133	0.82565381	-2.87355911
H	-1.15020590	-1.35117111	-1.75769320
H	-4.02017506	-3.10218067	-0.31867067
H	-2.47099417	-3.30649224	-1.13306616
H	-4.04094475	0.60282645	-0.26361363
H	-5.05604049	-0.64873785	-0.98936807
H	-4.53970885	-0.79364010	0.69361661
H	-1.14822357	1.23687964	3.39540301
H	-0.32288180	2.78941799	3.58554714
H	-2.18469858	4.01734140	2.54613735
H	-5.31670284	2.85161419	1.80637629
H	-4.54555459	4.26795651	2.52816246
H	-5.41452501	3.11563554	3.55039774
H	-3.23105673	0.30204430	2.40356825
H	-4.78249651	0.66191355	3.16168976
H	-3.30865276	0.65872780	4.13371637
H	-2.78520035	-2.21919523	1.74822029
H	-2.45551855	-3.90660411	1.35571302
H	-0.59436005	-1.50602859	1.07808165
H	1.72210222	-2.00694270	0.81312092
H	1.90447116	-3.11826364	-0.55661995
H	2.19026780	-3.68785604	1.09464874
H	-0.52792780	-5.39509487	1.21462730
H	-0.95954296	-5.00107626	-0.44975964
H	0.70775120	-5.42929698	-0.04907333
H	1.90561395	1.04413175	-2.49030767
H	0.40369227	0.41768926	-3.17273987
H	0.94352563	1.19403678	-5.39398259
H	4.27607734	1.24967081	-6.45751944
H	3.71073971	2.92068198	-6.51930893
H	2.64094154	1.60928759	-7.02575974
H	3.58792944	3.00129124	-3.24027183
H	4.90778229	2.35155662	-4.23012354
H	4.03512420	1.31680069	-3.08712101
H	4.70402557	-2.55530402	-2.47231749
H	7.88923587	0.79483281	-2.74553244
H	1.99078339	4.45615120	-1.78258448
H	0.70461684	5.32843541	-0.94776737
H	2.03430059	5.27840616	1.20691508
H	4.14147977	5.83476648	2.08941494
H	5.20129024	6.59030408	0.89301237

H	5.45753090	4.90601145	1.36207360
H	3.94188181	5.32492691	-2.11524811
H	5.50164822	5.34079326	-1.28722481
H	4.58104327	3.83458689	-1.40885861

Energy = -2617.18369056 a.u. #Cluster: 5

O	6.91860257	-1.50416804	-5.06324194
N	4.69975731	-1.41149749	-5.37675073
C	5.97786479	-1.23101766	-5.81798405
C	6.18451212	-0.76830121	-7.20379053
C	7.31298227	-0.14921306	-7.55275918
H	5.37387033	-0.96519014	-7.90790414
H	3.89707063	-1.09274628	-5.89320719
H	4.53040379	-1.70722969	-4.42871686
H	7.51105228	0.20767860	-8.55758078
H	8.12681930	0.05235500	-6.86233399
O	2.74951002	-3.25596679	-3.29836681
N	1.37326810	-3.45768336	-1.54230202
C	1.59377643	-3.21092892	-2.86518955
C	0.44172442	-2.90129631	-3.73150379
C	0.56757137	-2.06211551	-4.76013907
H	-0.49365663	-3.41144366	-3.49484824
H	0.45668001	-3.44000565	-1.12314331
H	2.11916690	-3.77263343	-0.94002467
H	-0.24944591	-1.81319066	-5.42927586
H	1.49444356	-1.55760245	-5.01544698
C	6.15563811	-0.28734784	-1.75397084
C	6.24597994	0.80530396	-0.89538281
C	5.21786765	1.04262579	0.01833726
C	4.11901894	0.19178009	0.06340359
C	4.01134852	-0.90910620	-0.79080236
C	5.04019295	-1.15006258	-1.68966182
C	3.01911608	0.40927169	1.03116264
C	1.93034596	1.31029231	0.73093747
C	0.77282094	1.26246580	1.47993690
C	-0.45142449	2.07521378	1.16215429
C	0.01801672	3.38728334	0.53334744
C	0.91079809	3.20728539	-0.68097704
C	2.07970125	2.27133769	-0.36172438
C	0.00226076	2.57009050	-1.80072748
C	-1.26597297	1.34723900	0.07944887
C	-0.54480542	1.21540896	-1.27228092
O	0.62195444	0.47987729	2.52115931
C	-1.15871887	3.53990820	-2.08534655
C	0.77239528	2.38433446	-3.13039199
C	-1.53838650	0.56107571	-2.25981212
C	-1.69289584	-0.87624724	-1.89452654
C	-2.76155686	-1.42596271	-1.30338690
C	-2.73645479	-2.87306208	-0.90244935
C	-4.02219223	-0.69982556	-0.98567556
O	3.10239914	2.35489293	-0.99670847
O	-0.33236213	4.45446284	0.95823556
C	-1.29750952	2.31076837	2.42761164
C	-2.61998963	2.92060317	2.09817679
C	-3.78630560	2.25957984	2.11842445
C	-5.06287056	2.93947825	1.75140528
C	-3.91372503	0.81957616	2.48690910
C	-2.31250187	-3.02178283	0.57214598
C	-0.89145359	-2.62192664	0.78683609
C	0.00367755	-3.32937667	1.49033345
C	1.40095380	-2.83738115	1.67436112
C	-0.30690791	-4.63217082	2.14432443
C	1.57006259	1.08090352	-3.32281412
C	2.23559395	1.14132687	-4.66163688
C	3.45170396	1.66501988	-4.87127565
C	4.01582941	1.77980945	-6.24934186
C	4.31090056	2.19604118	-3.77820923
O	5.06427146	-2.21819031	-2.51897606
O	7.18898754	-0.50332656	-2.59373189
O	3.00910899	-0.25751379	2.06873208
C	1.51595748	4.57029020	-1.07505571
C	2.39214203	5.10439141	0.01237677
C	3.71289794	5.31046318	-0.08630886
C	4.49354145	5.82325318	1.08056834
C	4.51886578	5.03849592	-1.30950112
H	7.10629880	1.47173162	-0.95200058
H	5.26945338	1.91005719	0.67885981
H	3.14504707	-1.56503541	-0.75408806
H	-1.55651119	0.33902942	0.44456526
H	-2.23185545	1.87894268	-0.06481873
H	0.32438814	0.52188703	-1.14426922
H	1.49604638	-0.07394513	2.72217490
H	-1.75459087	3.76083238	-1.19360087
H	-0.80038106	4.49645189	-2.48515020
H	-1.85108864	3.12976209	-2.83123322
H	0.05287817	2.46510889	-3.97213008
H	1.46557692	3.23979899	-3.27057834
H	-2.50479449	1.10101942	-2.25751264
H	-1.17315305	0.65248693	-3.30390805
H	-0.81352963	-1.47889508	-2.12061375
H	-3.73485641	-3.33420672	-1.04675003
H	-2.05517709	-3.46178921	-1.54731667
H	-3.88124749	0.38466067	-0.88006227
H	-4.76971783	-0.84947575	-1.78053980
H	-4.48095496	-1.05359230	-0.05069803
H	-1.42890880	1.35181792	2.97638405
H	-0.73944157	2.97262290	3.12711932
H	-2.57124947	3.97135228	1.81545033
H	-5.53336179	2.45615179	0.88125417
H	-4.93084224	3.99912785	1.49476630
H	-5.79418007	2.89398216	2.57210816
H	-3.34339642	0.17479330	1.80133337
H	-4.95206946	0.46215534	2.46339048
H	-3.53355491	0.62229308	3.50132483
H	-2.97207319	-2.40039574	1.21592521
H	-2.49859080	-4.06731201	0.89263637
H	-0.62660587	-1.66409243	0.33809378
H	1.56746757	-2.51522009	2.71613924
H	1.64489604	-1.98151532	1.03331031
H	2.14297591	-3.62628978	1.47700195
H	-1.15801945	-4.54773712	2.83644795
H	-0.57680983	-5.40090280	1.40365710
H	0.53492583	-5.03253972	2.72542935
H	2.31556232	0.94273493	-2.51514505
H	0.90294959	0.19857451	-3.26809184
H	1.63226037	0.76603609	-5.48403845
H	5.05053724	1.41355242	-6.30398007
H	4.04000739	2.83270995	-6.57108508
H	3.43261256	1.23469593	-7.00300769
H	3.81135402	3.02194731	-3.24342443
H	5.28087541	2.56623878	-4.12748779
H	4.50740320	1.43336017	-3.00521171
H	4.15720811	-2.66577668	-2.61203525
H	6.91323099	-0.97541835	-3.44974655

H	2.07878443	4.45892048	-2.02761554
H	0.70292206	5.29684464	-1.28795719
H	1.86501250	5.32909803	0.93860182
H	3.87645450	5.99248608	1.97263031
H	4.99068931	6.77519248	0.84670916
H	5.28258809	5.11054691	1.36715727
H	4.02353429	5.38983731	-2.22443962
H	5.51231564	5.50232822	-1.28073512
H	4.67363040	3.94918511	-1.42813481

Energy = -2617.18309061 a.u. #Cluster: 6

O	7.40029491	-2.88026930	-3.74922401
N	5.66200883	-4.12897515	-4.40351280
C	7.00126952	-3.89394818	-4.34324099
C	7.92479940	-4.86923020	-4.94964162
C	9.23429703	-4.62224373	-5.01357005
H	7.47925199	-5.78794639	-5.33730994
H	5.26098736	-4.87917311	-4.94579367
H	5.01031486	-3.47449387	-3.99973019
H	9.94994071	-5.31018436	-5.45070124
H	9.69409073	-3.71408357	-4.63490225
O	1.79646184	-2.14894714	-4.04077772
N	3.12405603	-0.79652439	-5.25336662
C	1.89336887	-1.29130150	-4.93226781
C	0.71697450	-0.81591284	-5.68295273
C	-0.51120732	-0.96931656	-5.18529913
H	0.90991559	-0.34418898	-6.64767437
H	3.24271550	-0.02890758	-5.89558011
H	3.94478219	-1.04685389	-4.72067458
H	-1.40753831	-0.63492343	-5.69516997
H	-0.71526789	-1.44172631	-4.22716146
C	5.31676903	-0.41338443	-2.49637064
C	5.69928150	0.72457937	-1.79264624
C	4.99554576	1.08007971	-0.64022188
C	3.93102309	0.29604089	-0.20922438
C	3.55255124	-0.86284235	-0.89323315
C	4.26247134	-1.22264193	-2.03029899
C	3.13200038	0.64294775	0.98882140
C	2.01252717	1.55694822	0.86518662
C	0.92135860	1.40297058	1.68726945
C	-0.39282811	2.08279296	1.42615779
C	-0.05974922	3.47880332	0.89588524
C	0.79926107	3.47571295	-0.35768022
C	2.05472343	2.62229874	-0.13782587
C	-0.11143839	2.85705011	-1.48516365
C	-1.11506443	1.31489119	0.30280408
C	-0.43811400	1.39520276	-1.07667012
O	0.90576813	0.61164070	2.73803049
C	-1.40387041	3.69306339	-1.57308305
C	0.54305122	2.92787341	-2.88327872
C	-1.36865083	0.69636976	-2.09133048
C	-1.46619822	-0.75372930	-1.75101805
C	-2.57543733	-1.36031378	-1.30835744
C	-2.55245278	-2.81657210	-0.94928625
C	-3.88337065	-0.66569549	-1.12885559
O	3.06586318	2.86406330	-0.75059565
O	-0.46782625	4.47469305	1.42851533
C	-1.25960690	2.13870919	2.69699404
C	-2.63244454	2.64481404	2.39883771
C	-3.73099184	1.87787180	2.34676394
C	-5.06159082	2.46220012	2.00830772
C	-3.72657734	0.40755340	2.59910473
C	-2.43416455	-2.99894187	0.57712143
C	-1.10511386	-2.54853142	1.09008476
C	0.04562046	-3.21539719	0.92538033
C	1.32308244	-2.70048612	1.50554569
C	0.17157266	-4.49862638	0.17834511
C	1.56329230	1.83875871	-3.24428974
C	2.03386363	2.08249898	-4.64227840
C	3.24790453	2.53471041	-4.98182586
C	3.62322304	2.71853809	-6.41739574
C	4.32472748	2.87232338	-4.01085982
O	3.99822477	-2.38077442	-2.68262921
O	5.89993073	-0.67114289	-3.69178117
O	3.33679153	0.04881160	2.04482604
C	1.26334563	4.91007180	-0.67119716
C	2.15637772	5.44634229	0.40070267
C	3.42398323	5.83913154	0.21111345
C	4.24415712	6.35773843	1.34683103
C	4.12105742	5.78934237	-1.10597032
H	6.52649641	1.33529227	-2.15324472
H	5.26963383	1.98689831	-0.09502405
H	2.71445722	-1.47426056	-0.55919685
H	-1.22627871	0.24716041	0.59251872
H	-2.15766423	1.69071004	0.22045367
H	0.52122156	0.81861032	-1.04362274
H	1.83309623	0.16512036	2.90327929
H	-1.96609542	3.71521952	-0.63403753
H	-1.19621875	4.73236917	-1.85416456
H	-2.08689244	3.29284021	-2.33325545
H	-0.26133133	2.90887352	-3.64996208
H	1.02612670	3.91789491	-3.01329781
H	-2.36112319	1.18671771	-2.10805806
H	-0.97963742	0.81597111	-3.12405214
H	-0.53167975	-1.30561821	-1.85983721
H	-3.46847772	-3.32178788	-1.31494765
H	-1.71058869	-3.33806476	-1.44915879
H	-3.79229768	0.21139178	-0.47121920
H	-4.28184011	-0.30623897	-2.08942423
H	-4.65224872	-1.31196850	-0.68542485
H	-1.29918311	1.13231553	3.16950628
H	-0.77021403	2.79485969	3.45101261
H	-2.68195100	3.71490564	2.20179919
H	-5.46543678	2.01922136	1.08481812
H	-5.03064606	3.54888356	1.85239287
H	-5.80059362	2.27113393	2.80049490
H	-3.10669114	-0.12747635	1.86323894
H	-4.72916478	-0.03803330	2.54667301
H	-3.32407111	0.16510191	3.59484700
H	-3.25413796	-2.44520836	1.07784506
H	-2.60790673	-4.06611687	0.83035955
H	-1.12823587	-1.60334251	1.62899088
H	1.31106040	-1.61452935	1.66000349
H	2.18915179	-2.93692776	0.87077464
H	1.51880400	-3.16341723	2.48583336
H	-0.77177168	-5.05926906	0.12901242
H	0.48458296	-4.31208150	-0.86175836
H	0.92103838	-5.16790287	0.62311269
H	2.40888125	1.82442873	-2.52993179
H	1.11015601	0.82817280	-3.17658975
H	1.28402872	1.86717585	-5.40274475
H	4.54869090	2.17695801	-6.66405390
H	3.80708712	3.77820312	-6.64796817
H	2.85078633	2.37222587	-7.11589918

H	3.94097460	3.03877118	-2.98971498
H	4.87314234	3.77791681	-4.29910797
H	5.05876810	2.05325971	-3.94314890
H	3.06349920	-2.35398238	-3.12616573
H	6.38782008	-1.56582468	-3.69429434
H	1.77670628	4.91746697	-1.65846607
H	0.38142162	5.57618973	-0.78602209
H	1.69341056	5.50741273	1.38373812
H	3.69349639	6.39570026	2.29587994
H	4.61299129	7.37376895	1.14635590
H	5.12844173	5.72339899	1.51363315
H	3.60535974	6.39397952	-1.86468618
H	5.15730226	6.14457720	-1.05827566
H	4.15314359	4.75297269	-1.48973720

Energy = -2617.18285680 a.u. #Cluster: 7

O	1.72360968	-3.10070621	-4.02797081
N	3.16453647	-1.75777153	-5.08954057
C	1.89672031	-2.21049224	-4.86940742
C	0.78793407	-1.64976281	-5.66326734
C	-0.44666048	-1.61619723	-5.15964983
H	1.03777825	-1.27505048	-6.65658989
H	3.36410083	-0.96855688	-5.68216145
H	3.92662950	-2.07009941	-4.50515655
H	-1.29856422	-1.21045529	-5.69379296
H	-0.70051689	-1.99117369	-4.17038471
O	7.49138440	4.13426124	1.13303799
N	5.28283777	3.75347474	1.11335926
C	6.48906386	3.84219619	0.47888425
C	6.51780887	3.56002846	-0.97062558
C	7.66307852	3.29980327	-1.60116156
H	5.55188401	3.57953227	-1.48462283
H	4.43583534	3.55891178	0.60260522
H	5.18643346	3.96881218	2.09257779
H	7.72458481	3.08920697	-2.66333776
H	8.62890085	3.27364174	-1.10708150
C	4.76105704	-1.49589460	-1.94635917
C	5.50976762	-0.39656092	-1.54326385
C	5.01654508	0.43112658	-0.53178366
C	3.78496249	0.15880301	0.04629374
C	3.02197669	-0.94423928	-0.35483368
C	3.51950535	-1.78673006	-1.33840792
C	3.21820776	0.98089680	1.14061266
C	2.09246083	1.84949585	0.82288785
C	0.98658629	1.81784437	1.63448427
C	-0.36131772	2.28283158	1.16441521
C	-0.10707301	3.55593538	0.35358542
C	0.83298696	3.36376894	-0.82903505
C	2.13134570	2.70211138	-0.35984676
C	0.05200999	2.43616811	-1.83240576
C	-0.93002272	1.21460686	0.20675154
C	-0.17976631	1.07200854	-1.12983339
O	0.99131998	1.32337665	2.85507531
C	-1.29667401	3.10284122	-2.16388128
C	0.79918188	2.27039455	-3.17499405
C	-0.97996934	0.09187363	-2.01398762
C	-0.76916699	-1.30140013	-1.52431025
C	-1.72485751	-2.08552072	-1.00897622
C	-1.38749176	-3.46517059	-0.52642469
C	-3.15164299	-1.66970533	-0.87989408
O	3.18069306	2.93850293	-0.91648854
O	-0.61917808	4.60430355	0.63375540
C	-1.31632804	2.53007651	2.34473500
C	-2.69748806	2.84512150	1.87335662
C	-3.73140822	1.99311596	1.92419281
C	-5.07605162	2.38443022	1.40929457
C	-3.63809714	0.61069666	2.47787267
C	-1.61811097	-3.61092380	0.98904513
C	-0.80639650	-2.64034929	1.78371389
C	0.51066264	-2.74294266	2.00867669
C	1.21777281	-1.75942082	2.88352843
C	1.37500890	-3.81204133	1.43518554
C	1.91231358	1.21489374	-3.22991737
C	2.51860835	1.24188510	-4.59563917
C	3.76032501	1.65751451	-4.87776891
C	4.28753823	1.61405104	-6.27467658
C	4.71666701	2.17516232	-3.85993131
O	2.83834745	-2.92533733	-1.60261525
O	5.15926398	-2.34234706	-2.92609865
O	3.56671879	0.80395653	2.29795198
C	1.19139776	4.73616480	-1.42619027
C	1.99703673	5.56410917	-0.47830353
C	3.19527305	6.09343667	-0.76272527
C	3.92125763	6.93415502	0.23534389
C	3.89646309	5.90212106	-2.06510443
H	6.47595637	-0.18947695	-2.00501407
H	5.60443420	1.29019796	-0.20444927
H	2.05211794	-1.16696197	0.09719127
H	-0.95041724	0.22621018	0.71480895
H	-1.99765346	1.44921797	0.00453203
H	0.82220178	0.61166925	-0.93135365
H	1.93531108	1.04341504	3.16069307
H	-1.91957971	3.27420457	-1.27988025
H	-1.16044801	4.07107182	-2.66033517
H	-1.89118943	2.48055414	-2.84543108
H	0.05922371	2.03277631	-3.96900550
H	1.22515029	3.24748595	-3.48358312
H	-2.05074241	0.37370912	-2.03041680
H	-0.65699179	0.16748659	-3.07341924
H	0.26074227	-1.65379246	-1.60419592
H	-1.99929202	-4.21451453	-1.06916225
H	-0.33194833	-3.71980936	-0.75917718
H	-3.26761733	-0.88120362	-0.12138541
H	-3.54937143	-1.26868181	-1.82351741
H	-3.81143294	-2.49547083	-0.58259555
H	-1.31483872	1.64726404	3.02152961
H	-0.92923599	3.36958358	2.96533665
H	-2.81081942	3.84519168	1.45686555
H	-5.36557783	1.76250223	0.54787566
H	-5.12472664	3.42988253	1.07640100
H	-5.85674867	2.25195428	2.17281864
H	-2.80757551	0.04366366	2.03340384
H	-4.55004550	0.02162998	2.30924463
H	-3.46759596	0.62634834	3.56631273
H	-2.69683046	-3.48162216	1.21268083
H	-1.38059938	-4.65318198	1.29183985
H	-1.37894005	-1.81093824	2.19328817
H	0.55965445	-0.95243364	3.23336236
H	2.07558439	-1.30258279	2.37132014
H	1.61684173	-2.25215085	3.78433340
H	0.83884507	-4.76167691	1.30081225
H	1.74561388	-3.51540981	0.43743786
H	2.25715126	-4.02055958	2.05448474
H	2.67201204	1.38955670	-2.44384094

H	1.50922518	0.20156280	-3.02426995
H	1.84687596	0.90121078	-5.38286753
H	5.20442020	1.00828850	-6.33714776
H	4.55134422	2.62001918	-6.63411834
H	3.57437566	1.19898419	-6.99841640
H	4.19832503	2.67907726	-3.02624276
H	5.42905876	2.89528434	-4.28247885
H	5.30617122	1.35472819	-3.42214802
H	2.53063528	-2.97405594	-2.56660662
H	6.06677121	-2.11020970	-3.28465342
H	1.73441997	4.58531803	-2.38575267
H	0.26244782	5.28522308	-1.69557094
H	1.52399917	5.73478993	0.48736974
H	3.34252746	7.11179676	1.15191260
H	4.17963341	7.91877295	-0.18071780
H	4.86567069	6.45880789	0.54060707
H	3.40626769	6.46705389	-2.87126992
H	4.94493870	6.22581844	-2.03896644
H	3.89240068	4.84212508	-2.37052499

Energy = -2617.17782765 a.u. #Cluster: 8

O	2.29414297	-0.89169157	4.28575594
N	4.04054547	-1.72947613	3.15802018
C	2.92651947	-1.89178575	3.92461791
C	2.53943640	-3.25296823	4.34093745
C	1.26109905	-3.54533296	4.58438767
H	3.34521786	-3.98272348	4.43067648
H	4.56174018	-2.50114305	2.77722801
H	4.31472190	-0.80457636	2.85464664
H	0.92101137	-4.52789621	4.89117570
H	0.45289514	-2.82574280	4.48555074
O	-3.05021013	8.78762645	0.82530223
N	-1.27164401	7.47242119	0.45965492
C	-2.37142529	7.82510452	1.18759208
C	-2.67449087	7.03264587	2.39857284
C	-3.91835851	6.94433728	2.86819700
H	-1.82437412	6.53080500	2.87063600
H	-0.72133265	6.65887107	0.70095391
H	-1.02692014	7.95705935	-0.38777232
H	-4.18260183	6.37801274	3.75423103
H	-4.77206058	7.43002532	2.40700914
C	4.62053129	-1.04169374	-1.86594410
C	5.50699542	-0.24091777	-1.15855981
C	5.04229426	0.46679851	-0.04743675
C	3.70495530	0.37972798	0.32446869
C	2.80245299	-0.42084623	-0.38551799
C	3.26383726	-1.13866647	-1.48031322
C	3.21993624	1.15295283	1.48996647
C	2.00444631	1.94735503	1.32414735
C	0.93710092	1.83096978	2.16421034
C	-0.35554027	2.57838052	1.93692637
C	-0.03616680	3.87819340	1.20340874
C	0.74625939	3.69937368	-0.08591805
C	2.01782805	2.90016260	0.21372634
C	-0.21758313	2.92756876	-1.07082235
C	-1.21954002	1.74146573	0.97711881
C	-0.60137975	1.56908538	-0.42067449
O	0.85416061	1.02996186	3.19989079
C	-1.47702321	3.79053524	-1.27904941
C	0.41892522	2.72516390	-2.46620684
C	-1.59807792	0.75925551	-1.28149321
C	-1.71681154	-0.61593561	-0.71281296
C	-2.73056456	-1.05995340	0.04189682
C	-2.67009154	-2.42557052	0.66543721
C	-3.96077326	-0.28254519	0.36147793
O	3.02753417	3.08891495	-0.42297816
O	-0.41640501	4.94167830	1.61615276
C	-1.08605373	2.84346369	3.26590230
C	-2.48032868	3.32356757	3.03209286
C	-3.58419329	2.60267404	3.27234136
C	-4.94073042	3.15582424	2.98745858
C	-3.56314221	1.21889770	3.82906422
C	-1.77321446	-2.40312944	1.92222644
C	-0.32781134	-2.37472386	1.54337327
C	0.40121810	-3.46729232	1.27695033
C	1.83375941	-3.36673176	0.87075102
C	-0.14047088	-4.85524644	1.35596769
C	1.37440181	1.53357812	-2.62055692
C	1.98202198	1.54598963	-3.98624748
C	3.22112675	1.97996101	-4.25465829
C	3.77809268	1.93074863	-5.63949345
C	4.13567791	2.53790351	-3.21910945
O	2.36862682	-1.91864591	-2.13231713
O	4.97193827	-1.78434031	-2.94215276
O	3.80381073	1.10300605	2.55708696
C	1.18985518	5.06552063	-0.64326356
C	2.04213131	5.82181482	0.32365476
C	3.28855189	6.24591030	0.07088029
C	4.06367855	7.00192907	1.09950790
C	4.00255498	6.00845234	-1.21561474
H	6.55019904	-0.16727747	-1.46546083
H	5.73234241	1.09628200	0.51935504
H	1.75176058	-0.48994443	-0.09981642
H	-1.40927841	0.74285712	1.42735213
H	-2.22754160	2.19842515	0.88912184
H	0.33252518	0.95910070	-0.32470264
H	1.65772073	0.41405038	3.40538282
H	-1.98383686	4.03859484	-0.34101092
H	-1.24730235	4.73291455	-1.79079229
H	-2.21544203	3.27336956	-1.90526299
H	-0.39135244	2.61610258	-3.21732698
H	0.95473039	3.64857197	-2.76528907
H	-2.57638233	1.27543073	-1.33176157
H	-1.24983734	0.70819663	-2.33394346
H	-0.85694672	-1.25735857	-0.91397881
H	-3.68051884	-2.78579312	0.93946345
H	-2.28136053	-3.17203105	-0.05603147
H	-3.96936470	0.72405905	-0.07822845
H	-4.86339481	-0.79683337	-0.00117661
H	-4.08225321	-0.15616230	1.44797725
H	-1.08022045	1.92532871	3.89273064
H	-0.52107222	3.60071607	3.85489161
H	-2.54338506	4.33338771	2.62587866
H	-5.44427162	2.57878137	2.19646049
H	-4.92001928	4.20213932	2.65387780
H	-5.59034003	3.11108435	3.87358844
H	-2.83707267	0.57566066	3.31007444
H	-4.53794149	0.71744165	3.76141100
H	-3.28233488	1.22221920	4.89413834
H	-2.02918809	-1.52588738	2.54957015
H	-2.00042536	-3.28706157	2.55223439
H	0.10107657	-1.37768897	1.47766980
H	2.29992132	-2.42299811	1.18031343
H	1.93151519	-3.42431310	-0.22653982

H	2.44136952	-4.18002395	1.28981446
H	-0.16157470	-5.20692170	2.39895296
H	-1.16906457	-4.92484926	0.97471142
H	0.46021374	-5.57633653	0.78646753
H	2.16323344	1.55644555	-1.84318455
H	0.84133832	0.57197528	-2.46570463
H	1.33643613	1.15911939	-4.77006547
H	4.67824221	1.29861406	-5.67797225
H	4.07459287	2.93007822	-5.98951664
H	3.07128412	1.52574594	-6.37482797
H	3.65644106	3.36263202	-2.66476952
H	5.07675809	2.91752857	-3.63116684
H	4.39231344	1.78079714	-2.45937036
H	2.75831223	-2.29188119	-2.97398615
H	5.90437824	-1.59113548	-3.24886712
H	1.73235326	4.89887957	-1.60220709
H	0.30389702	5.67509675	-0.91772906
H	1.57463762	6.02680464	1.28545047
H	3.50216190	7.16867805	2.02850005
H	4.37022194	7.98984977	0.72550236
H	4.98535062	6.46579507	1.37249668
H	3.45735736	6.43285128	-2.07094943
H	5.01250691	6.43609290	-1.23428213
H	4.10899622	4.92599099	-1.40894867

Energy = -2617.17737629 a.u. #Cluster: 9

O	-1.59182352	8.54328232	0.65821503
N	0.11055308	7.18086287	1.18198053
C	-1.22397868	7.43228348	1.04066342
C	-2.16108228	6.33980494	1.38005764
C	-3.31991157	6.19790347	0.73857920
H	-1.83815827	5.66844175	2.18023162
H	0.45410573	6.26408055	1.43802181
H	0.80454346	7.86626417	0.93374582
H	-4.03172953	5.40994780	0.95816586
H	-3.65162071	6.85503226	-0.05900821
O	7.21433708	4.88555245	0.46916869
N	5.63900701	3.36088448	0.93011947
C	6.21479160	4.27218014	0.09134899
C	5.60415278	4.45742497	-1.24143412
C	6.31448292	4.91802985	-2.26965600
H	4.54291106	4.19605933	-1.31848497
H	4.81068760	2.85345457	0.66369734
H	5.98290641	3.21365530	1.86375596
H	5.90047779	5.06948952	-3.25983073
H	7.36537197	5.17736389	-2.20544330
C	5.30114478	-0.62375661	-2.62659754
C	5.88700250	0.07726801	-1.58162870
C	5.15804987	0.27388974	-0.40602178
C	3.85480728	-0.19713738	-0.30915808
C	3.25438653	-0.91011939	-1.35253750
C	3.98765334	-1.13022178	-2.51125892
C	3.05995531	0.01690040	0.92331451
C	2.07644117	1.07792136	0.99119201
C	1.07147775	1.01817052	1.93651382
C	-0.10298878	1.95867457	1.94764679
C	0.35370869	3.29109527	1.35535042
C	0.97764284	3.17699592	-0.02686161
C	2.16367126	2.20963256	0.07523538
C	-0.15545726	2.66268341	-0.99451202
C	-1.16517101	1.39542998	0.98747499
C	-0.68391652	1.30017239	-0.46919207
O	1.01646800	0.10560355	2.87452362
C	-1.29112199	3.70434379	-0.99650952
C	0.33889500	2.54648442	-2.45461671
C	-1.84870272	0.74487383	-1.31959710
C	-2.16129995	-0.64269186	-0.86718847
C	-3.22919790	-1.00226436	-0.14263103
C	-3.37766178	-2.42086870	0.32847933
C	-4.32990328	-0.08415248	0.26397034
O	3.17424044	2.43879734	-0.54997712
O	0.21457806	4.32717470	1.94664405
C	-0.66665182	2.12058564	3.37060660
C	-1.92455239	2.92386352	3.37215087
C	-3.15613510	2.41509971	3.51838710
C	-4.36037944	3.29525657	3.47434814
C	-3.43145271	0.96389331	3.72180397
C	-2.61091242	-2.63453788	1.65053205
C	-1.13467395	-2.50753778	1.45672852
C	-0.36235951	-3.42316119	0.85606119
C	1.10434622	-3.20469232	0.67655841
C	-0.87087310	-4.70987568	0.30047266
C	1.15454873	1.29365841	-2.80298829
C	1.67436160	1.40351290	-4.20103035
C	2.85477534	1.95278500	-4.51946354
C	3.33304712	2.00467929	-5.93285310
C	3.77728036	2.54559891	-3.51129145
O	3.39892834	-1.86029210	-3.48981017
O	5.92854777	-0.89772210	-3.79690155
O	3.19009737	-0.77040849	1.86007042
C	1.54062281	4.53758797	-0.47306605
C	2.51110061	5.12735338	0.49756898
C	3.15472817	6.28149562	0.26833599
C	4.11327407	6.84507154	1.26186399
C	2.98679649	7.06564247	-0.98831857
H	6.90105790	0.46779211	-1.67593908
H	5.62291906	0.79653743	0.43150082
H	2.23421894	-1.28618135	-1.27649686
H	-1.49152386	0.39202679	1.33856685
H	-2.08173211	2.01999100	1.04594680
H	0.15190704	0.55799675	-0.52484384
H	1.85704491	-0.52823590	2.85595128
H	-1.65225512	3.94305526	0.00907399
H	-0.98320169	4.64554612	-1.46880542
H	-2.15918474	3.34822291	-1.56714540
H	-0.53949757	2.58574774	-3.13340744
H	0.93164533	3.44538985	-2.71731032
H	-2.73069835	1.41113101	-1.25428266
H	-1.57398338	0.74082366	-2.39538993
H	-1.40142123	-1.37983925	-1.13288426
H	-4.44440209	-2.68207142	0.47352836
H	-3.00269490	-3.13210415	-0.43531621
H	-4.04410361	0.97577213	0.23503944
H	-5.20126786	-0.20020983	-0.39942427
H	-4.68031306	-0.29059393	1.28593808
H	-0.83140261	1.11921544	3.82650861
H	0.09574483	2.61103083	4.01716632
H	-1.77011384	3.99407805	3.23632564
H	-4.99763331	3.04803857	2.61076744
H	-4.11487244	4.36296435	3.39694362
H	-4.98439802	3.17285363	4.37203627
H	-3.05365217	0.35807922	2.88417306
H	-4.50291014	0.73989419	3.81457010
H	-2.94748729	0.58255407	4.63464094

H	-2.97063840	-1.90742075	2.40604607
H	-2.86731756	-3.63354412	2.06244672
H	-0.70958403	-1.57906481	1.83379293
H	1.44182511	-2.22854145	1.04521230
H	1.38613599	-3.27446484	-0.38522094
H	1.69060664	-3.96760133	1.21117622
H	-1.78007809	-5.06163924	0.80771244
H	-1.12436009	-4.59982943	-0.76593988
H	-0.12969513	-5.51777991	0.37174795
H	1.99420895	1.15612447	-2.09334175
H	0.53155001	0.38113236	-2.70529214
H	1.01805797	0.99162283	-4.96226097
H	4.27857397	1.45254592	-6.04702849
H	3.52645192	3.03850526	-6.25383692
H	2.62053813	1.57034170	-6.64564248
H	3.27918049	3.33215585	-2.92395189
H	4.68106147	2.97923985	-3.95417324
H	4.10709241	1.79083656	-2.77544182
H	3.92677919	-1.81994501	-4.33795540
H	6.76715101	-0.36306941	-3.90684389
H	2.03958819	4.41516343	-1.46443120
H	0.70905957	5.25237236	-0.65582320
H	2.67720167	4.56643262	1.41363691
H	4.15173145	6.26920384	2.19668776
H	3.85928016	7.88089512	1.53060430
H	5.13672065	6.86008452	0.85522346
H	1.93115134	7.17933004	-1.27398115
H	3.41124822	8.07644750	-0.92051178
H	3.49069922	6.56585193	-1.83142619

Energy = -2617.17102767 a.u. #Cluster: 10

O	3.97743664	-3.16619449	-4.83488173
N	3.77279656	-1.02720300	-5.48036754
C	4.30248226	-2.27823835	-5.63408310
C	5.18814751	-2.53011108	-6.78486307
C	5.98751015	-3.59772665	-6.80773511
H	5.15275508	-1.80129210	-7.59713946
H	4.07056757	-0.24419571	-6.03611776
H	3.23695000	-0.79451053	-4.65519346
H	6.65958790	-3.82137867	-7.62922287
H	6.03605956	-4.32952341	-6.00655525
O	-2.83019904	8.40398901	0.69131102
N	-1.04077387	7.08564475	0.39685547
C	-2.16563968	7.44298739	1.08283832
C	-2.51273544	6.65675033	2.28598212
C	-3.77463916	6.55877583	2.70261621
H	-1.67843305	6.16777658	2.79764423
H	-0.49987060	6.27375573	0.66225507
H	-0.76452264	7.56604744	-0.44331881
H	-4.07021727	5.99662214	3.58150637
H	-4.61281591	7.03181218	2.20119584
C	5.03629538	-0.95141110	-2.17109621
C	5.73453661	-0.03954234	-1.39103358
C	5.14719508	0.44837961	-0.22009265
C	3.87717762	0.02050330	0.14044598
C	3.15933174	-0.89221764	-0.63988771
C	3.74491960	-1.38889225	-1.79846653
C	3.21313767	0.51270528	1.36979286
C	2.13286690	1.47402844	1.22736170
C	1.06090981	1.43818361	2.08106168
C	-0.22099774	2.16618084	1.79684861
C	0.15172793	3.48836231	1.12593903
C	0.98973670	3.34127509	-0.13197123
C	2.21674640	2.46781649	0.15594321
C	0.03748145	2.65000211	-1.18578984
C	-1.01737584	1.34983352	0.76089569
C	-0.36328482	1.25674646	-0.62872603
O	1.02845038	0.71643709	3.18394640
C	-1.21165210	3.53722249	-1.35636973
C	0.68987575	2.53957709	-2.58269060
C	-1.35039516	0.51303332	-1.55617250
C	-1.52765653	-0.88443636	-1.06243418
C	-2.63825295	-1.35537954	-0.47927329
C	-2.67971835	-2.76050699	0.04504970
C	-3.88063749	-0.55247677	-0.28922028
O	3.24350214	2.63579508	-0.45578845
O	-0.23170787	4.54020902	1.56358729
C	-1.04375805	2.38269709	3.07960895
C	-2.39991058	2.92451697	2.76939314
C	-3.53663876	2.21682626	2.82807929
C	-4.84620675	2.83694108	2.47048046
C	-3.59845998	0.78369822	3.23648372
C	-2.39745821	-2.78134389	1.56107324
C	-0.98951789	-2.38999472	1.87354988
C	0.08570538	-3.15826052	1.65101036
C	1.45867730	-2.69344193	2.01251694
C	0.03330498	-4.51514898	1.03597224
C	1.63776941	1.35471562	-2.83061072
C	2.25500708	1.51989367	-4.18335421
C	3.43394259	2.12063067	-4.40038759
C	3.97320605	2.30686125	-5.78030556
C	4.28350111	2.66635511	-3.30689146
O	3.01770992	-2.27302195	-2.50609812
O	5.52968511	-1.48022029	-3.31848047
O	3.48234350	0.02488670	2.46089312
C	1.50791065	4.71434565	-0.59934744
C	2.29485815	5.41381873	0.46106033
C	3.58068915	5.77664861	0.35061543
C	4.27499102	6.47536008	1.47394435
C	4.42233468	5.52174551	-0.85181016
H	6.72726449	0.29656288	-1.69097290
H	5.68135605	1.17707176	0.39192292
H	2.15472826	-1.21547269	-0.36534862
H	-1.19383034	0.32421391	1.15227212
H	-2.03360870	1.78870704	0.65952008
H	0.56370907	0.63379709	-0.55142032
H	1.93301111	0.24879424	3.36815974
H	-1.75777514	3.69325392	-0.42058436
H	-0.95878266	4.52481169	-1.76026636
H	-1.92536175	3.08881083	-2.06007510
H	-0.11409332	2.49487757	-3.34841902
H	1.23334145	3.47949008	-2.80773104
H	-2.31270935	1.05759399	-1.61249335
H	-0.96864194	0.50132370	-2.59871986
H	-0.64563545	-1.51515743	-1.17579084
H	-3.66617698	-3.22447533	-0.15479937
H	-1.94184750	-3.40400449	-0.47631037
H	-3.69858432	0.33614310	0.33376514
H	-4.28192290	-0.19312885	-1.24844173
H	-4.68369756	-1.11824697	0.20169396
H	-1.11318639	1.43064306	3.64932738
H	-0.50272602	3.08559858	3.75292396
H	-2.40284751	3.97180912	2.46580096
H	-5.28517708	2.35088275	1.58539843

H	-4.76879892	3.90816699	2.24006579
H	-5.57840117	2.73324495	3.28464562
H	-2.90057122	0.16066791	2.65727441
H	-4.59657663	0.34451653	3.10472827
H	-3.33313415	0.65734857	4.29792291
H	-3.11350001	-2.10927763	2.07606808
H	-2.61537682	-3.79584101	1.95730675
H	-0.88412750	-1.39871551	2.31049926
H	1.49764512	-1.62836388	2.27023264
H	2.17197137	-2.86198481	1.19178396
H	1.84433303	-3.24725963	2.88280131
H	-0.93488139	-5.01272795	1.18498310
H	0.19691445	-4.45443814	-0.05211267
H	0.80469427	-5.18561691	1.43931830
H	2.41623514	1.29174687	-2.04509451
H	1.08965470	0.39218382	-2.77683288
H	1.64971430	1.14419206	-5.00475011
H	4.98347153	1.88330105	-5.88430933
H	4.06568060	3.37770318	-6.02234290
H	3.34056720	1.86590023	-6.56174969
H	3.74221425	3.43121100	-2.72490745
H	5.21447085	3.12020004	-3.66341999
H	4.55781517	1.88717082	-2.57465885
H	3.47724724	-2.59035770	-3.35863466
H	6.39953764	-1.06281336	-3.57819138
H	2.12350050	4.56945600	-1.51691015
H	0.66114119	5.35518433	-0.92242284
H	1.74030916	5.63522215	1.37195855
H	3.62999453	6.63607115	2.34812509
H	4.64908025	7.46181944	1.16283766
H	5.14690699	5.89917729	1.81915499
H	3.94764128	5.88553322	-1.77398613
H	5.41186908	5.99143605	-0.79383635
H	4.58962895	4.43690549	-0.98339536

OC-(AM)$_3$ complex in the DMSO

Energy = -2864.31633933 a.u. #Cluster: 1

O	5.69461325	-0.86587626	-4.38940979
N	4.90749701	-1.63318248	-6.33039621
C	5.94359639	-1.39152147	-5.48987109
C	7.31973542	-1.69645090	-5.91493106
C	8.36230640	-1.24659371	-5.21400754
H	7.43031922	-2.29742987	-6.81976022
H	5.00807629	-2.14252989	-7.19579118
H	3.95903127	-1.43743337	-6.04583441
H	9.39354563	-1.44250372	-5.48907595
H	8.26829722	-0.64487479	-4.31380248
O	1.78247565	-0.56146057	4.59879041
N	3.61312926	-1.57217630	3.78668256
C	2.41759354	-1.60983511	4.44105829
C	1.94827689	-2.89900682	4.98627826
C	0.64452729	-3.17237809	5.03986137
H	2.71883416	-3.59199407	5.32652700
H	4.13105256	-2.40200127	3.55365379
H	3.92023222	-0.70520993	3.36178990
H	0.24397209	-4.10263795	5.42581878
H	-0.12493027	-2.48818872	4.69259930
O	7.66584099	3.39045473	-0.73906934
N	5.60390999	3.22164239	0.11941513
C	6.50582024	2.99733086	-0.88036451
C	6.03326502	2.26969786	-2.07607448
C	6.87507106	1.93058697	-3.05241606
H	4.96787859	2.01791756	-2.09982788
H	4.63599484	2.94904071	0.02521797
H	5.85582264	3.72904263	0.94866273
H	6.57097773	1.38235254	-3.93879077
H	7.93371308	2.16630460	-3.04172335
C	5.67953682	-0.91574112	-1.12275473
C	6.14911024	-0.44553391	0.10333801
C	5.26072755	0.18434786	0.96995492
C	3.91913618	0.34568743	0.61515920
C	3.43070180	-0.17770703	-0.58544641
C	4.31158530	-0.80848144	-1.45353113
C	3.02102575	1.05249196	1.54486704
C	1.83590616	1.76442367	1.08734537
C	0.72770415	1.79868948	1.90045797
C	-0.55224277	2.49242327	1.49781664
C	-0.16124743	3.67264468	0.60903823
C	0.62987474	3.27656897	-0.62747074
C	1.86711273	2.48176235	-0.18439621
C	-0.37417441	2.43522657	-1.50358406
C	-1.36901179	1.49806240	0.65564066
C	-0.73278021	1.15487669	-0.70296429
O	0.60636134	1.21380541	3.06059628
C	-1.64050426	3.28571393	-1.74052472
C	0.17001963	2.10784473	-2.91103526
C	-1.70422450	0.21890045	-1.45852815
C	-1.73620681	-1.09132979	-0.74425660
C	-2.69781930	-1.50636666	0.09081349
C	-2.51591777	-2.77562041	0.87467956
C	-3.97534491	-0.78646150	0.35062230
O	2.86741147	2.50898503	-0.86644613
O	-0.46760115	4.80282014	0.87475302
C	-1.34492944	2.95786633	2.73302557
C	-2.70010164	3.45662317	2.35324130
C	-3.84557242	2.81016525	2.61227691
C	-5.15981660	3.36909383	2.18056337
C	-3.91284371	1.50598076	3.33368028
C	-1.62290459	-2.51403486	2.10771309
C	-0.21760264	-2.22292056	1.69086400
C	0.71355397	-3.15935143	1.46421374
C	2.08304420	-2.79315302	0.99683082
C	0.47048228	-4.61916797	1.64922939
C	1.20861511	0.98408685	-3.02972871
C	1.42532216	0.69946497	-4.48261776
C	2.45245647	1.15995862	-5.20829376
C	2.55296505	0.86580126	-6.67066546
C	3.56363011	1.98591483	-4.65742344
O	3.78915762	-1.35535973	-2.57933617
O	6.58001286	-1.49579098	-1.94585171
O	3.26175823	1.01300231	2.74699476
C	1.13582297	4.53278962	-1.35930082
C	2.09698028	5.32448525	-0.53322595
C	3.32204817	5.68805069	-0.93818914
C	4.20875475	6.51059068	-0.06246082
C	3.89997248	5.31216773	-2.26071213
H	7.20091163	-0.55999957	0.35970873
H	5.62336296	0.57427297	1.92542910
H	2.37611223	-0.09918150	-0.85389423
H	-1.53135881	0.56419429	1.23717565
H	-2.39230033	1.90043411	0.49803969
H	0.21209960	0.58090132	-0.52650885
H	1.41036630	0.65065076	3.41371527

H	-2.12768621	3.60927933	-0.81536244
H	-1.42068411	4.18825555	-2.32329265
H	-2.39536370	2.72294724	-2.30457973
H	-0.69135822	1.84879772	-3.56519062
H	0.59101300	3.02641959	-3.36818054
H	-2.70655303	0.67978519	-1.54191780
H	-1.37125616	0.07368181	-2.50680670
H	-0.84337936	-1.70081857	-0.89936302
H	-3.48898010	-3.18828375	1.20339474
H	-2.05994593	-3.56732792	0.24729237
H	-4.01172242	0.21531950	-0.09774315
H	-4.83210789	-1.34813992	-0.05247761
H	-4.15816970	-0.66253787	1.42854577
H	-1.41714064	2.12887277	3.47121780
H	-0.77958858	3.76190270	3.25546944
H	-2.69673171	4.40757937	1.82267042
H	-5.63699621	2.72072140	1.42929383
H	-5.07940200	4.36830560	1.73191303
H	-5.86340728	3.44840778	3.02213818
H	-3.19963820	0.77286100	2.92818295
H	-4.90720743	1.04240920	3.28311861
H	-3.67458417	1.62809271	4.40220779
H	-2.03815975	-1.67057756	2.69552068
H	-1.66596256	-3.39208418	2.78343650
H	0.00019771	-1.16563593	1.55326647
H	2.32522621	-1.73783541	1.17545982
H	2.18445389	-2.97082569	-0.08575104
H	2.86029700	-3.39294488	1.49073904
H	0.46202129	-4.88225817	2.71836700
H	-0.49890595	-4.93399575	1.23739662
H	1.23724606	-5.24261608	1.17154884
H	2.15896662	1.27089126	-2.53556551
H	0.86557979	0.06248787	-2.51823970
H	0.65116920	0.08472729	-4.93725795
H	3.59309915	0.73169962	-6.99840111
H	2.14452070	1.69790744	-7.26439988
H	2.00056048	-0.03619281	-6.96504715
H	3.27947316	2.51987595	-3.73771010
H	3.91918286	2.73720833	-5.37450233
H	4.42430811	1.34030641	-4.41026478
H	4.37779086	-1.17729578	-3.38009766
H	6.34377763	-1.34026306	-2.92156343
H	1.60131180	4.22292294	-2.32179733
H	0.27549884	5.17956040	-1.63712912
H	1.72063497	5.61933164	0.44429920
H	3.76065517	6.73771830	0.91415256
H	4.45551988	7.47225338	-0.53598189
H	5.16292913	5.99876785	0.13251753
H	3.29596578	5.69563398	-3.09500932
H	4.92053178	5.68707436	-2.40694052
H	3.94051017	4.21404395	-2.36971587

Energy = -2864.31412389 a.u. #Cluster: 2

O	7.66384226	-2.57399849	-3.64451079
N	6.22497483	-3.48401558	-5.12107540
C	7.41458879	-3.47412282	-4.46804602
C	8.39527931	-4.54869952	-4.71990570
C	8.74072283	-4.94551893	-5.94311276
H	8.83402020	-4.96933693	-3.80955966
H	5.98467402	-4.19120460	-5.80058232
H	5.53682991	-2.75400601	-4.98236331
H	9.47730567	-5.72067007	-6.12977288
H	8.33250543	-4.53579575	-6.86037543
O	3.57702780	-1.99472127	-5.48467476
N	4.61265004	-0.03056997	-5.74722651
C	3.69173880	-0.95114381	-6.14326500
C	2.88961762	-0.68397566	-7.35259189
C	1.57506399	-0.90044976	-7.35980874
H	3.44140537	-0.31310382	-8.21919675
H	4.72518378	0.86012018	-6.19918075
H	5.13142374	-0.18602977	-4.89358729
H	0.94651078	-0.71930694	-8.22510923
H	1.01931825	-1.27261860	-6.50484502
O	7.65849342	3.64468481	-1.13065055
N	5.80244439	3.27062205	0.06654862
C	6.49262648	3.24407134	-1.11108415
C	5.79752924	2.70549555	-2.29928035
C	6.36108069	2.75230711	-3.50614664
H	4.80872365	2.26754612	-2.12553418
H	4.82883700	3.00707158	0.11037105
H	6.20512813	3.65317341	0.90350827
H	5.88612111	2.36986086	-4.40078925
H	7.34119534	3.17660733	-3.69622459
C	4.99393416	-0.82826018	-2.14525321
C	5.77063750	-0.34091260	-1.10372384
C	5.13597923	0.15244648	0.04037670
C	3.74880733	0.16764622	0.11202590
C	2.95835746	-0.37141987	-0.91168761
C	3.58273102	-0.88682607	-2.03731966
C	3.05329613	0.71422961	1.29392543
C	1.92334029	1.61453482	1.09038035
C	0.83882688	1.53719949	1.92750068
C	-0.47029933	2.20703384	1.61745414
C	-0.11940553	3.54912581	0.96979068
C	0.71543029	3.42501467	-0.29740218
C	1.96532587	2.58503120	-0.00066419
C	-0.24063413	2.73867884	-1.34474052
C	-1.21126502	1.35057888	0.57376417
C	-0.55474122	1.31318943	-0.81756825
O	0.81818426	0.82969695	3.03864722
C	-1.53495451	3.57471781	-1.43565890
C	0.33340880	2.71340560	-2.77802371
C	-1.48494057	0.52054712	-1.76133089
C	-1.60751356	-0.88101082	-1.26271199
C	-2.69357409	-1.40547547	-0.67994209
C	-2.66625380	-2.81546760	-0.16442115
C	-3.98510243	-0.68613617	-0.49168258
O	2.98267964	2.78128955	-0.62623798
O	-0.48764718	4.59475279	1.42966715
C	-1.32237812	2.38742459	2.88617527
C	-2.68174392	2.91219706	2.56055547
C	-3.80446373	2.17969275	2.58404779
C	-5.12094657	2.77741120	2.21560123
C	-3.84032011	0.73491465	2.95388397
C	-2.27149691	-2.84696735	1.32609555
C	-0.87348909	-2.37049700	1.55323515
C	0.23285210	-3.04638407	1.21421962
C	1.59327740	-2.49497682	1.49110279
C	0.22984619	-4.37330433	0.53528113
C	1.44765326	1.70526176	-3.09028105
C	1.80438119	1.81150870	-4.53998112
C	2.56357730	2.78307833	-5.06422237
C	2.84613450	2.84533048	-6.53005235
C	3.15563595	3.89426150	-4.26634503

1	O	2.79680500	-1.45003969	-2.97471988
2	O	5.49379056	-1.22448797	-3.32705399
3	O	3.35004785	0.33865749	2.42176211
4	C	1.19978312	4.81607265	-0.74452359
5	C	2.11434339	5.43670242	0.26178896
6	C	3.32829570	5.93275306	-0.01500289
7	C	4.17442151	6.53680979	1.05704682
8	C	3.93912066	5.91813090	-1.37511623
9	H	6.85668098	-0.33218770	-1.18346605
10	H	5.73575145	0.53785048	0.86723739
11	H	1.87080516	-0.40207471	-0.83702353
12	H	-1.32245755	0.31208887	0.95592953
13	H	-2.25524670	1.71973899	0.47709204
14	H	0.40954308	0.74788850	-0.74976414
15	H	1.74489250	0.43098738	3.26507547
16	H	-2.06662107	3.65760062	-0.48243782
17	H	-1.33538023	4.59468523	-1.78538298
18	H	-2.24540689	3.13205842	-2.14573352
19	H	-0.50310113	2.52212382	-3.48526548
20	H	0.68814848	3.72997350	-3.04468258
21	H	-2.46823440	1.02067396	-1.84880417
22	H	-1.07421590	0.51689280	-2.79331015
23	H	-0.69724188	-1.47293864	-1.37099163
24	H	-3.65486151	-3.29908790	-0.29430802
25	H	-1.95718542	-3.43747623	-0.74759757
26	H	-3.86468228	0.40359165	-0.42328836
27	H	-4.66885905	-0.88158493	-1.33182779
28	H	-4.50715987	-1.00387539	0.42249484
29	H	-1.38704342	1.42222337	3.43545046
30	H	-0.80607176	3.08418649	3.58408141
31	H	-2.70043600	3.96407863	2.27854656
32	H	-5.54545760	2.28227556	1.32830967
33	H	-5.06014334	3.84898275	1.98274037
34	H	-5.85753433	2.66424185	3.02469719
35	H	-3.23366685	0.12724428	2.26463447
36	H	-4.85452991	0.31369292	2.93425766
37	H	-3.44488198	0.56278863	3.96685471
38	H	-2.99042056	-2.23490842	1.90775426
39	H	-2.39408525	-3.88202562	1.71060691
40	H	-0.80448282	-1.39557601	2.03293487
41	H	1.56868758	-1.49512772	1.94045274
42	H	2.19048691	-2.42876218	0.56817819
43	H	2.15166406	-3.14303311	2.18378868
44	H	1.08313427	-4.99792561	0.83332495
45	H	-0.68021017	-4.95364192	0.74053218
46	H	0.28931394	-4.24998076	-0.55830364
47	H	2.34270194	1.88723743	-2.46206225
48	H	1.12860895	0.67003256	-2.85299641
49	H	1.38698136	1.02591660	-5.16494792
50	H	3.92157551	2.94030560	-6.73912052
51	H	2.36501939	3.72492812	-6.98445869
52	H	2.48014966	1.96836398	-7.07946067
53	H	3.26792000	3.62828866	-3.20162532
54	H	2.51719908	4.78952671	-4.30807679
55	H	4.14649992	4.19163899	-4.63540586
56	H	3.29581542	-1.66134856	-3.82645472
57	H	6.43945664	-1.63417874	-3.29713458
58	H	1.70089358	4.72129853	-1.73335707
59	H	0.32832229	5.48541308	-0.91158484
60	H	1.71506989	5.47751548	1.27354655
61	H	3.70090447	6.50959810	2.04753025
62	H	4.40615444	7.58976260	0.83938981
63	H	5.13659752	6.01014679	1.14672324
64	H	3.28959834	6.39270618	-2.12397211
65	H	4.90579164	6.43591091	-1.41805549
66	H	4.11323262	4.88033157	-1.70870392
67				
68	Energy = -2864.31254030 a.u. #Cluster: 3			
69	O	3.62575018	-2.73807806	-4.18681940
70	N	2.06781348	-1.81652203	-5.49018109
71	C	3.36379098	-2.10769753	-5.23370033
72	C	4.40329317	-1.72865564	-6.20311102
73	C	5.68420029	-1.62957696	-5.84390731
74	H	4.06323616	-1.53574153	-7.22481763
75	H	1.77579882	-1.26476714	-6.28340022
76	H	1.33736609	-2.05840899	-4.83491367
77	H	6.47501326	-1.35536656	-6.53634848
78	H	6.05085280	-1.81074764	-4.83828035
79	O	2.23152848	-0.77047821	4.52507493
80	N	4.08629578	-1.60675728	3.58532497
81	C	2.91439242	-1.76783423	4.25991120
82	C	2.52238152	-3.12111118	4.69615952
83	C	1.23498594	-3.42974576	4.85914450
84	H	3.33150626	-3.83213858	4.86833482
85	H	4.65619713	-2.38019114	3.28451637
86	H	4.36563602	-0.68766526	3.26958168
87	H	0.89026302	-4.40771617	5.17496909
88	H	0.42366188	-2.72982000	4.67908998
89	O	-2.77662760	8.76768696	0.42784848
90	N	-1.02236420	7.39357123	0.17731498
91	C	-2.17045086	7.76678974	0.81438417
92	C	-2.61331968	6.94765778	1.96318209
93	C	-3.90023162	6.87677603	2.30104200
94	H	-1.82711518	6.41007718	2.50217053
95	H	-0.52855652	6.54977172	0.43722611
96	H	-0.68375529	7.89326170	-0.62781720
97	H	-4.26620278	6.29024954	3.13622788
98	H	-4.69151636	7.39739241	1.77202399
99	C	4.90035108	-1.27643642	-1.41556025
100	C	5.76800678	-0.51885678	-0.62914235
101	C	5.25309839	0.24990895	0.41271820
102	C	3.88113795	0.26966771	0.65207056
103	C	3.00533369	-0.50169158	-0.11580208
104	C	3.51418449	-1.28222452	-1.14796460
105	C	3.33213175	1.13732951	1.71567310
106	C	2.11315582	1.88683291	1.40484402
107	C	0.97380900	1.77210596	2.14205802
108	C	-0.31206360	2.46310493	1.75332961
109	C	0.05163692	3.74397229	1.00692358
110	C	0.95094975	3.53601279	-0.19963754
111	C	2.20591644	2.78183031	0.25013970
112	C	0.09599146	2.70668165	-1.23579899
113	C	-1.04946609	1.55690242	0.75214160
114	C	-0.29904501	1.35778922	-0.57557149
115	O	0.81298011	1.02121360	3.20669871
116	C	-1.16707933	3.52382150	-1.57236700
117	C	0.84442642	2.48514065	-2.57058034
118	C	-1.18104246	0.48023208	-1.49244940
119	C	-1.30565943	-0.87721612	-0.88355794
120	C	-2.37778733	-1.33872162	-0.22673796
121	C	-2.33587404	-2.68123440	0.44647187
122	C	-3.66548902	-0.60491125	-0.07198104
123	O	3.26395228	2.96092483	-0.30569130
124	O	-0.36950542	4.81588150	1.35205051

C	-1.18228932	2.75550344	2.98902280
C	-2.54337946	3.22601169	2.59505007
C	-3.66496622	2.50313593	2.71914687
C	-4.98287509	3.04765110	2.27878330
C	-3.70083198	1.12470887	3.28833045
C	-1.60446750	-2.57890726	1.80266727
C	-0.12604827	-2.49898213	1.60020611
C	0.67209672	-3.56727671	1.46664829
C	2.13689123	-3.41922147	1.22291958
C	0.17694474	-4.97314301	1.53847842
C	1.86444679	1.33805011	-2.61840053
C	2.54484761	1.34680485	-3.94940854
C	3.82501992	1.68862116	-4.14694922
C	4.42685718	1.67693953	-5.51404657
C	4.74869126	2.10137080	-3.05263809
O	2.61139307	-2.03742837	-1.81325502
O	5.47443267	-2.00009699	-2.40019463
O	3.86104824	1.20620287	2.80939566
C	1.41099510	4.89094841	-0.76888606
C	2.19100697	5.68653821	0.22690279
C	3.44497316	6.12067228	0.03604549
C	4.14936089	6.91580761	1.08574966
C	4.23434460	5.85813907	-1.20113535
H	6.83652769	-0.53106359	-0.84130815
H	5.92700378	0.84869961	1.02970863
H	1.92946527	-0.50908102	0.07447033
H	-1.24389375	0.56792766	1.22161715
H	-2.06083260	1.96957777	0.55355662
H	0.64355993	0.78722035	-0.37365182
H	1.61137882	0.44926936	3.52405649
H	-1.75478133	3.78796893	-0.68738886
H	-0.92368096	4.45484659	-2.09818872
H	-1.83809464	2.96310131	-2.23567870
H	0.09641402	2.31211636	-3.37320371
H	1.35586474	3.42353840	-2.86642392
H	-2.16629419	0.95675068	-1.65724430
H	-0.73291555	0.40217720	-2.50474020
H	-0.40352052	-1.48720340	-0.96797252
H	-3.35605392	-3.08063342	0.60487005
H	-1.82754549	-3.42864584	-0.19543589
H	-3.63134026	0.42392295	-0.45442187
H	-4.47925230	-1.11740873	-0.60701256
H	-3.96886268	-0.54245215	0.98374050
H	-1.24284398	1.85046399	3.63193188
H	-0.68509177	3.52761681	3.61861894
H	-2.56485001	4.23072260	2.17214837
H	-5.39044699	2.46320419	1.43936444
H	-4.92895111	4.09183435	1.94239452
H	-5.72896134	3.00518648	3.08540546
H	-2.93637655	0.47393352	2.83792238
H	-4.66853550	0.62732140	3.13936754
H	-3.51340071	1.13535560	4.37352950
H	-1.97103719	-1.69375081	2.35992369
H	-1.87260726	-3.45158287	2.43192200
H	0.26667102	-1.48675124	1.54288020
H	2.51848876	-2.43108611	1.50951541
H	2.36360899	-3.54611359	0.15057773
H	2.72509538	-4.16806522	1.76970881
H	-0.03031515	-5.26306389	2.57971185
H	-0.75807717	-5.11198226	0.97715343
H	0.89859544	-5.69810493	1.14030179
H	2.59895368	1.42540727	-1.79480633
H	1.36977251	0.35518324	-2.46487881
H	1.90585963	1.05325656	-4.77923266
H	5.32142485	1.03772531	-5.55075224
H	4.74249250	2.68553724	-5.81844715
H	3.73838792	1.30966754	-6.28584351
H	4.27759112	2.84014602	-2.38197929
H	5.68186747	2.54181712	-3.42175917
H	5.02128877	1.24173214	-2.41983295
H	2.92486725	-2.31228272	-2.72841041
H	4.81841076	-2.30230078	-3.10732604
H	2.01458901	4.70457709	-1.68674494
H	0.53559029	5.47806834	-1.11679434
H	1.66288649	5.90973270	1.15243794
H	3.52160307	7.12631084	1.96201263
H	4.49294967	7.88424965	0.69391812
H	5.04217129	6.38460636	1.44932555
H	3.77764433	6.33254907	-2.08168060
H	5.26708147	6.22267803	-1.13895981
H	4.28747232	4.77581981	-1.41285107

Energy = -2864.31167291 a.u. #Cluster: 4

O	5.51919910	-3.52407657	-4.01347691
N	3.38613524	-4.12030225	-3.62486151
C	4.52410290	-4.15220552	-4.38461036
C	4.54420871	-5.02472125	-5.57647690
C	5.20319653	-4.65039332	-6.67274664
H	3.99141224	-5.96199589	-5.49976938
H	2.52165745	-4.52764227	-3.94651864
H	3.29998514	-3.43525977	-2.88658254
H	5.24585018	-5.24520534	-7.57741454
H	5.74570225	-3.71254748	-6.75171950
O	3.94955090	-1.57920656	-5.32008483
N	1.74767476	-1.25679361	-5.60259176
C	3.01822855	-1.32540777	-6.09841424
C	3.23724438	-1.13464309	-7.54243831
C	4.47714882	-1.02726425	-8.02355825
H	2.34837813	-1.08722430	-8.17270477
H	0.95497842	-0.98249886	-6.15710597
H	1.59156117	-1.34608541	-4.61024513
H	4.69965745	-0.88483453	-9.07482932
H	5.36409838	-1.07476094	-7.39649614
O	7.38103982	4.58368148	0.95060569
N	5.25027846	3.92537843	1.17270557
C	6.37504118	4.11111255	0.41887882
C	6.32276976	3.69447974	-0.99727317
C	7.27531032	4.05440794	-1.85704805
H	5.46135695	3.08316140	-1.28803165
H	4.38698607	3.61472694	0.75274588
H	5.20242038	4.23731246	2.12787190
H	7.27250350	3.77026628	-2.90360449
H	8.13202124	4.66059707	-1.58475296
C	5.30541580	-0.57255348	-2.04535810
C	6.00300147	0.14873183	-1.07888108
C	5.30749500	0.69021308	0.00306303
C	3.92965701	0.53175890	0.09511210
C	3.21558098	-0.17930598	-0.87496027
C	3.90540468	-0.73077480	-1.94575504
C	3.16551291	1.05152804	1.24730471
C	1.97445484	1.85490487	0.99720780
C	0.86929540	1.69134210	1.79404350
C	-0.48167840	2.23337828	1.42150821
C	-0.23823365	3.57724932	0.72897059

C	0.65502994	3.48984537	-0.50070060
C	1.97148797	2.80783511	-0.11069547
C	-0.17548656	2.65894167	-1.55256888
C	-1.10383160	1.27325141	0.38956973
C	-0.37812547	1.23661186	-0.96710503
O	0.86708382	0.99580435	2.91269204
C	-1.53865527	3.35215116	-1.75311537
C	0.48631365	2.62133709	-2.94799155
C	-1.17602057	0.30639451	-1.90630209
C	-1.06944858	-1.09315535	-1.39789555
C	-2.04783176	-1.77307597	-0.78652200
C	-1.78863194	-3.15136885	-0.25060526
C	-3.43366335	-1.26484063	-0.57957303
O	3.00406114	3.10966796	-0.66659243
O	-0.72673384	4.59765003	1.12960184
C	-1.38745468	2.38073232	2.65647491
C	-2.78067346	2.75374789	2.27071641
C	-3.82745653	1.91664019	2.30278308
C	-5.18471704	2.36527736	1.87516974
C	-3.73430771	0.49412526	2.74240975
C	-1.34800847	-3.08358565	1.22579828
C	0.02629776	-2.51612406	1.37767321
C	1.15756164	-3.16937216	1.07828453
C	2.49756942	-2.54472619	1.28805743
C	1.19825668	-4.54602891	0.50668596
C	1.67123320	1.66675649	-3.13434298
C	2.20133260	1.80878717	-4.52464707
C	3.45077981	2.18074227	-4.83179609
C	3.90750249	2.25246113	-6.25266289
C	4.48940237	2.52053996	-3.81789567
O	3.18604501	-1.44505247	-2.82992883
O	6.02010581	-1.06671178	-3.07510445
O	3.45707241	0.71308607	2.38767068
C	0.99815786	4.90227517	-1.00734865
C	1.79218654	5.68826119	-0.01509326
C	2.98302026	6.24827240	-0.27101091
C	3.69112017	7.05584870	0.76682172
C	3.69407282	6.12000024	-1.57564689
H	7.08112114	0.27449466	-1.17125423
H	5.85081692	1.23491719	0.77722645
H	2.13553764	-0.31864052	-0.80617603
H	-1.14552040	0.24592483	0.81217476
H	-2.16843663	1.54995903	0.23227805
H	0.63273374	0.77497812	-0.82561447
H	1.81367218	0.67440519	3.17465464
H	-2.11217640	3.45841182	-0.82701067
H	-1.42597473	4.35478841	-2.18294691
H	-2.17361615	2.78334567	-2.44475089
H	-0.28983591	2.35957869	-3.69924584
H	0.80665455	3.64495481	-3.23091358
H	-2.22649518	0.64381949	-1.99132186
H	-0.77962596	0.36167629	-2.94152632
H	-0.08211065	-1.53974320	-1.52566607
H	-2.69443071	-3.78304497	-0.33918050
H	-1.01117028	-3.67097312	-0.84653633
H	-3.49372117	-0.16826250	-0.56822176
H	-4.10085542	-1.61355421	-1.38283492
H	-3.86668002	-1.61794959	0.36763274
H	-1.37541941	1.43740670	3.24625782
H	-0.96445989	3.15418970	3.33637872
H	-2.89266206	3.78422199	1.93601466
H	-5.53727192	1.78620467	1.00750139
H	-5.21961398	3.42424103	1.58599290
H	-5.92780943	2.22345070	2.67382355
H	-3.05341455	-0.08331274	2.09856160
H	-4.70244816	-0.02422472	2.72069002
H	-3.35139925	0.40850440	3.77117571
H	-2.08268580	-2.48422634	1.80099894
H	-1.39643606	-4.10292337	1.66449497
H	0.05159865	-1.49791273	1.76004742
H	2.44084230	-1.52582704	1.68995889
H	3.06155410	-2.49443861	0.34308138
H	3.10555398	-3.13312192	1.99163407
H	0.34527922	-5.16178730	0.82364199
H	1.16901764	-4.50885076	-0.59387599
H	2.11054856	-5.09145234	0.78395709
H	2.46006447	1.86263630	-2.38234099
H	1.36641258	0.61179643	-2.96764188
H	1.47955247	1.57095319	-5.30329465
H	4.69378180	1.50605488	-6.44545050
H	4.32766309	3.23762058	-6.49719249
H	3.10310387	2.05676863	-6.97302269
H	4.05242328	3.02451670	-2.93877079
H	5.27089024	3.17749777	-4.21890028
H	4.98401304	1.60488266	-3.45413978
H	3.59091210	-1.45731119	-3.78493457
H	5.60448004	-1.88912956	-3.49951375
H	1.54613364	4.81207675	-1.97244286
H	0.06270189	5.45435689	-1.24457746
H	1.31587032	5.80461637	0.95650913
H	3.14686515	7.10593746	1.71953424
H	3.84640200	8.09096376	0.42862865
H	4.68517281	6.63924632	0.98656851
H	3.11011158	6.54857285	-2.40234441
H	4.67259207	6.61554071	-1.58339184
H	3.86617774	5.05740127	-1.82353464

Energy = -2864.30912391 a.u. #Cluster: 5

O	2.37681153	-1.06116203	3.74919359
N	4.46382934	-1.51701677	3.05401123
C	3.30884235	-1.86863906	3.68351807
C	3.19077525	-3.22714411	4.25217542
C	2.00485368	-3.83454538	4.30096055
H	4.11196291	-3.68336896	4.61645599
H	5.29839563	-2.07866115	3.08410155
H	4.57266690	-0.57731302	2.69625928
H	1.85711878	-4.82845474	4.70644695
H	1.08866652	-3.38369847	3.92609648
O	-2.70075253	9.01648570	0.81544020
N	-0.97220953	7.62247646	0.50552283
C	-2.12196074	7.98072614	1.14818558
C	-2.59980335	7.10169824	2.23700890
C	-3.89099220	7.05057688	2.56189051
H	-1.83535982	6.50596304	2.74424597
H	-0.49815581	6.75542153	0.71997239
H	-0.60760894	8.16540186	-0.25921798
H	-4.28275917	6.42372076	3.35499395
H	-4.66069237	7.63028464	2.06283455
O	7.59658249	3.93263422	1.04622672
N	5.37784541	3.71324074	1.26212108
C	6.51288441	3.70274994	0.50475447
C	6.36931477	3.39880482	-0.93296236
C	7.43381293	3.30697218	-1.72997793
H	5.34667667	3.25444949	-1.29554902

1	H	4.47115990	3.55751524	0.85071127
2	H	5.40134744	3.92364803	2.24607209
3	H	7.36821381	3.08489460	-2.78943013
4	H	8.45389880	3.44381718	-1.38720065
5	C	4.56337038	-1.10170125	-1.99687058
6	C	5.48514253	-0.35663682	-1.27277238
7	C	5.04442750	0.37670207	-0.16992935
8	C	3.69655000	0.37726638	0.17733480
9	C	2.76311574	-0.37688012	-0.54193320
10	C	3.19900390	-1.12153616	-1.62965116
11	C	3.24249108	1.19226159	1.32494603
12	C	2.01143493	1.97055449	1.17016231
13	C	0.95990200	1.83439683	2.02892495
14	C	-0.33997973	2.57954875	1.83831144
15	C	-0.04001852	3.87977147	1.09689869
16	C	0.70290144	3.69870232	-0.21795420
17	C	1.99259309	2.92871322	0.07033642
18	C	-0.27885800	2.91347834	-1.16994731
19	C	-1.22478949	1.73511259	0.90450540
20	C	-0.63381679	1.55548520	-0.50361814
21	O	0.90749496	1.01932045	3.05427776
22	C	-1.55053025	3.76571087	-1.34785456
23	C	0.31776426	2.70859196	-2.58185532
24	C	-1.63654599	0.72648082	-1.33734976
25	C	-1.73009631	-0.64584522	-0.75699060
26	C	-2.73230780	-1.10048775	0.00659835
27	C	-2.65760767	-2.47092689	0.61791408
28	C	-3.96525209	-0.33446868	0.34269396
29	O	2.99975590	3.15877553	-0.56318251
30	O	-0.38917910	4.94663932	1.52604812
31	C	-1.03419126	2.84475669	3.18601468
32	C	-2.43553609	3.32050669	2.98767638
33	C	-3.52962452	2.59092212	3.24637320
34	C	-4.89577265	3.13622886	2.99506601
35	C	-3.48698594	1.20271568	3.79078078
36	C	-1.78730664	-2.44695387	1.89269219
37	C	-0.33608063	-2.35062150	1.54938404
38	C	0.43492663	-3.39958577	1.23132723
39	C	1.87195701	-3.22290359	0.86819998
40	C	-0.05618012	-4.80845650	1.20853158
41	C	1.30873256	1.54876647	-2.74751199
42	C	1.83543287	1.53420584	-4.14637119
43	C	3.08240741	1.88445264	-4.48851673
44	C	3.55043340	1.80980563	-5.90453564
45	C	4.09883177	2.36238120	-3.50846902
46	O	2.26887745	-1.85223403	-2.29068600
47	O	4.88638144	-1.85997761	-3.07071675
48	O	3.86896053	1.21464275	2.36719536
49	C	1.11385243	5.06589400	-0.79767114
50	C	2.03019296	5.81578863	0.11422328
51	C	3.23517543	6.28528291	-0.23956112
52	C	4.07982607	7.03956579	0.73398060
53	C	3.83250391	6.10072686	-1.59305877
54	H	6.53659169	-0.34918359	-1.55965364
55	H	5.76264091	0.95643473	0.41434678
56	H	1.70781576	-0.39594450	-0.26376750
57	H	-1.40313671	0.73878018	1.36495238
58	H	-2.23483864	2.19055625	0.83522771
59	H	0.30901133	0.95700592	-0.42185123
60	H	1.70087758	0.36876840	3.19288701
61	H	-2.02465814	4.02854753	-0.39677107
62	H	-1.34456853	4.70099926	-1.88256013
63	H	-2.30754709	3.23383368	-1.93850238
64	H	-0.51401260	2.55943636	-3.30241011
65	H	0.80957729	3.64493474	-2.91513350
66	H	-2.62137175	1.23131364	-1.37454539
67	H	-1.30705755	0.66940445	-2.39571620
68	H	-0.86315909	-1.27663580	-0.96186914
69	H	-3.66699595	-2.85196962	0.86728374
70	H	-2.24040371	-3.20186880	-0.10381386
71	H	-3.96606515	0.68991639	-0.05328211
72	H	-4.86354586	-0.83125138	-0.05390795
73	H	-4.10178029	-0.25428524	1.43186322
74	H	-1.00828143	1.92767162	3.81417341
75	H	-0.45516451	3.60414116	3.75858290
76	H	-2.51218078	4.33256185	2.59020659
77	H	-5.41945382	2.55003907	2.22400030
78	H	-4.88949561	4.17911393	2.65106701
79	H	-5.51965125	3.09695734	3.89999142
80	H	-2.78919571	0.56221756	3.23102959
81	H	-4.46551507	0.70460861	3.76638589
82	H	-3.15390566	1.19565551	4.84074883
83	H	-2.09293972	-1.59779008	2.53620908
84	H	-1.99050262	-3.35698617	2.49347254
85	H	0.06625720	-1.33977941	1.56304431
86	H	2.29347796	-2.28400475	1.25340479
87	H	1.99739191	-3.20725335	-0.22676529
88	H	2.50007110	-4.03685489	1.25370361
89	H	-0.05074245	-5.23922207	2.22143605
90	H	-1.08617490	-4.88779961	0.83341336
91	H	0.56451600	-5.46022908	0.57963369
92	H	2.13483770	1.62807971	-2.01431524
93	H	0.82222888	0.57283027	-2.53425343
94	H	1.11800107	1.20013712	-4.89200649
95	H	4.42137194	1.14322763	-5.99652585
96	H	3.86298518	2.79633698	-6.27681828
97	H	2.78390233	1.43180826	-6.59303147
98	H	3.66993175	3.10906853	-2.81830976
99	H	4.97428119	2.81739923	-3.98663554
100	H	4.46199950	1.53030233	-2.88345157
101	H	2.65005586	-2.26123727	-3.11904477
102	H	5.83444015	-1.72493038	-3.36059511
103	H	1.59264033	4.90161771	-1.79035946
104	H	0.21322230	5.67792639	-1.01265070
105	H	1.64917708	5.97680065	1.12129020
106	H	3.57604810	7.21942843	1.69335975
107	H	4.37647439	8.02033930	0.33479139
108	H	5.00783284	6.49142682	0.95658484
109	H	3.25980220	6.63536620	-2.36444949
110	H	4.86875054	6.45577930	-1.65798414
111	H	3.84044918	5.03511934	-1.87941115
112				
113	Energy = -2864.30515561 a.u. #Cluster: 6			
114	O	6.69924489	-1.44641826	-5.23081375
115	N	4.48152792	-1.19328749	-5.44988406
116	C	5.75319024	-1.05307887	-5.92321350
117	C	5.94276370	-0.48718684	-7.27281604
118	C	7.08290234	0.12148638	-7.60124312
119	H	5.10889548	-0.60109699	-7.96818793
120	H	3.68460562	-0.78029302	-5.90497365
121	H	4.32555973	-1.55717214	-4.52346596
122	H	7.26901048	0.55105727	-8.57955229
123	H	7.91927537	0.24137413	-6.91885501
124	O	2.48510870	-3.09876391	-3.45668879

1	N	1.16044739	-3.37236565	-1.67128967	63	O	3.10575973	-0.55652485	2.12257116
2	C	1.34888586	-3.02910911	-2.97756050	64	C	1.79152055	4.56321971	-0.63803253
3	C	0.18726904	-2.58981364	-3.77194197	65	C	2.65907459	5.05117612	0.47674785
4	C	0.32612582	-1.68155856	-4.73842931	66	C	3.98567172	5.22847797	0.39957078
5	H	-0.76791304	-3.06300285	-3.53747013	67	C	4.76005078	5.70491119	1.58547524
6	H	0.26203239	-3.33938988	-1.21563494	68	C	4.79962516	4.96537384	-0.82022596
7	H	1.90862767	-3.77253087	-1.12475236	69	H	7.20766319	1.14993045	-0.89823968
8	H	-0.49794045	-1.33573825	-5.35381112	70	H	5.45467391	1.56508182	0.82859241
9	H	1.27166388	-1.21199987	-4.99190999	71	H	3.06125213	-1.63463738	-0.80729820
10	O	-2.21254542	8.56177588	0.93717697	72	H	-1.45594162	0.40381408	0.72424430
11	N	-0.58289526	7.08400171	0.50478538	73	H	-2.06511162	2.00755987	0.34500222
12	C	-1.64316044	7.51462994	1.24956819	74	H	0.38269655	0.60379787	-0.91517669
13	C	-2.03539961	6.69808181	2.41793705	75	H	1.63035255	-0.33935396	2.84261347
14	C	-3.28794399	6.70586249	2.87263279	76	H	-1.52684835	3.93619123	-0.65212568
15	H	-1.24289249	6.09508107	2.87087340	77	H	-0.59416111	4.71011531	-1.94172618
16	H	-0.12709416	6.20344775	0.70131606	78	H	-1.72522062	3.42086833	-2.31925704
17	H	-0.28571963	7.57997042	-0.31922109	79	H	0.09245757	2.72989554	-3.59019550
18	H	-3.61534925	6.12468449	3.72761691	80	H	1.55456299	3.42614052	-2.91182595
19	H	-4.08609344	7.29213934	2.42887000	81	H	-2.44426248	1.39853988	-1.89324052
20	C	6.11871360	-0.47208764	-1.80615379	82	H	-1.16886855	0.94416286	-3.00599824
21	C	6.30712608	0.53703036	-0.86540173	83	H	-0.89516185	-1.27163715	-1.96298770
22	C	5.32542770	0.76156529	0.10119836	84	H	-3.88903669	-3.02876734	-0.90514252
23	C	4.17454463	-0.01848034	0.11598337	85	H	-2.24094009	-3.21868692	-1.48512256
24	C	3.96788032	-1.03473817	-0.82109405	86	H	-3.82081259	0.67191524	-0.51925550
25	C	4.95097378	-1.26480962	-1.77241024	87	H	-4.79878386	-0.44905600	-1.47324059
26	C	3.12300341	0.18664700	1.13848200	88	H	-4.48160305	-0.78250729	0.23172016
27	C	2.08090667	1.16985776	0.94825541	89	H	-1.21708396	1.23396441	3.29892908
28	C	0.94748526	1.12830239	1.73350053	90	H	-0.40685656	2.78531179	3.55327921
29	C	-0.23659570	2.02778483	1.51665856	91	H	-2.18590137	4.01580384	2.38263281
30	C	0.28338027	3.35235856	0.96109020	92	H	-5.26702274	2.82345186	1.40413050
31	C	1.12399389	3.21632797	-0.29377314	93	H	-4.53631903	4.25460055	2.13957197
32	C	2.25328084	2.20521635	-0.06979960	94	H	-5.46895370	3.12613364	3.13259540
33	C	0.14098678	2.70596575	-1.41709634	95	H	-3.24228940	0.30079826	2.10177744
34	C	-1.12443436	1.41876913	0.41749639	96	H	-4.82043487	0.65707130	2.80263821
35	C	-0.45445810	1.34497102	-0.96451229	97	H	-3.38646996	0.63086943	3.83211292
36	O	0.78335847	0.27751267	2.71715317	98	H	-2.97623562	-2.27297785	1.37274000
37	C	-0.98049236	3.74854088	-1.58219548	99	H	-2.63048644	-3.94545566	0.93614334
38	C	0.84617098	2.58108575	-2.78800363	100	H	-0.63185862	-1.62711041	0.45994496
39	C	-1.50992088	0.80824344	-1.95842537	101	H	1.61338831	-2.80640581	2.63287961
40	C	-1.73417474	-0.63840381	-1.67610175	102	H	1.64678086	-2.13993212	0.99761475
41	C	-2.81599783	-1.16568837	-1.08852716	103	H	2.06172721	-3.84236575	1.28074798
42	C	-2.86032517	-2.63419062	-0.77706739	104	H	-1.20355256	-4.68453057	2.74153478
43	C	-4.02561304	-0.39376276	-0.69072478	105	H	-0.78996019	-5.43333010	1.19630949
44	O	3.27018767	2.29464603	-0.71369365	106	H	0.43671383	-5.26984716	2.45621450
45	O	0.00183957	4.40130292	1.47630932	107	H	2.35266606	1.05037609	-2.31198893
46	C	-1.02992459	2.22126790	2.82276507	108	H	0.90045074	0.40910731	-3.08288854
47	C	-2.30967413	2.95063219	2.58059865	109	H	1.60080550	1.12101256	-5.26992982
48	C	-3.52378424	2.38272642	2.58145607	110	H	5.01838772	1.68689892	-6.12465297
49	C	-4.74848180	3.18935109	2.30373433	111	H	4.07921170	3.17371013	-6.24627647
50	C	-3.75787180	0.93268281	2.84047371	112	H	3.37771197	1.65103822	-6.79301591
51	C	-2.38738027	-2.89735206	0.66640048	113	H	3.92904487	3.12409611	-2.92924204
52	C	-0.93480511	-2.60388474	0.83789080	114	H	5.35583750	2.64812996	-3.86959840
53	C	-0.05266168	-3.42320340	1.42710442	115	H	4.53937204	1.48770344	-2.80754442
54	C	1.37971313	-3.03110609	1.57837597	116	H	3.94500330	-2.64478598	-2.77963112
55	C	-0.41205781	-4.75956182	1.98089612	117	H	6.77721202	-1.06068117	-3.57898578
56	C	1.59641193	1.27074790	-3.09044010	118	H	2.38023955	4.44253170	-1.57622689
57	C	2.23638960	1.40737241	-4.43598463	119	H	1.02076547	5.32353685	-0.87879722
58	C	3.46785726	1.89878248	-4.63408011	120	H	2.12816179	5.26901273	1.40246704
59	C	4.00608719	2.09994701	-6.01264637	121	H	4.13301852	5.87699289	2.47026069
60	C	4.37252520	2.30904713	-3.52570520	122	H	5.28335618	6.64808046	1.37251178
61	O	4.88029608	-2.25919555	-2.68607889	123	H	5.52757612	4.97020503	1.87404279
62	O	7.10970068	-0.68270687	-2.69670940	124	H	4.36157084	5.42196761	-1.71839434

H	5.82873295	5.33617399	-0.73874368
H	4.86340136	3.87793595	-1.01281019

Energy = -2864.30426101 a.u. #Cluster: 7

O	7.50290474	-2.77772643	-3.97790477
N	5.76461012	-3.96881852	-4.73170638
C	7.10325570	-3.73296376	-4.66164578
C	8.02556887	-4.64117979	-5.36614442
C	9.33220388	-4.37818379	-5.42441781
H	7.58147211	-5.52481261	-5.82969645
H	5.36173759	-4.66612364	-5.33929568
H	5.11404715	-3.36035210	-4.26057596
H	10.04681315	-5.01749886	-5.93150275
H	9.79042666	-3.50414233	-4.97095087
O	1.85061922	-2.12329100	-4.02033183
N	3.09294281	-0.65936929	-5.19296165
C	1.88803272	-1.19648869	-4.84461277
C	0.66532962	-0.67898962	-5.48580242
C	-0.52160852	-0.82084093	-4.89407241
H	0.79163285	-0.18605631	-6.45102439
H	3.16741346	0.15388074	-5.78429209
H	3.94522531	-0.93842157	-4.72889182
H	-1.44873250	-0.45579810	-5.32206835
H	-0.65874464	-1.31353658	-3.93502528
O	-2.41778983	8.54221014	1.61744873
N	-0.70818869	7.18429133	1.10726662
C	-1.81587613	7.49116618	1.84393612
C	-2.21942955	6.53986046	2.90139472
C	-3.48784745	6.45299819	3.30032082
H	-1.42080624	5.92176706	3.32182131
H	-0.22196269	6.30648230	1.23120743
H	-0.39871165	7.77692452	0.35467754
H	-3.82418144	5.77198326	4.07422455
H	-4.29145336	7.05404905	2.88729005
C	5.42274538	-0.42250049	-2.51311520
C	5.79952591	0.67171776	-1.74003722
C	5.10014619	0.94575409	-0.56316127
C	4.04112442	0.12919876	-0.17935929
C	3.67833696	-0.99469999	-0.92713106
C	4.38570686	-1.27516511	-2.08803814
C	3.23066928	0.40607648	1.02852789
C	2.16302555	1.38548664	0.98731301
C	1.09771351	1.25849510	1.85097885
C	-0.16972732	2.05105311	1.69861802
C	0.24087178	3.44184358	1.21513817
C	1.02327867	3.44097340	-0.08517608
C	2.22545317	2.49373376	0.03465886
C	0.00317764	2.94622277	-1.18071978
C	-1.01302660	1.39666260	0.58848098
C	-0.41006539	1.49288469	-0.82313664
O	1.06820849	0.40623294	2.84944030
C	-1.22501535	3.87912672	-1.14564038
C	0.57365977	3.03966414	-2.61297961
C	-1.44675330	0.91984035	-1.81370204
C	-1.70593817	-0.51454851	-1.49317359
C	-2.86513328	-0.99618106	-1.02499520
C	-3.00620346	-2.45640389	-0.70973603
C	-4.07483734	-0.15901646	-0.77986592
O	3.21956139	2.70605707	-0.61626339
O	-0.05819014	4.43319180	1.82561430
C	-0.95997248	2.11073799	3.01889341
C	-2.28792296	2.76480349	2.82549414
C	-3.46141617	2.11848125	2.78178258
C	-4.73570989	2.85851486	2.54409442
C	-3.60226983	0.64224852	2.93849732
C	-2.75390926	-2.71990891	0.78836864
C	-1.34160880	-2.42323412	1.17525118
C	-0.28171332	-3.17237330	0.84181872
C	1.09622121	-2.80034431	1.28451494
C	-0.35756129	-4.41540402	0.02291938
C	1.51480418	1.91722089	-3.07289600
C	1.94231520	2.20855416	-4.47578770
C	3.16043683	2.63280410	-4.83708161
C	3.49304049	2.87626487	-6.27395178
C	4.28010564	2.87833743	-3.88712691
O	4.13384956	-2.39359683	-2.81131898
O	5.99055877	-0.58975724	-3.73109437
O	3.38830092	-0.29578016	2.02779330
C	1.58415635	4.84356749	-0.38506729
C	2.48806356	5.33256676	0.69952960
C	3.77628870	5.66103566	0.52549639
C	4.60496758	6.13965152	1.67241959
C	4.48794250	5.57750313	-0.78153596
H	6.61651146	1.31446735	-2.06718061
H	5.37252860	1.81663368	0.03871457
H	2.85263706	-1.63743478	-0.62316285
H	-1.19309034	0.32812817	0.83873616
H	-2.02604217	1.85430088	0.58568035
H	0.50186065	0.84594863	-0.87323272
H	1.96869750	-0.12550766	2.93448077
H	-1.72725476	3.89301352	-0.17306516
H	-0.95839433	4.91300646	-1.39520494
H	-1.98171904	3.56870453	-1.87807991
H	-0.27636311	3.08647261	-3.32798243
H	1.09378383	4.01073315	-2.74049521
H	-2.37597098	1.52197953	-1.79204594
H	-1.07769598	1.01066092	-2.85699701
H	-0.84798972	-1.17145716	-1.64031671
H	-4.01606840	-2.82000439	-0.98576680
H	-2.30133206	-3.06276220	-1.31470890
H	-3.83807476	0.75383018	-0.21411244
H	-4.53499599	0.16209218	-1.72660022
H	-4.84953812	-0.68911285	-0.20972775
H	-1.07350489	1.08570730	3.43454850
H	-0.37040655	2.67134915	3.77903939
H	-2.23709335	3.84684645	2.70018635
H	-5.23669215	2.50044494	1.63142288
H	-4.59173639	3.94087041	2.42487733
H	-5.44558694	2.71593689	3.37215117
H	-3.19038229	0.10736817	2.06791794
H	-4.64828737	0.32135243	3.03618005
H	-3.07112096	0.26680738	3.82613799
H	-3.46002788	-2.11361558	1.39116257
H	-3.00710931	-3.77632064	1.01938628
H	-1.21915051	-1.51762503	1.76649552
H	1.18217421	-1.74339729	1.56490545
H	1.84192327	-2.99842638	0.50093926
H	1.39748870	-3.39309036	2.16286071
H	-1.34189083	-4.90045405	0.07385434
H	-0.16732163	-4.19241224	-1.03935318
H	0.38629998	-5.16331537	0.33117374
H	2.38689698	1.81886119	-2.39888507
H	1.00697544	0.93155111	-3.03531068
H	1.15806900	2.05753296	-5.21646311

H	4.40250574	2.33526664	-6.57547495
H	3.68545071	3.94327685	-6.46105267
H	2.69324166	2.57352882	-6.96197452
H	3.92750565	3.10714062	-2.86703349
H	4.91879495	3.71343912	-4.20056144
H	4.92414521	1.98765942	-3.80382180
H	3.17518379	-2.37509384	-3.19981995
H	6.48330704	-1.47839253	-3.80636697
H	2.12227880	4.80903272	-1.36100602
H	0.75494636	5.56395710	-0.54064775
H	2.02372785	5.41680349	1.68036804
H	4.04372309	6.20412205	2.61402418
H	5.02427110	7.13726707	1.47742220
H	5.45588444	5.46445282	1.85126155
H	4.00463141	6.19655416	-1.55047506
H	5.53593657	5.89506228	-0.72271289
H	4.48765947	4.53891368	-1.16015876

Energy = -2864.29981188 a.u. #Cluster: 8

O	3.72196426	-3.02959489	-5.22221384
N	3.99670223	-0.89344684	-5.85733614
C	4.28554387	-2.22351693	-5.97437514
C	5.20677271	-2.65153013	-7.04337066
C	5.76695807	-3.86168406	-7.01532536
H	5.40057211	-1.92857020	-7.83842998
H	4.50639999	-0.18928760	-6.36684539
H	3.44250239	-0.55366390	-5.08364702
H	6.45361843	-4.21812187	-7.77549904
H	5.58623688	-4.58927072	-6.22940324
O	-1.61127957	8.49774636	0.06639659
N	0.06398636	7.20462797	0.81251809
C	-1.25817582	7.42478989	0.55627248
C	-2.20627916	6.34638828	0.91374075
C	-3.24610276	6.05192957	0.13576506
H	-1.98809724	5.81711052	1.84513892
H	0.39319107	6.31098602	1.15594013
H	0.76895515	7.87440762	0.55534166
H	-3.95995175	5.26852220	0.36407721
H	-3.46956374	6.56601435	-0.79307840
O	7.30989826	4.46259103	1.30535216
N	5.48157211	3.18400336	1.52084667
C	6.36510494	3.89499022	0.75638687
C	6.14711926	3.90664990	-0.70453831
C	6.63839948	4.88379760	-1.46542828
H	5.54500339	3.08680521	-1.10755728
H	4.66461006	2.76377146	1.11143356
H	5.53487169	3.19038359	2.52588698
H	6.49115188	4.93493355	-2.53822715
H	7.22276251	5.71092057	-1.07752009
C	4.95031340	-1.06416306	-2.39714334
C	5.71969367	-0.30153981	-1.52930778
C	5.16955462	0.11139076	-0.31255737
C	3.85991645	-0.22538625	-0.00100626
C	3.07396092	-0.99485176	-0.86553773
C	3.62345644	-1.42552438	-2.06771728
C	3.22268477	0.21417901	1.26149456
C	2.18235594	1.22682507	1.18591715
C	1.13272238	1.20212811	2.07254228
C	-0.11701364	2.01154122	1.86465807
C	0.31269428	3.32796958	1.21360301
C	1.05678631	3.15719596	-0.10284275
C	2.26736613	2.25345245	0.15101847
C	0.02413732	2.52411545	-1.11017061
C	-1.00231500	1.25684108	0.85640867
C	-0.39971421	1.13929883	-0.55413337
O	1.09378765	0.43334524	3.13832024
C	-1.19596052	3.46313850	-1.19769592
C	0.58367417	2.41788792	-2.54587596
C	-1.43205296	0.42348797	-1.45261022
C	-1.65092970	-0.95898496	-0.93373221
C	-2.75084235	-1.38214248	-0.29692592
C	-2.81945058	-2.78095453	0.24437360
C	-3.96778407	-0.55305476	-0.06734996
O	3.30526197	2.45102788	-0.43956077
O	0.07996524	4.39044120	1.72252214
C	-0.86252944	2.24140138	3.19083196
C	-2.16923385	2.92771010	2.96881645
C	-3.36647417	2.32704743	3.02259912
C	-4.62043657	3.08978030	2.75321664
C	-3.55165196	0.88013359	3.33236657
C	-2.34048345	-2.82088008	1.70993322
C	-0.87863264	-2.53531617	1.83074979
C	0.09711987	-3.38553712	1.48303128
C	1.53686461	-3.02546741	1.65133366
C	-0.13955651	-4.73739627	0.90084955
C	1.55620144	1.26629101	-2.84198126
C	2.12822537	1.47936773	-4.20807529
C	3.30132469	2.08515899	-4.44113043
C	3.78274190	2.34606488	-5.83002501
C	4.20081296	2.56435581	-3.35533086
O	2.82902039	-2.17100601	-2.85534356
O	5.40394652	-1.51697473	-3.59175968
O	3.48686178	-0.33650030	2.32229416
C	1.60081249	4.50692945	-0.59575548
C	2.50393559	5.17677287	0.38825447
C	3.24910776	6.24648717	0.07371811
C	4.13771663	6.89770846	1.07879492
C	3.25966146	6.84863828	-1.29009341
H	6.74159264	-0.02731164	-1.79451670
H	5.77293011	0.70205496	0.37808098
H	2.04429567	-1.25850802	-0.62272146
H	-1.22882036	0.24080370	1.24768261
H	-1.99271430	1.75748424	0.79743996
H	0.51021336	0.48880789	-0.51154285
H	1.98132837	-0.08397803	3.28210591
H	-1.64813797	3.67435644	-0.22327976
H	-0.93671772	4.42654657	-1.65358657
H	-1.98791258	3.02833933	-1.82199069
H	-0.26962028	2.33451346	-3.25333358
H	1.07251247	3.37540715	-2.81874199
H	-2.37303879	1.00405870	-1.50338073
H	-1.06767736	0.38078232	-2.50097265
H	-0.79974242	-1.62714393	-1.07305995
H	-3.85325222	-3.17524971	0.18264259
H	-2.20378622	-3.47416676	-0.36441549
H	-3.75317327	0.52315354	-0.02084921
H	-4.69991391	-0.69953258	-0.87641334
H	-4.47689915	-0.81410751	0.87169239
H	-1.00294726	1.27121850	3.71633337
H	-0.22726568	2.85092913	3.87279407
H	-2.08166093	3.99025012	2.74275161
H	-5.11067039	2.72864590	1.83524156
H	-4.45313565	4.16744214	2.62374473
H	-5.34998110	2.97306616	3.56806282

H	-3.03687443	0.23958601	2.59996869
H	-4.60648450	0.57358834	3.33107679
H	-3.14694610	0.62131604	4.32327077
H	-2.92984261	-2.09793945	2.30936219
H	-2.57796679	-3.81642712	2.14196242
H	-0.64394720	-1.55209811	2.23427642
H	1.68634276	-1.98860495	1.97449304
H	2.09741493	-3.16514310	0.71444221
H	2.01927060	-3.66626328	2.40582697
H	-1.10085183	-5.17033359	1.21061991
H	-0.15174952	-4.68922492	-0.20002588
H	0.64238536	-5.45636256	1.18157103
H	2.35800290	1.21120638	-2.08084957
H	1.03814468	0.28706211	-2.79087732
H	1.49282358	1.13848578	-5.02189008
H	4.81976208	2.00963758	-5.97682148
H	3.77866859	3.42694551	-6.04466399
H	3.16416852	1.87168572	-6.60298006
H	3.67952029	3.26450279	-2.68023570
H	5.09606969	3.07431439	-3.72969771
H	4.53747283	1.72894932	-2.71754154
H	3.26428497	-2.44369032	-3.73616271
H	6.30215878	-1.14060405	-3.81819092
H	2.15643156	4.34246220	-1.55119680
H	0.76110075	5.18407505	-0.86272956
H	2.53352426	4.74738031	1.38581942
H	4.07641836	6.43275945	2.07208809
H	3.89421922	7.96314420	1.20391583
H	5.19157512	6.84392627	0.76528211
H	2.24465657	7.06173417	-1.65592638
H	3.82378763	7.78934173	-1.33877172
H	3.72234758	6.16297941	-2.01803081

OC-(AM)$_4$ complex in the DMSO

Energy = -3111.43796223 a.u. #Cluster: 1

O	3.93064285	-2.74749999	-4.39620286
N	4.46730677	-0.61147785	-4.80674914
C	4.68934159	-1.95129742	-4.96431186
C	5.79935281	-2.38208945	-5.83222358
C	6.15025735	-3.66762714	-5.90039690
H	6.30964935	-1.60150646	-6.40035311
H	5.04455450	0.09090762	-5.23071250
H	3.72635289	-0.27740742	-4.20539866
H	6.96292440	-4.03220477	-6.51954769
H	5.65459278	-4.45525348	-5.34014413
O	2.29788589	-0.77644790	4.44480437
N	4.22696091	-1.49650794	3.55612514
C	3.04936958	-1.72855801	4.19873815
C	2.73043136	-3.10297988	4.62923447
C	1.46087083	-3.49316772	4.75199562
H	3.57823342	-3.75923485	4.83171692
H	4.84744628	-2.23504941	3.26916933
H	4.45207862	-0.56412531	3.23354849
H	1.16984921	-4.49031080	5.06290123
H	0.61308344	-2.84667475	4.54115669
O	-2.75379983	8.76897267	0.33748563
N	-0.95464875	7.43179914	0.27936165
C	-2.18137222	7.76919659	0.77477684
C	-2.74749723	6.91493914	1.84073820
C	-4.06390931	6.83656601	2.03140239
H	-2.02541593	6.36152709	2.44800526
H	-0.48112815	6.58934101	0.57717796
H	-0.52706950	7.95713654	-0.46506613
H	-4.51999068	6.22765328	2.80383610
H	-4.79193401	7.37457706	1.43277782
O	7.86047382	3.25790122	-0.59185982
N	5.85056174	3.31785811	0.39319998
C	6.66318020	2.97062505	-0.64795910
C	6.05112246	2.24852425	-1.78301549
C	6.76960133	1.92285616	-2.85740219
H	4.99007109	1.99806170	-1.68270187
H	4.85953750	3.13128345	0.37180797
H	6.19970629	3.83425505	1.18140398
H	6.36415360	1.39303362	-3.70926740
H	7.82268621	2.15907501	-2.96970209
C	5.04643949	-1.33227553	-1.31328816
C	5.84919127	-0.75774094	-0.33474660
C	5.26275712	0.05450843	0.63530757
C	3.88990226	0.29174709	0.61324301
C	3.07434975	-0.29994766	-0.35753434
C	3.64839936	-1.11571285	-1.32390967
C	3.28742540	1.15835127	1.64383461
C	2.04926383	1.87511753	1.33242747
C	0.94556115	1.75502705	2.12840362
C	-0.36083576	2.43970096	1.80107985
C	-0.02555846	3.72843049	1.05397986
C	0.80125527	3.52435299	-0.20607634
C	2.06823740	2.74700648	0.16479224
C	-0.13620888	2.73108373	-1.19530833
C	-1.13679076	1.52935815	0.83406573
C	-0.46996015	1.36252194	-0.54215657
O	0.84267086	1.00652188	3.19687806
C	-1.42468579	3.55502217	-1.39537403
C	0.48098573	2.55908735	-2.60083336
C	-1.39817829	0.49034113	-1.41772502
C	-1.47146412	-0.87490432	-0.81831885
C	-2.48979086	-1.35243380	-0.09077050
C	-2.37742223	-2.69325992	0.57794032
C	-3.77343227	-0.63760471	0.15449043
O	3.08393490	2.90670632	-0.47741015
O	-0.40623208	4.80078541	1.44054008
C	-1.17526556	2.72476706	3.07622890
C	-2.56133769	3.16967424	2.74456924
C	-3.66266395	2.43082755	2.93844861
C	-5.01072324	2.94204407	2.55386187
C	-3.64517362	1.06443423	3.53670937
C	-1.57421518	-2.56959385	1.89107138
C	-0.11958930	-2.37514538	1.60917220
C	0.74944266	-3.37490503	1.40724702
C	2.18399989	-3.10423040	1.09662502
C	0.37329602	-4.81722323	1.46959568
C	1.54515494	1.46685553	-2.77923588
C	2.00186227	1.45827361	-4.20372692
C	2.91181603	2.29924009	-4.71510805
C	3.30546574	2.23173018	-6.15480486
C	3.58192315	3.37797569	-3.93691934
O	2.81076853	-1.67135884	-2.21964761
O	5.52722807	-2.12040216	-2.30038880
O	3.79357443	1.25371729	2.74837225
C	1.26310513	4.87787998	-0.77740892
C	2.10809400	5.63478400	0.19572206
C	3.34515437	6.08525213	-0.05750277
C	4.11975733	6.82297519	0.98459402

C	4.05201879	5.88995318	-1.35499731
H	6.92393841	-0.93963594	-0.32874295
H	5.88790077	0.51290197	1.40630510
H	1.99339889	-0.14238657	-0.36458909
H	-1.28101875	0.53119756	1.30285276
H	-2.16856954	1.91946034	0.70387898
H	0.49005107	0.79990566	-0.41096335
H	1.66345824	0.44261157	3.48757654
H	-1.95017993	3.76927483	-0.45953090
H	-1.22262492	4.51527801	-1.88525140
H	-2.14010181	3.02360781	-2.03659647
H	-0.33890801	2.35519157	-3.32345587
H	0.90594675	3.52720808	-2.93614580
H	-2.39558879	0.96055471	-1.51464817
H	-1.01080129	0.43072400	-2.45606668
H	-0.57077637	-1.47415066	-0.96583739
H	-3.37689206	-3.11730837	0.79468700
H	-1.88685353	-3.42947373	-0.09011108
H	-3.80232226	0.37180205	-0.27743547
H	-4.62445432	-1.19119531	-0.27049154
H	-3.97317588	-0.52885386	1.23116661
H	-1.18904918	1.82354068	3.72723008
H	-0.66158122	3.50840556	3.67822278
H	-2.61891733	4.16589567	2.30644300
H	-5.45622452	2.32281030	1.75981785
H	-4.99260527	3.97370492	2.17836248
H	-5.70965532	2.91851512	3.40254652
H	-2.88575786	0.41942823	3.07006586
H	-4.60760034	0.54425752	3.44022826
H	-3.41273739	1.10314611	4.61285076
H	-1.96994546	-1.72768221	2.49377794
H	-1.74148729	-3.47370575	2.51087382
H	0.19471352	-1.33500486	1.55855540
H	2.49833626	-2.09323757	1.38618074
H	2.37308172	-3.19944990	0.01464325
H	2.85653065	-3.80956800	1.60352204
H	0.30694648	-5.15837550	2.51419401
H	-0.60459858	-5.01373937	1.00778476
H	1.10205745	-5.46663022	0.96700749
H	2.40518783	1.63185956	-2.10005850
H	1.14229330	0.46954147	-2.50613793
H	1.51734330	0.70848508	-4.82649628
H	4.39715849	2.21797115	-6.28399340
H	2.94006584	3.11382649	-6.70300027
H	2.90642563	1.34938518	-6.67226986
H	3.45301412	3.25945679	-2.84742980
H	3.16732923	4.36401987	-4.19785858
H	4.66214727	3.42291976	-4.13389527
H	3.29460555	-2.13107019	-2.98849608
H	6.52352581	-2.20286330	-2.26790400
H	1.82027317	4.69379036	-1.72456218
H	0.38589691	5.48838494	-1.07576217
H	1.64672827	5.81305554	1.16562487
H	3.56541092	6.95320808	1.92367506
H	4.40620443	7.82650981	0.63723507
H	5.05207573	6.29323543	1.23285456
H	3.47358786	6.28520687	-2.20214691
H	5.03644630	6.37388251	-1.38703417
H	4.21617687	4.81623883	-1.55054663
Energy = -3111.43611127 a.u. #Cluster: 2			
O	1.31107505	-2.80033110	-3.62388231
N	2.93567481	-1.50657900	-4.45206187
C	1.62093763	-1.86195618	-4.36887375
C	0.63299849	-1.14154212	-5.19179645
C	-0.65953058	-1.16020435	-4.86188979
H	1.01636553	-0.60650188	-6.06158289
H	3.25314194	-0.69524704	-4.95791964
H	3.62372768	-1.96949489	-3.87765639
H	-1.42785370	-0.64676547	-5.42911414
H	-1.04757027	-1.68925355	-3.99392806
O	3.03810432	-0.32848661	4.24632856
N	5.00324169	-0.93463276	3.34880126
C	3.92952405	-1.18143911	4.14707906
C	3.89748890	-2.43915751	4.91890853
C	2.87431269	-2.71443774	5.72964131
H	4.74495310	-3.11495937	4.78867059
H	5.72059669	-1.61944753	3.16762694
H	5.05369868	-0.08036735	2.81338479
H	2.80609603	-3.62347295	6.31693608
H	2.02492922	-2.05125236	5.86896681
O	-3.18189682	8.48330795	0.67972430
N	-1.30722131	7.29357877	0.36500468
C	-2.49432652	7.51320619	1.00274138
C	-2.87762382	6.57252983	2.07734569
C	-4.15746847	6.36201109	2.38230630
H	-2.05324100	6.06801735	2.59011863
H	-0.74749319	6.47601727	0.56621440
H	-1.00487027	7.87983306	-0.39517801
H	-4.48078256	5.68344186	3.16375101
H	-4.98487926	6.84952117	1.87733903
O	8.07268285	3.66005652	0.86623554
N	5.83371278	3.56865445	0.80819889
C	7.04208264	3.68156919	0.19053076
C	7.05105645	3.84868393	-1.27929593
C	7.95481697	3.22596045	-2.03402196
H	6.28333402	4.50648452	-1.69016648
H	4.96543015	3.51402419	0.29404894
H	5.76501869	3.40218045	1.80055140
H	8.00209332	3.32370819	-3.11283362
H	8.71911046	2.56568347	-1.63871085
C	4.44847798	-1.87424013	-1.39210181
C	5.44993024	-0.98657966	-1.01267844
C	5.14451255	0.03337464	-0.11265344
C	3.84679147	0.17159152	0.37512849
C	2.84001601	-0.72864589	0.00997054
C	3.14329215	-1.76729851	-0.85863219
C	3.54008813	1.30205478	1.27691306
C	2.25255100	1.97852533	1.07182653
C	1.15552805	1.72067489	1.83326006
C	-0.20420298	2.29527898	1.51339224
C	0.02763185	3.65090154	0.85032915
C	0.90742045	3.60283151	-0.38953024
C	2.23288682	2.93427055	-0.02976625
C	0.11761528	2.76643588	-1.46500704
C	-0.88638415	1.39623350	0.46888474
C	-0.16612507	1.35250009	-0.88708839
O	1.11703980	0.92429402	2.87827263
C	-1.20275821	3.50404757	-1.75558978
C	0.89125640	2.67381642	-2.80173352
C	-1.02750584	0.50225587	-1.84414682
C	-1.09477496	-0.90506204	-1.35211864
C	-2.21866174	-1.51841060	-0.95921112
C	-2.18206263	-2.93648698	-0.47257431

1	C	-3.56359570	-0.87107730	-0.97982901
2	O	3.24667150	3.21824453	-0.62995943
3	O	-0.45875840	4.66123485	1.28270419
4	C	-1.06803225	2.41721478	2.78247618
5	C	-2.46560204	2.82247130	2.44924782
6	C	-3.52063680	1.99658247	2.41721324
7	C	-4.87384914	2.49748548	2.03268079
8	C	-3.45539820	0.53881930	2.72236396
9	C	-2.32517176	-3.00324963	1.06074920
10	C	-1.12373206	-2.45117683	1.75669777
11	C	0.06120128	-3.07113652	1.84014130
12	C	1.20731470	-2.45714159	2.57404830
13	C	0.36801373	-4.38743213	1.21185802
14	C	1.93104333	1.54922711	-2.92010655
15	C	2.63870349	1.66366385	-4.23095315
16	C	3.96659293	1.77777300	-4.37020540
17	C	4.59740439	1.84940782	-5.72127538
18	C	4.91241223	1.83454882	-3.22067373
19	O	2.19534251	-2.70194460	-1.09720156
20	O	4.64112939	-2.86031369	-2.29623707
21	O	4.32415426	1.65451958	2.13375472
22	C	1.22961605	5.03367199	-0.86206188
23	C	2.06192075	5.76548083	0.14043669
24	C	3.27963035	6.26780829	-0.10963440
25	C	4.05922808	6.96022376	0.95907766
26	C	3.95538196	6.17846941	-1.43605197
27	H	6.45941958	-1.09001276	-1.41205042
28	H	5.92587013	0.73067916	0.20029945
29	H	1.82256784	-0.64076694	0.40050689
30	H	-0.97950874	0.36238346	0.86995952
31	H	-1.93428851	1.73382126	0.32214705
32	H	0.81529183	0.83130464	-0.75084825
33	H	2.00715456	0.52381578	3.21172994
34	H	-1.80832087	3.65755715	-0.85623300
35	H	-1.02980274	4.48628127	-2.21156711
36	H	-1.82732035	2.94414650	-2.46371311
37	H	0.16203647	2.56106928	-3.63094417
38	H	1.39693305	3.64052074	-3.00436458
39	H	-2.03521099	0.94946579	-1.95324089
40	H	-0.60141692	0.52019818	-2.86917102
41	H	-0.13242773	-1.42068914	-1.31851913
42	H	-2.99078418	-3.52592150	-0.94871553
43	H	-1.23638935	-3.43390838	-0.77347859
44	H	-3.55629724	0.10468938	-0.47301701
45	H	-3.90325544	-0.69279164	-2.01113371
46	H	-4.33576810	-1.47783336	-0.48905977
47	H	-1.05118722	1.45470689	3.34030567
48	H	-0.61048938	3.16231872	3.47111168
49	H	-2.57409150	3.87924419	2.20220043
50	H	-5.28645239	1.92359760	1.18923186
51	H	-4.87402407	3.55323307	1.72988879
52	H	-5.58939938	2.39966714	2.86240502
53	H	-3.18974996	-0.03917073	1.82193641
54	H	-4.41578253	0.14028973	3.07780037
55	H	-2.70691076	0.29661324	3.49101491
56	H	-3.23937409	-2.45821847	1.37090703
57	H	-2.49755904	-4.05766032	1.36315570
58	H	-1.27173176	-1.47064188	2.20342824
59	H	0.98508775	-1.45244844	2.95776439
60	H	2.09198438	-2.37663729	1.92411996
61	H	1.50239260	-3.07106962	3.43742701
62	H	-0.52604067	-4.99894979	1.03534019
63	H	0.85061127	-4.22856029	0.23065795
64	H	1.06259538	-4.98711878	1.81405468
65	H	2.64213794	1.57727951	-2.07225931
66	H	1.44372693	0.55267382	-2.86049596
67	H	1.97983647	1.64859984	-5.09767309
68	H	5.34504161	1.05289347	-5.85687855
69	H	5.12544127	2.80412583	-5.86419547
70	H	3.87572138	1.75634981	-6.54320872
71	H	4.59973667	2.59182832	-2.48075458
72	H	5.93807482	2.07968961	-3.52309581
73	H	4.95499975	0.87242055	-2.68815813
74	H	1.96745375	-2.76815610	-2.08082297
75	H	5.59506151	-2.91583071	-2.60615900
76	H	1.74790749	4.98068708	-1.84661678
77	H	0.29246088	5.59198679	-1.06316605
78	H	1.60841435	5.86402096	1.12518343
79	H	3.49022227	7.09597225	1.88853704
80	H	4.39573534	7.95568687	0.63575552
81	H	4.96223367	6.38455275	1.21699827
82	H	3.42084637	6.76165696	-2.20022888
83	H	4.98861513	6.54768628	-1.41863980
84	H	3.98503301	5.13645961	-1.79835081
85				
86	Energy = -3111.43031752 a.u. #Cluster: 3			
87	O	5.98820480	-3.22434260	-4.56109367
88	N	3.92552495	-4.08985574	-4.34363896
89	C	5.07601962	-3.89084652	-5.05796655
90	C	5.21969793	-4.57805600	-6.35766222
91	C	5.98617885	-4.05594900	-7.31527703
92	H	4.66341322	-5.50826706	-6.47950904
93	H	3.11270769	-4.52386036	-4.75334072
94	H	3.74864635	-3.53329829	-3.51866935
95	H	6.11973115	-4.51219958	-8.28908119
96	H	6.53514155	-3.12602896	-7.19984209
97	O	4.09506554	-1.34877153	-5.54085382
98	N	1.86876553	-1.15543677	-5.74624083
99	C	3.12482821	-1.11851176	-6.27863999
100	C	3.28805918	-0.84433946	-7.71706778
101	C	4.50433526	-0.64836210	-8.22945562
102	H	2.37784340	-0.81280116	-8.31736013
103	H	1.04461293	-0.90398792	-6.26510392
104	H	1.74517817	-1.29324569	-4.75347942
105	H	4.68457975	-0.44303971	-9.27839095
106	H	5.41302953	-0.67952659	-7.63311334
107	O	-1.68264213	8.61245986	0.50522588
108	N	0.01188743	7.28638219	1.14078368
109	C	-1.31697354	7.51735192	0.93321554
110	C	-2.25500995	6.42218522	1.26387755
111	C	-3.33415183	6.18626439	0.52006903
112	H	-1.99701492	5.82957604	2.14584434
113	H	0.35108522	6.37878114	1.43460027
114	H	0.70944782	7.96995950	0.89894083
115	H	-4.04215487	5.39234490	0.72940899
116	H	-3.59849194	6.76296536	-0.35998590
117	O	7.10608321	4.98878795	0.98480631
118	N	5.51908093	3.46633874	1.42592598
119	C	6.22699397	4.24384572	0.55129442
120	C	5.92197537	4.10152306	-0.88822947
121	C	6.11847785	5.11246615	-1.73301201
122	H	5.50604408	3.13689749	-1.19709164
123	H	4.74092521	2.91200338	1.10757727
124	H	5.62575173	3.56822295	2.42071875

1	H	5.89728590	5.05701374	-2.79274534
2	H	6.51160007	6.07735081	-1.43348381
3	C	5.43474070	-0.62105373	-2.18392238
4	C	6.09223831	0.01661629	-1.13479864
5	C	5.38414501	0.32441613	0.02787746
6	C	4.03097222	0.02158947	0.11603483
7	C	3.35257201	-0.60519414	-0.93358371
8	C	4.06099952	-0.93307997	-2.08318351
9	C	3.24891675	0.32986309	1.33300947
10	C	2.18405716	1.31456586	1.26586792
11	C	1.12268591	1.23383673	2.13891236
12	C	-0.12208400	2.06242804	1.98096174
13	C	0.31069295	3.40863219	1.39619512
14	C	1.04906645	3.30199552	0.07102127
15	C	2.25629052	2.37680612	0.26705243
16	C	0.00296357	2.73309559	-0.96025900
17	C	-1.01952598	1.36614096	0.94259816
18	C	-0.42382224	1.32269706	-0.47516171
19	O	1.07242418	0.39730620	3.14906021
20	C	-1.21422957	3.67944251	-0.98638376
21	C	0.55079983	2.70229150	-2.40403568
22	C	-1.46190450	0.65441690	-1.40344369
23	C	-1.61202768	-0.77467870	-0.99940129
24	C	-2.65882361	-1.29144656	-0.34300851
25	C	-2.64146035	-2.72900988	0.09101537
26	C	-3.89232428	-0.53773936	0.01887139
27	O	3.27881033	2.58604863	-0.34552847
28	O	0.08017663	4.44471830	1.95840928
29	C	-0.85493662	2.22744222	3.32410637
30	C	-2.16005670	2.93035507	3.15063131
31	C	-3.35999478	2.33432966	3.19415540
32	C	-4.61314754	3.11547596	2.97912862
33	C	-3.54932164	0.87623638	3.44309304
34	C	-1.99508971	-2.86187346	1.48562050
35	C	-0.52645087	-2.58672923	1.44763978
36	C	0.39370674	-3.42258351	0.94709672
37	C	1.84652641	-3.07615063	0.95212431
38	C	0.07621777	-4.74331207	0.33240710
39	C	1.49691493	1.55070385	-2.76842897
40	C	1.94472668	1.73093764	-4.18402477
41	C	3.15707966	2.17015502	-4.54674215
42	C	3.53575643	2.29707131	-5.98641804
43	C	4.22894711	2.53479904	-3.57780782
44	O	3.39019891	-1.58750286	-3.04663821
45	O	6.15923299	-0.87319029	-3.29308411
46	O	3.44285809	-0.30556545	2.36575386
47	C	1.60406168	4.66952412	-0.35786135
48	C	2.48384032	5.30196554	0.67049224
49	C	3.18928998	6.41726657	0.43140864
50	C	4.06351603	7.02131637	1.47762230
51	C	3.17664199	7.11133834	-0.88791385
52	H	7.14974601	0.26272860	-1.22782850
53	H	5.90047562	0.80165672	0.86242355
54	H	2.28953477	-0.83520654	-0.87500753
55	H	-1.24760943	0.33130540	1.27907839
56	H	-2.00830454	1.87239687	0.91741313
57	H	0.48456477	0.66824238	-0.46898127
58	H	1.96074982	-0.14101323	3.25800294
59	H	-1.65731717	3.84160507	0.00142671
60	H	-0.95642974	4.66366629	-1.39638095
61	H	-2.01351062	3.27799242	-1.62330018
62	H	-0.30949362	2.67487764	-3.10732344
63	H	1.05765023	3.66269901	-2.62843477
64	H	-2.42318209	1.20207708	-1.37364713
65	H	-1.13522493	0.71590424	-2.46269772
66	H	-0.74989734	-1.39844308	-1.24195574
67	H	-3.66704210	-3.14684063	0.11589442
68	H	-2.08706219	-3.35646248	-0.63589888
69	H	-3.74169130	0.54965419	0.04982442
70	H	-4.69470304	-0.72959429	-0.71044248
71	H	-4.28141364	-0.83392195	1.00405059
72	H	-0.99607100	1.23218906	3.80027731
73	H	-0.21022122	2.79806475	4.03038878
74	H	-2.06930714	4.00156637	2.97152535
75	H	-5.10241448	2.81983708	2.03734071
76	H	-4.44451597	4.19938785	2.92556863
77	H	-5.34397545	2.94224225	3.78249001
78	H	-2.98692237	0.26214159	2.72386457
79	H	-4.59954665	0.56256908	3.37049781
80	H	-3.20061881	0.58969594	4.44776530
81	H	-2.50258779	-2.17436725	2.19224304
82	H	-2.18985431	-3.88171222	1.87995220
83	H	-0.23985687	-1.62180868	1.86105882
84	H	2.05318041	-2.09171286	1.38885574
85	H	2.25618296	-3.07821637	-0.07012899
86	H	2.42964959	-3.81015017	1.52885783
87	H	-0.86242773	-5.17372783	0.70708495
88	H	-0.03368663	-4.64464979	-0.75972112
89	H	0.86529958	-5.48744382	0.50715426
90	H	2.36261736	1.50917319	-2.07897446
91	H	0.99060618	0.56985955	-2.66128183
92	H	1.19179967	1.47699277	-4.92629996
93	H	4.33599860	1.58349724	-6.23949376
94	H	3.91006223	3.30247122	-6.22313629
95	H	2.70183208	2.09414846	-6.67019866
96	H	3.81920798	3.01910566	-2.67482388
97	H	4.97547157	3.21303719	-4.00797523
98	H	4.76290681	1.63158171	-3.23857595
99	H	3.76945627	-1.43408311	-4.00094766
100	H	5.83487967	-1.67549154	-3.82082175
101	H	2.18325159	4.53965994	-1.30571556
102	H	0.77029182	5.35507155	-0.62096844
103	H	2.53317881	4.80462665	1.63530948
104	H	3.95115279	6.54218914	2.45977443
105	H	3.85715940	8.09246507	1.61459945
106	H	5.12552550	6.92941983	1.19859239
107	H	2.15499303	7.28632113	-1.25518176
108	H	3.68135250	8.08605495	-0.86415501
109	H	3.68895014	6.50475112	-1.65290436
110				
